# Chitin-mediated blockade of chitinase-like proteins reduces tumor immunosuppression, inhibits lymphatic metastasis and enhances anti-PD-1 efficacy in complementary TNBC models

**DOI:** 10.1186/s13058-024-01815-8

**Published:** 2024-04-11

**Authors:** Robbe Salembier, Caro De Haes, Julie Bellemans, Kristel Demeyere, Wim Van Den Broeck, Niek N. Sanders, Steven Van Laere, Traci R. Lyons, Evelyne Meyer, Jonas Steenbrugge

**Affiliations:** 1 Laboratory of Biochemistry, Department of Veterinary and Biosciences, Faculty of Veterinary Medicine, Ghent University, Merelbeke, Belgium; 2 Cancer Research Institute Ghent (CRIG), Ghent, Belgium; 3 Department of Morphology, Imaging, Orthopedics, Rehabilitation and Nutrition, Faculty of Veterinary Medicine, Ghent University, Merelbeke, Belgium; 4 Laboratory of Gene Therapy, Department of Veterinary and Biosciences, Faculty of Veterinary Medicine, Ghent University, Merelbeke, Belgium; 5 Center for Oncological Research (CORE), Faculty of Medicine and Health Sciences, University of Antwerp, Wilrijk, Belgium; 6 Department of Medicine, Division of Medical Oncology, University of Colorado Anschutz Medical Campus, Aurora, CO USA; 7 University of Colorado Cancer Center Young Women’s Breast Cancer Translational Program, Aurora, CO USA

**Keywords:** Chitinase-like proteins, Chitin, Immunosuppression, Triple-negative breast cancer, Lymphatic metastasis, Immunotherapy

## Abstract

**Background:**

Chitinase-like proteins (CLPs) play a key role in immunosuppression under inflammatory conditions such as cancer. CLPs are enzymatically inactive and become neutralized upon binding of their natural ligand chitin, potentially reducing CLP-driven immunosuppression. We investigated the efficacy of chitin treatment in the context of triple-negative breast cancer (TNBC) using complementary mouse models. We also evaluated the immunomodulatory influence of chitin on immune checkpoint blockade (ICB) and compared its efficacy as general CLP blocker with blockade of a single CLP, i.e. chitinase 3-like 1 (CHI3L1).

**Methods:**

Female BALB/c mice were intraductally injected with luciferase-expressing 4T1 or 66cl4 cells and systemically treated with chitin in combination with or without anti-programmed death (PD)-1 ICB. For single CLP blockade, tumor-bearing mice were treated with anti-CHI3L1 antibodies. Metastatic progression was monitored through bioluminescence imaging. Immune cell changes in primary tumors and lymphoid organs (i.e. axillary lymph nodes and spleen) were investigated through flow cytometry, immunohistochemistry, cytokine profiling and RNA-sequencing. CHI3L1-stimulated RAW264.7 macrophages were subjected to 2D lymphatic endothelial cell adhesion and 3D lymphatic integration in vitro assays for studying macrophage-mediated lymphatic remodeling.

**Results:**

Chitin significantly reduced primary tumor progression in the 4T1-based model by decreasing the high production of CLPs that originate from tumor-associated neutrophils (TANs) and Stat3 signaling, prominently affecting the CHI3L1 and CHI3L3 primary tumor levels. It reduced immunosuppressive cell types and increased anti-tumorigenic T-cells in primary tumors as well as axillary lymph nodes. Chitin also significantly reduced CHI3L3 primary tumor levels and immunosuppression in the 66cl4-based model. Compared to anti-CHI3L1, chitin enhanced primary tumor growth reduction and anti-tumorigenicity. Both treatments equally inhibited lymphatic adhesion and integration of macrophages, thereby hampering lymphatic tumor cell spreading. Upon ICB combination therapy, chitin alleviated anti-PD-1 resistance in both TNBC models, providing a significant add-on reduction in primary tumor and lung metastatic growth compared to chitin monotherapy. These add-on effects occurred through additional increase in CD8α^+^ T-cell infiltration and activation in primary tumor and lymphoid organs.

**Conclusions:**

Chitin, as a general CLP blocker, reduces CLP production, enhances anti-tumor immunity as well as ICB responses, supporting its potential clinical relevance in immunosuppressed TNBC patients.

**Supplementary Information:**

The online version contains supplementary material available at 10.1186/s13058-024-01815-8.

## Background

Triple-negative breast cancer (TNBC) patients remain highly dependent on chemotherapy despite poor chances for survival and severe side effects [1]. Although immunotherapy, especially immune checkpoint blockade (ICB) such as anti-programmed death (PD)-1, has provided additional benefit for the metastatic TNBC setting, only 10–20% of patients eventually respond [2, 3]. The immunosuppressive tumor microenvironment (TME) in TNBC is an important contributor to this (immuno)therapeutic failure with tumor-associated macrophages (TAMs), neutrophils (TANs), myeloid-derived suppressor cells (MDSCs) and regulatory T-cells (T-regs) as the main attributors [4, 5]. Reducing the number and/or phenotype of these undesired cell types may therefore enhance therapeutic response in TNBC.

Chitinase-like proteins (CLPs) have originally been identified as immunosuppressive stimulators for wound healing, being highly expressed following resolution of pathogen infection and tissue damage [6, 7]. Subsequently, production of CLPs was also observed in non-infectious diseases characterized by inflammation such as cancer [6]. Chitinase 3-like 1 (CHI3L1), one of the main CLP family members, has been reported in a 4T1-based mouse model for TNBC [8] and in human TNBC patients [9, 10] to establish a wound healing response that contributes to the immunosuppressive TME [11]. More specifically, CHI3L1 drives immunosuppressive TAMs in the TME [12–14], but also contributes to the inhibition of anti-tumor (type 1) T-cell responses [15] and natural killer (NK) cell activity [16]. Similarly to CHI3L1, CHI3L3—a mouse CLP member with no human orthologue—was identified as a common marker and driver for immunosuppressive M2 TAM polarization in the TME [17, 18]. Collectively, given its evolutionary function in wound healing and associated immunosuppression, blocking CLPs may ameliorate TNBC-associated immunosuppression.

We here evaluated the influence of CLP blockade on disease progression and anti-PD-1 ICB in highly metastatic 4T1- and 66cl4-based intraductal models for TNBC. Since CLPs have a conserved chitin-binding domain but—in contrast to chitinases—no enzymatic activity [19], chitin can be used as a general CLP blocker in mouse BC models as shown more than a decade ago [20, 21]. Collectively, our data demonstrate that chitin monotherapy reduces primary tumor progression by attenuating the immunosuppressive TME in both complementary intraductal models that are characterized by a heterogenous CLP production. Moreover, as a general CLP blocker, chitin evokes enhanced anti-tumor immunity and concomitant tumor growth reduction compared to blockade of CHI3L1 alone, both treatments equally affecting lymphatic metastasis by hampering lymphatic vessel integration of macrophages. In combination with anti-PD-1, chitin treatment alleviates ICB resistance and further stimulates primary tumor as well as lymphoid anti-tumor immunity, additionally reducing primary tumor growth and lung metastasis.

## Methods

### 4T1-luc and 66cl4-luc cell culture

Firefly luciferase-expressing 4T1 (4T1-luc) mammary tumor cells which resemble metastatic human TNBC were provided as a kind gift from Prof. Clare Isacke (Breakthrough Breast Cancer Research Centre, London, UK). The 4T1-luc cells were cultured as previously described [22]. Firefly luciferase-expressing 66cl4 (66cl4-luc) cells were kindly provided by Prof. Traci R. Lyons (University of Colorado Anschutz Medical Campus, Denver, CO, USA). The 66cl4-luc cells were cultured using Dulbecco’s Modified Eagle’s Medium (DMEM, Thermo Fisher Scientific, Waltham, MA, USA) supplemented with 10% heat-inactivated fetal bovine serum (FBS, Thermo Fisher Scientific), 100U/ml penicillin, 100 µg/ml streptomycin (both from Sigma-Aldrich, Overijse, Belgium), 1% Gibco™ MEM Non-Essential Amino Acids Solution (Thermo Fisher Scientific) and 200 µg/ml hygromycin B (Thermo Fisher Scientific) at 37 °C and 5% CO2. Both cell lines were routinely checked for mycoplasma and other bacterial contamination using a PlasmoTest^TM^ mycoplasma detection kit (Invivogen, San Diego, USA). Harvesting of confluent cells was performed through incubation with 0.25% trypsin-ethylenediaminetetraacetic acid (EDTA) (Sigma-Aldrich) for 5 min and centrifugation at 400 × g for 5 min for subsequent counting with a Bürker chamber.

### Intraductal inoculations

Animal experiment protocols were approved by the Ethical Committee (EC) of the Faculty of Veterinary Medicine, Ghent University (EC 2021–074) and performed according to Good Scientific Practice principles. Sample sizes were a priori calculated based on preliminary data from a pilot study (primary tumor volume as primary outcome measure), identifying that 7 mice per group for the 4T1- and 11 mice per group for the 66cl4-based model are sufficient to detect statistically significant differences.

Female BALB/c mice of 8 weeks (w) old were mated with male BALB/c mice of similar age and gave birth to pups within 3 w. The pups were weaned 12–14 days (d) later, after which the individually caged, lactating mother mice were intraductally inoculated in the third mammary gland pair with 5 × 10^4^ 4T1-luc or 1–2 × 10^5^ 66cl4-luc cells suspended in a cold mixture of PBS and Matrigel® (1:10; Corning, Bedford, MA, USA). The intraductal inoculations were conducted using a 32G pediatric needle under isoflurane inhalation anesthesia and buprenorphine analgesia as described previously [22].

### Chitin preparation and systemic treatment

A chitin particle-containing mixture was prepared as described previously [23]. In short, chitin powder from shrimp shells (Sigma-Aldrich) suspended in PBS at a concentration of 10 mg/ml was sonicated to reduce the chitin particle size using a Branson Digital Sonifier 450 (VWR, Branson Ultrasonics). Subsequent filtration through a 10 µm strainer (pluriSelect, Leipzig, Germany) and pelleting through centrifugation at 250 × g for 10 min allowed further size selection. The composition of the chitin suspension was investigated through flow cytometry (CytoFLEX, Analis, Ghent, Belgium) with 1 and 9.9 µm-sized Fluoro-Max™ Green Fluorescent Polymer Microspheres (Thermo Fisher Scientific), allowing to verify chitin particle sizes between 1 and 9.9 µm (Additional file 1: Figure S1A). Based on a previous study, the degree of polymerization and acetylation of the chitin suspension was also characterized and verified using Matrix-Assisted Laser Desorption/Ionization (MALDI) Time-Of-Flight (TOF) Mass Spectrometry (MS) (Additional file 1: Figure S1B) [24]. Individually-caged mice were assigned to a group and provided with treatment, single-blinded (up to the end of the study) in a climate-controlled animal facility. Systemic administration of 1 mg chitin/mouse was established through intraperitoneal (i.p.) injection of 100 µl of the final chitin mixture. Anti-mPD-1-mIgG1e3 InvivoFit™ (Invivogen) derived from clone RMP1-14 and anti-β-Gal-mIgG1e3 InvivoFit™ (Invivogen) clone T9C6 were used for anti-PD-1 ICB and isotype control, respectively, through i.p. injection at 25 µg or 200 µg/mouse as previously described [22]. Anti-CHI3L1 clone 4E3-F2 (M3; Absolute Antibody, Oxford, UK) and anti-Fluorescein clone 4–4-20 (Absolute Antibody) were used for CHI3L1 blockade and isotype control, respectively, through i.p. injection at 200 µg/mouse.

### Monitoring of disease progression

4T1-luc and 66cl4-luc primary tumor progression was weekly monitored through caliper-based primary tumor volume measurements and imaging of bioluminescent signals in the primary tumor area. For the in vivo imaging, tumor-bearing mice were i.p. injected with 200 µl D-luciferin (20 mg/ml PBS; PerkinElmer, Zaventem, Belgium) and caudally placed in the IVIS Lumina III system (PerkinElmer) under isoflurane inhalation anesthesia as described previously [22]. For the ex vivo imaging of metastases, tumor-bearing mice were terminally sedated and sacrificed through cervical dislocation, after which axillary lymph nodes, lungs and spleen were quickly isolated and bioluminescent signals were captured with the IVIS system. Imaging analysis was performed with the living image software 4.7.2.

### ELISA and cytokine profiling

Whole tissue/cellular lysate preparation and subsequent protein concentration assessment were performed as previously described [25]. CHI3L1 and CHI3L3 levels were measured in the prepared lysates using the Mouse Chitinase 3-like 1/YKL-40 Quantikine enzyme-linked immunosorbent assay (ELISA) Kit (Bio-techne, Minneapolis, MN, USA) and the Mouse YM1/Chitinase 3-like 3 DuoSet ELISA (Bio-techne), respectively, according to the manufacturer’s instructions. A panel of 9 cytokines (granulocyte-colony stimulating factor (G-CSF), interferon (IFN)-γ, interleukin (IL)-1β, IL-4, IL-6, IL-10, monocyte chemoattractant protein (MCP)-1, macrophage inflammatory protein (MIP)-2 and tumor necrosis factor (TNF)-α) was investigated in 50 µg of the prepared lysates using the Luminex Multiplex Assay (ProcartaPlex from Thermo Fisher Scientific), and transforming growth factor (TGF)-β1 levels were measured using ELISA (Thermo Fisher Scientific) according to the manufacturer’s instructions.

### Western blotting

Primary tumor lysates were loaded at 25 µg per lane on 12% Mini-PROTEAN TGX Precast Protein Gels (Bio-Rad, CA, USA). Proteins in all gels were electrophoretically separated and transferred onto a 0.45 µm nitrocellulose membrane (Bio-Rad). All further steps were performed on a shaking platform. Membranes were blocked for 1 h at room temperature (RT) with freshly prepared blocking buffer (tris-buffered saline (TBS) with 0.1% Tween-20 and 5% non-fat dry milk). After blocking, membranes were incubated with primary antibody (diluted in blocking buffer) overnight (ON) at 4 °C. Following primary antibodies were used: anti-CHI3L3 (1:1000, clone #281926, Bio-techne), anti-CHI3L3 + CHI3L4 (1:10,000, clone EPR15263, Abcam, Cambridge, UK), anti-CHI3L1 (1:1000, clone EPR23891-162, Abcam), anti-SI-CLP (1:1000, polyclonal, Thermo Fisher Scientific), anti-ovidcutin (1:100, clone H-8, HRP-conjugated, Santa Cruz Biotechnology, Dallas, TX, USA) and anti-glyceraldehyde 3-phosphate dehydrogenase (GAPDH; 1:5000, clone EPR16891, HRP-conjugated, Abcam). The next day, prior to secondary antibody incubation for 1 h at RT, membranes were washed three times for 5 min with blocking buffer. Following secondary antibodies were used: goat anti-rat IgG HRP-conjugated antibody (1:1000, Bio-techne), donkey anti-rabbit IgG HRP-conjugated polyclonal antibody (1:10,000, Thermo Fisher Scientific). Immediately after incubations with secondary antibodies, membranes received three additional washes for 5 min each with blocking buffer and one wash step with deionized water for 15 min. In order to reprobe blots with subsequent antibodies, membranes were incubated with TBS containing 0.1% sodium azide ON and with stripping buffer (Restore PLUS Western Blot Stripping Buffer, Thermo Fisher Scientific) the following day for 20 min at 37 °C on a shaking platform. Full, uncropped western blot images are provided in Additional files 18 and 19: Fig. S16 and S17. CHI3L1 antibody specificity was also validated on a blot loaded with recombinant mouse (rm)CHI3L1 protein, verifying a signal at 43 kDa as reported by the manufacturer and other groups [26, 27] (Additional file 18: Fig. S16). Pageruler Prestained colorimetric Protein Ladder (Thermo Fisher Scientific) and chemiluminescent MagicMark (Invitrogen) were used as reference molecular weight markers on the membranes. Protein bands were detected using a ChemiDoc MP Imaging System and signal quantification was performed with ImageJ.

### Immunohistochemistry

Primary tumor and lung tissue was fixed in 3.5% phosphate-buffered formaldehyde for 24 h and subsequently embedded in paraffin. For histology, 5 μm sections were deparaffinized and rehydrated, followed by staining with hematoxylin and eosin (H&E). For immunohistochemical stainings, 2–3 µm sections were deparaffinized and rehydrated, followed by pressurized antigen retrieval for 30 min at 95 °C using either a 10 mM citrate buffer (VWR International, Leuven, Belgium) at pH 6 or a 10 mM Tris-1 mM EDTA buffer (Sigma-Aldrich) at pH 9, both supplemented with 0.05% Tween 20 (Sigma-Aldrich). Endogenous peroxidase and non-specific protein binding were subsequently blocked for 10 min using respectively 3% H_2_O_2_ (Sigma-Aldrich) in methanol and a serum-free protein blocking buffer (Dako, Heverlee, Belgium), followed by a 1 h primary antibody staining step. The primary antibodies were therefore diluted in antibody diluent (Dako). Description of the used antibodies, clones, dilutions, supplier and used antigen retrieval buffer are summarized in Table S1 (Additional file 2). Depending on the used primary antibody, either Rat-on-Mouse HRP-Polymer (Biocare Medical, CA, USA) or Dako EnVision + Rabbit (Dako) was used as secondary antibodies for 30 min. In between every step, the slides were kept in a humidified box and washed 3 × 5 min using TBS (Thermo fisher Scientific). To provide proper detection of positive staining, the tissue slides were treated for 10 min with a 3,3’-diaminobenzidine (DAB)-containing buffer (Dako) and submerged for 5 min in Harris Hematoxylin (Carl Roth, Karlsruhe, Germany) and 1 min in 0.1 N HCl (Sigma-Aldrich) for counterstaining. Dual stainings on primary tumor sections were performed as previously described [28], using DAB-containing buffer and the Vina Green Chromogen Kit (Biocare Medical) to detect positive staining of both markers. For subsequent microscopic analysis, the tissue slides were dehydrated and mounted with Entellan (Sigma-Aldrich) and ImageJ was applied to quantify positive staining (color deconvolution and automatic counting of % area).

### Flow cytometric immunophenotyping and cell separation

Primary tumors, axillary lymph nodes and spleens were isolated from untreated and treated mice upon sacrification. Primary tumors were digested into single cell suspensions using a commercially available tumor dissociation kit and gentleMACS Dissociator with accompanying C tubes (Miltenyi Biotec, Leiden, The Netherlands) as previously described [22]. Manual processing of axillary lymph nodes and spleens into single cell suspensions was also performed as previously described [22].

The resulting number of cells was determined by adding 20 µl of Trucount beads (BD Biosciences, Erembodegem, Belgium) to 180 µl of diluted cell suspension in a well of a 96 well plate for analysis on a CytoFLEX flow cytometer (Analis). Absolute numbers of cells were then calculated based on Trucount technology, taking the dilution factor into account. Cellular viability was also evaluated by staining with a viobility 488/520 fixable dye (Miltenyi Biotec) according to the manufacturer’s instructions. Remaining cell suspensions were pelleted through centrifugation and plated in a 96 well plate to obtain a final number of 2 × 10^5^–1 × 10^6^ cells in each well. Cellular stainings for identification of immune cell subtypes, flowcytometric analysis and data processing were performed as previously described [22]. Intracellular cytoplasmic stainings for granzyme B and IFN-γ were performed using an Inside Stain Kit (Miltenyi Biotec), nuclear stainings for Ki67 and FoxP3 were performed using a FoxP3 staining kit (Miltenyi Biotec) according to the manufacturer’s instructions. The used fluorophore-conjugated antibodies and concentrations for cellular stainings are summarized in Table S2 (Additional file 3). In short, following immune cell populations were selected to be analysed: CD45^+^ CD11b^+^ CD14^+^ Ly6C^int^ Ly6G^+^ polymorphonuclear (PMN)-MDSCs, CD45^+^ CD11b^+^ CD14^+^ Ly6C^hi^ Ly6G^−^ monocytic (M)-MDSCs, CD45^+^ CD11b^+^ CD14^−^ Ly6C^int^ Ly6G^+^ neutrophils/TANs, CD45^+^ CD11b^+^ CD14^+^ F4/80^+^ macrophages/TAMs, and also more specific CD45^+^ CD11b^+^ CD14^+^ F4/80^+^ CD80^+^ MHCII^+^ M1 and CD45^+^ CD11b^+^ CD14^+^ F4/80^+^ CD206^+^ M2 macrophage/TAM subtypes, CD45^+^ CD11b^+^ CD11c^+^ dendritic cells (DCs), CD4^+^ and CD8α^+^ T-cells (CD45^+^ CD3ε^+^ CD4^+^ CD8α^−^ and CD45^+^ CD3ε^+^ CD4^−^ CD8α^+^, respectively), CD45^+^ CD3ε^−^ NKp46^−^ CD19^+^ B220^+^ B-cells, CD45^+^ CD3ε^−^ NKp46^+^ NK and CD45^+^ CD3ε^+^ NKp46^+^ NK-T cells.

Besides flow cytometric immunophenotyping, single cell suspensions of 4T1 primary tumors were also subjected to cellular separation for downstream protein analysis. More specifically, CD45^−^ and CD45^+^ cells were separated on a MACSQuant Tyto Cell Sorter (Miltenyi Biotec). Separation of Ly6G^+^ and Ly6G^−^ cells relied on magnetic bead isolation using anti-Ly6G MicroBeads UltraPure (Miltenyi Biotec). In short, up to 2 × 10^8^ Ly6G^+^ cells in the single cell suspension of primary tumors were magnetically labelled with MicroBeads, after which the cell suspension was loaded on a MACS MS Column and placed in a MACS Separator. According to the manufacturer’s instructions, labeled Ly6G^+^ cells are retained whereas Ly6G^−^ cells flow through with subsequent washing steps. Purity of both the Ly6G^+^ and Ly6G^−^ fraction was verified through labeling with anti-Ly6G-APC antibody and flow cytometric analysis.

### RNA-sequencing

RNA was extracted from untreated, anti-PD-1-, chitin- and chitin + anti-PD-1-treated 4T1 and 66cl4 primary tumors using the Rneasy Mini Kit (QiAgen, Valencia, CA, USA) according to manufacturer’s instructions. The extracted RNA was stored at -80 °C until downstream transcriptomic analysis using Illumina’s TruSeq chemistry in collaboration with Azenta Life Sciences (Leipzig, Germany). Therefore, 250 ng of RNA was converted into sequencing libraries and sequencing (2 × 150 bp) was performed on a NovaSeq instrument (Illumina Inc., San Diego, CA, USA), aiming for 10 M reads per sample. Then, raw reads were mapped to the murine reference genome (version mm10) using HISAT2 and resulting SAM files were converted to sorted BAM files using *samtools*. The number of mapped reads per genomic location was counted using the summariseOverlaps-function from the BioC-package *GenomicAlignments* in the intersection non-empty mode, taking strand information and unpaired reads into account. Finally, all genes with raw expression counts above 10 in at least 10% of the cases (N = 14,266) were filtered in for further analysis. Raw count data were normalized using variance stabilizing normalization (DESeq2) and the normalized expression data of selected genes in the different treatment groups were presented in a heatmap using the online Heatmapper tool [29]. One outlier sample (primary tumor sample 3 from the untreated 4T1-based model), was removed from the analyses due to RNA degradation.

### 2D LEC adhesion and 3D lymphatic integration assay

RAW264.7 macrophages’ ability to adhere to human-derived lymphatic endothelial cells (HDLECs) or integrate into lymphatic vessel structures following stimulation with rmCHI3L1 was evaluated using a 2D adhesion and 3D integration assay, respectively. Therefore, RAW264.7 macrophages, cultured as previously described [14], were pretreated ON with 5 μg/ml rmCHI3L1 (Bio-techne), in addition of 0.6 μg/ml anti-PDPN (clone pMAB, Thermo Fisher Scientific) or 0.6 μg/ml rat IgG control (BioXCell, Lebanon, USA), 2 µg/ml IL13Rα2 (polyclonal, Bio-techne) or 2 µg/ml goat IgG control (Bio-techne). The treated RAW264.7 macrophages were fluorescently stained the following day with CellTracker (Thermo Fisher Scientific) and seeded on a HDLEC monolayer for the 2D adhesion assay or together with HDLECs on top of a 4 mg/ml Matrigel pad for the 3D integration assay as previously described [28]. Images were taken after 4 h for the 2D adhesion assay, and after 7 h for the 3D integration assay.

### Statistical analysis

Statistics with data normalization through log10 normalization were performed with Prism (Graphpad). Differences between multiple treatment groups were calculated by unpaired Student’s t-tests or Analysis of Variance (ANOVA) tests followed by Newman-Keuls post-hoc testing, with P-values < 0.05 identified as statistically significant.

## Results

### Chitin enhances anti-PD-1 ICB efficacy in both the 4T1- and 66cl4-based TNBC model

**Fig. 1:**
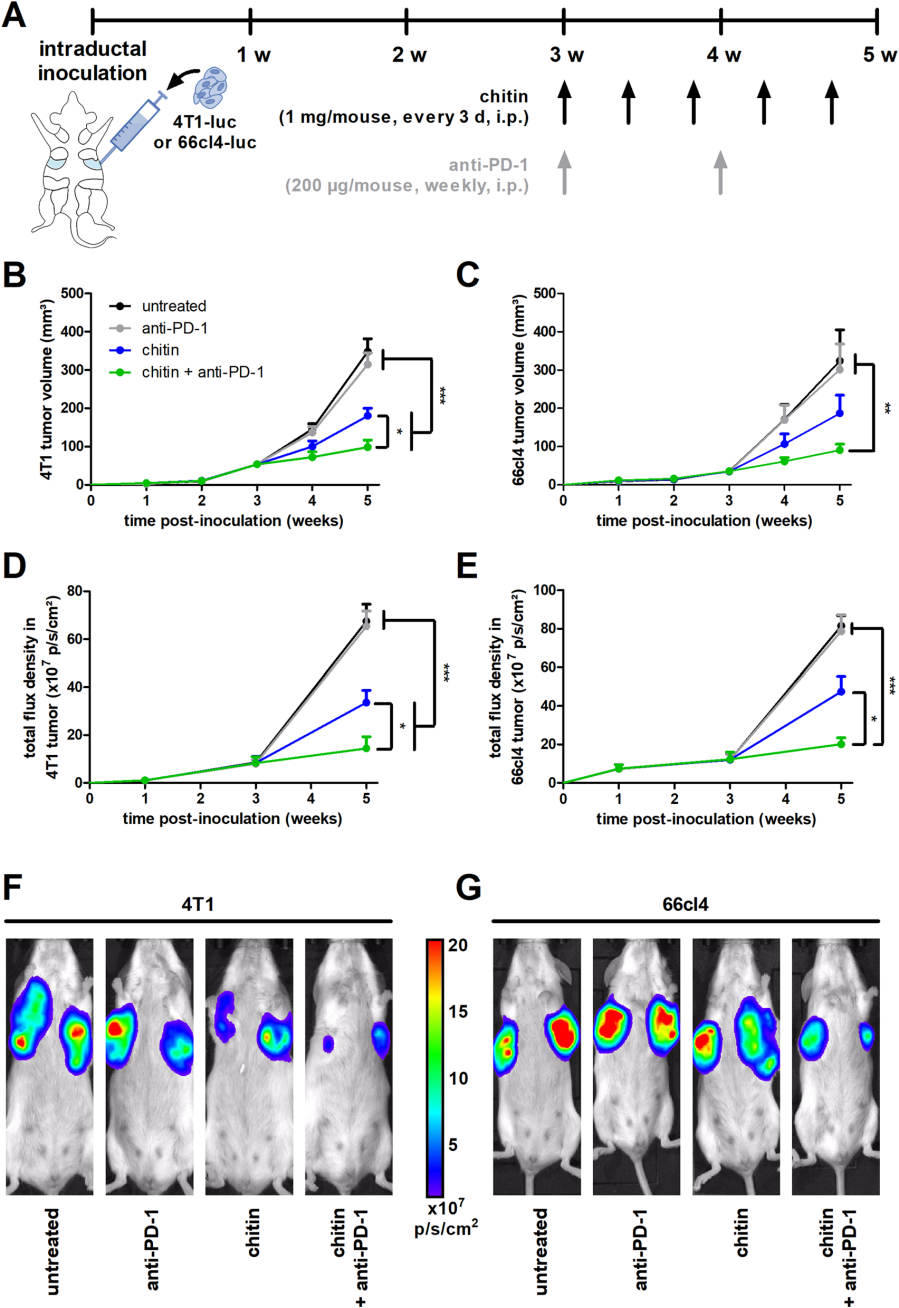
Chitin reduces tumor progression and enhances anti-PD-1 efficacy in a 4T1- and 66cl4-based intraductal model. **A** Experimental timeline with chitin and anti-PD-1 treatment schedules. **B,C** Weekly measurements of primary tumor volumes across the 5-w study period in the untreated and treated 4T1- (**B**) and 66cl4-based model (**C**) (n = 14 for all groups at all time points in the 4T1-based model; n = 22 for all groups at all time points in the 66cl4-based model). **D,E** In vivo imaging of primary tumor bioluminescent signals (total flux density in p/s/cm^2^) in the untreated and treated 4T1- (**D**) and 66cl4-based model (**E**) (n = 14 for all groups at all time points in the 4T1-based model; n = 22 for all groups at all time points in the 66cl4-based model). **F,G** Representative images of primary tumor bioluminescence in the untreated and treated 4T1- (**F**) and 66cl4-based model (**G**) at 5 w p.i. Data are presented as the means ± standard error of the mean (SEM). *P < 0.05, **P < 0.01, ***P < 0.001

Following intraductal injection with 4T1-luc or 66cl4-luc cells, mammary tumors were allowed to develop for 3 w, fully transition to the invasive carcinoma stage and commence metastasis. As these events indicate for systemic therapy in the clinic, treatment with 1 mg chitin (every 3 d) as blocker of CLPs either combined with or without 200 µg anti-PD-1 ICB (weekly) was initialized at that time point through i.p. injections and continued for 2 w until 5 w post-inoculation (p.i.) (Fig. 1A). Based on body weight and temperature, chitin either as monotherapy or in combination with anti-PD-1 was well tolerated in the 4T1- and 66cl4-based model (Additional file 4: Fig. S2A-D).

In line with the ICB-resistant nature of the 4T1- and 66cl4-based model, anti-PD-1 monotherapy did not reduce disease progression by 5 w p.i. compared to untreated tumor-bearing mice, based on tumor volume measurements (Fig. 1B,C) and concomitant in vivo imaging (Fig. 1D–G). In marked contrast, chitin monotherapy significantly reduced 4T1 primary tumor growth (Fig. 1B,D) and also showed a growth reductive effect in 66cl4 primary tumors (Fig. 1C,E), albeit not statistically significant, compared to untreated and anti-PD-1-treated tumor-bearing mice. Combined with anti-PD-1, chitin further and significantly reduced primary tumor growth compared to untreated, anti-PD-1- and chitin-treated tumor-bearing mice in both the 4T1- and 66cl4-based model (Fig. 1B–G). This add-on treatment effect upon combination of anti-PD-1 with chitin was anti-PD-1 dose-dependent, as a low (25 µg) anti-PD-1 dose in combination with chitin did not provide a significant additional tumor growth reduction compared to chitin monotherapy in the 4T1-based model based on primary tumor volume (Additional file 5: Fig. S3A) and in vivo imaging (Additional file 5: Fig. S3B,C).

**Fig. 2:**
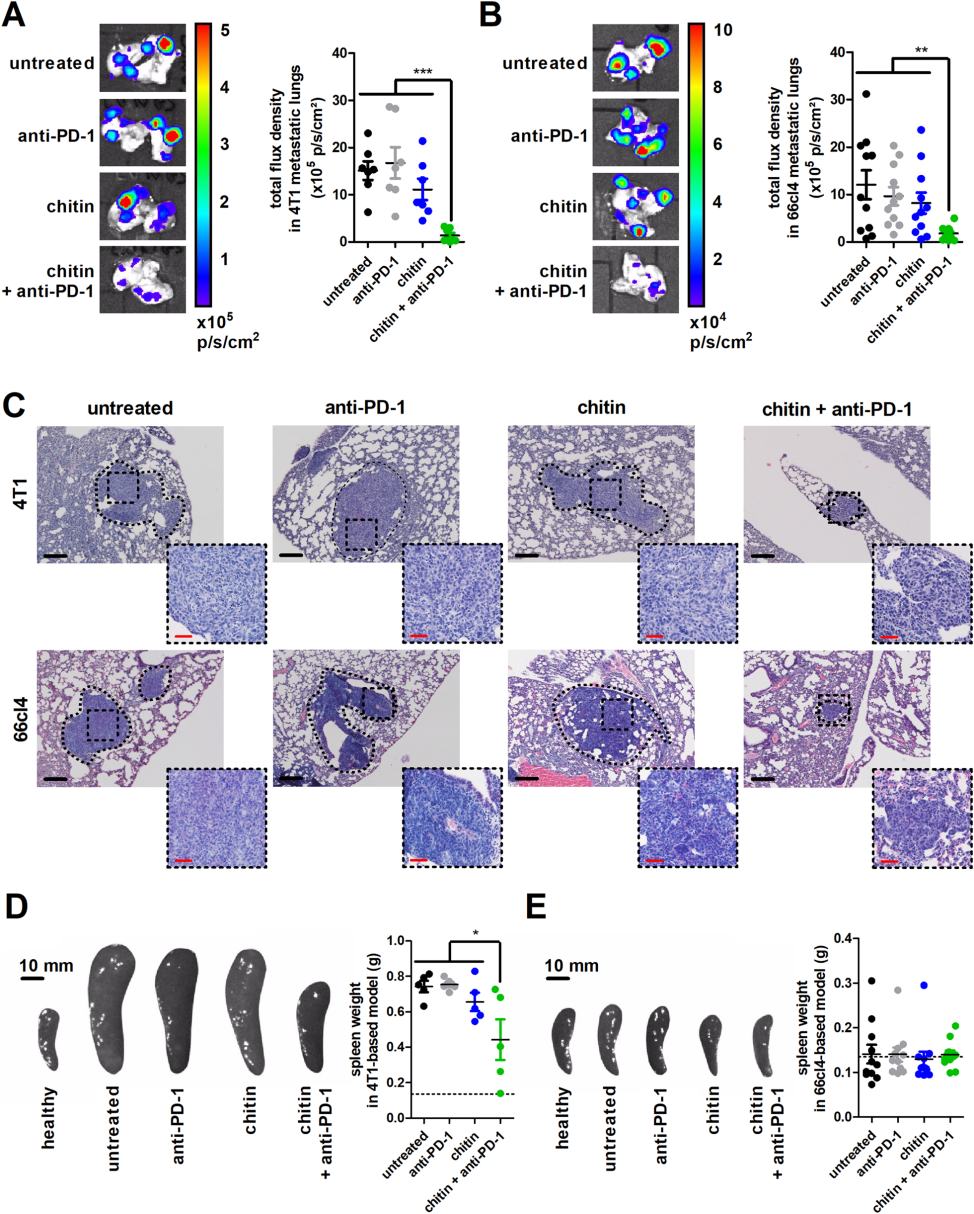
Chitin and anti-PD-1 combination has a reductive effect on metastatic progression in the 4T1- and 66cl4-based intraductal model. **A,B** Representative images and quantification of bioluminescent signals (total flux density in p/s/cm^2^) in lungs from the untreated and treated 4T1- (**A**) and 66cl4-based model (**B**) at 5 w p.i. (n = 7 for all groups in the 4T1-based model; n = 11 for all groups in the 66cl4-based model). **C** H&E histology of lung metastases from the untreated and treated 4T1- and 66cl4-based model at 5 w p.i. Dashed inserts highlight H&E-stained metastases at a larger magnification. Black scale bars = 200 µm, red scale bars = 50 μm. **D,E** Representative images and weight measurements of the spleen from the untreated and treated 4T1-(**D**) and 66cl4-based model (**E**) at 5 w p.i. (n = 5 for all groups in the 4T1-based model; n = 11 for all groups in the 66cl4-based model). An image of a healthy spleen is shown for comparison and the dotted line in the graph highlights the mean spleen weight of 4 healthy BALB/c mice. Data are presented as the means ± SEM. *P < 0.05, **P < 0.01, ***P < 0.001

To evaluate systemic disease following 2-w treatment with chitin either in combination with or without 200 µg anti-PD-1, lungs were ex vivo imaged for metastatic 4T1- and 66cl4-derived bioluminescence. Only the combination of chitin with anti-PD-1 significantly reduced lung bioluminescent signals in both TNBC models (Fig. 2A,B) and these reduced metastatic signals were confirmed by H&E histology (Fig. 2C). A low (25 µg) anti-PD-1 dose in combination with chitin again did not provide a significant metastatic reduction in the lungs of the 4T1-based model (Additional file 5: Fig. S3D,E), confirming the need for a high (200 µg) anti-PD-1 dose to obtain treatment efficacy.

Spleen size and weight in the 4T1-based model have been associated with systemic disease progression by our group and others [8, 30], and were both significantly increased in untreated, anti-PD-1- and chitin-treated 4T1 tumor-bearing mice. Chitin treatment in combination with anti-PD-1 significantly reduced the spleen size and weight in the 4T1-based model, albeit not to the level of a healthy spleen (Fig. 2D). The synergistic reduction in splenomegaly was only obtained with a high (200 µg) anti-PD-1 dose, a low (25 µg) anti-PD-1 dose did not provide these reductions upon combination with chitin (Additional file 5: Fig. S3F). In marked contrast to 4T1 tumor-bearing mice, the size and weight of spleens from 66cl4 tumor-bearing mice did not differ from those of a healthy spleen and were not affected by either treatment (Fig. 2E).

### TAN-derived CLP production is higher in 4T1 compared to 66cl4 primary tumors and is reduced by chitin treatment

**Fig. 3:**
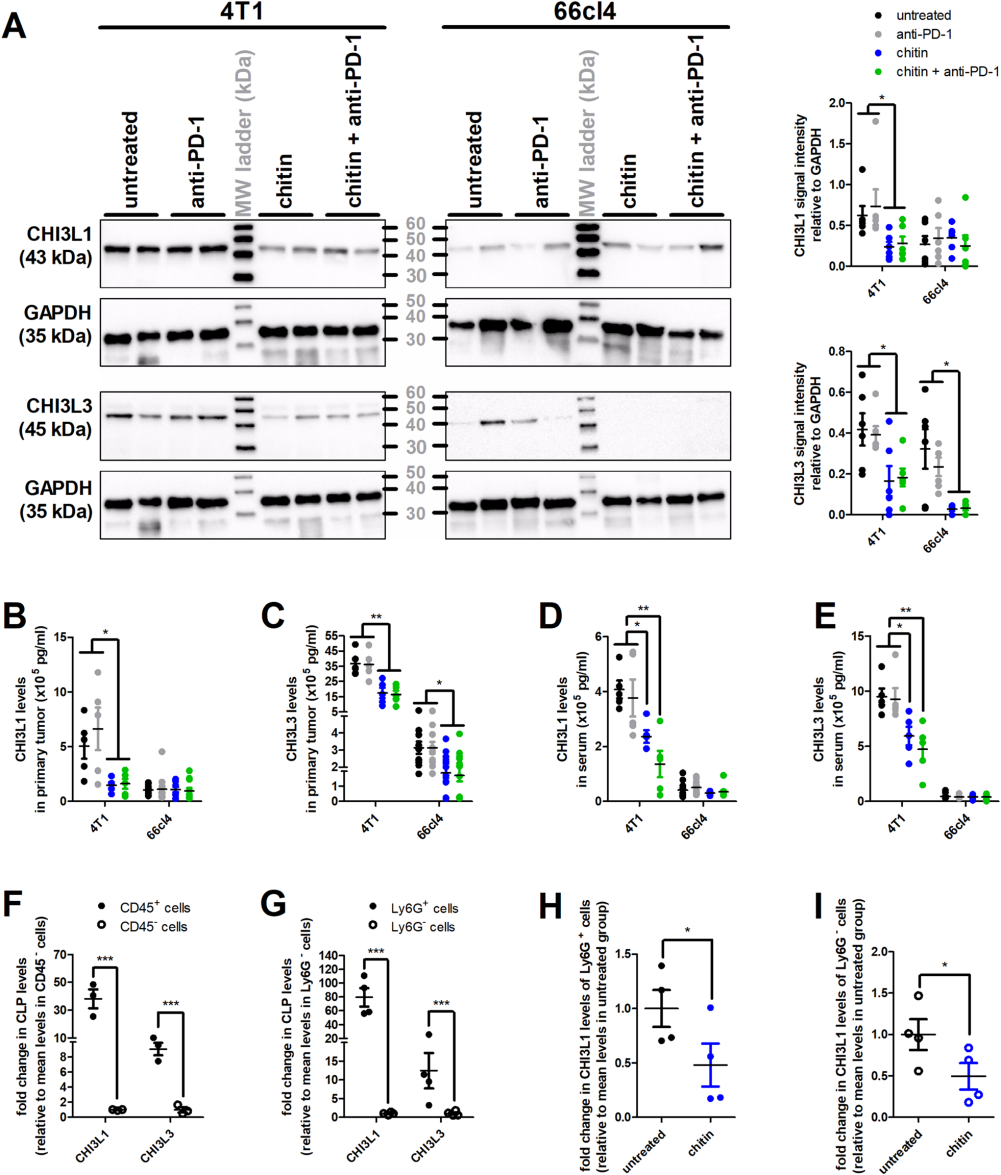
Chitin reduces high CHI3L1 and CHI3L3 production in the 4T1-based and even low CHI3L3 production in the 66cl4-based intraductal model. **A** Representative western blot images for CHI3L1, CHI3L3 and GAPDH loading control in primary tumor lysates from untreated, anti-PD-1-, chitin- and chitin + anti-PD-1-treated 4T1 and 66cl4 tumor-bearing mice at 5 w p.i. with quantification of CHI3L1 and CHI3L3 signal intensity relative to GAPDH (n = 6 for all groups; 3 western blots with 2 samples from each group per blot). **B** CHI3L1 primary tumor levels in the untreated and treated 4T1- (n = 5 for all groups) and 66cl4-based model (n = 11 for all groups) at 5 w p.i. **C** CHI3L3 primary tumor levels in the untreated and treated 4T1- (n = 5 for all groups) and 66cl4-based model (n = 11 for all groups) at 5 w p.i. **D** CHI3L1 serum levels in the untreated and treated 4T1- (n = 5 for all groups) and 66cl4-based model (n = 11 for all groups) at 5 w p.i. **E** CHI3L3 serum levels in the untreated and treated 4T1- (n = 5 for all groups) and 66cl4-based model (n = 11 for all groups) at 5 w p.i. **F** Fold change in CHI3L1 and CHI3L3 levels between separated CD45^+^ and CD45^−^ cells from untreated 4T1 primary tumors at 5 w p.i. (n = 3 for all groups). **G** Fold change in CHI3L1 and CHI3L3 levels between separated Ly6G^+^ and Ly6G^−^ cells from untreated 4T1 primary tumors at 3 w p.i. (n = 4 for all groups). **H** Fold change in CHI3L1 levels between Ly6G^+^ cells derived from untreated versus chitin-treated 4T1 primary tumors at 3 w p.i. (n = 4 for all groups). **I** Fold change in CHI3L1 levels between Ly6G^−^ cells derived from untreated versus chitin-treated 4T1 primary tumors at 3 w p.i. (n = 4 for all groups). Data are presented as the means ± SEM. *P < 0.05, **P < 0.01, ***P < 0.001

Complementary immuno-assays on primary tumor tissue lysates identified local CHI3L1 and CHI3L3 production in both TNBC models, albeit higher in the 4T1 compared to the 66cl4 primary tumors (Fig. 3). Chitin monotherapy significantly reduced both CHI3L1 and CHI3L3 levels in 4T1 primary tumors, and CHI3L3 levels in 66cl4 primary tumors based on western blot analysis (Fig. 3A) as well as ELISA (Fig. 3B,C). Combining chitin with anti-PD-1 treatment did not further decrease these levels compared to chitin monotherapy in either model (Fig. 3A-C). Additional western blotting for related CLP family members identified local production of CHI3L4, again higher in 4T1 than in 66cl4 primary tumors (Additional file 6: Fig. S4). Stabilin-1 interacting chitinase-like protein (SI-CLP) and oviductin were not detected (data not shown). Chitin with or without anti-PD-1 also reduced CHI3L4 production in primary tumors of both models, albeit not statistically significantly (Additional file 6: Fig. S4C).

Systemically, CHI3L1 and CHI3L3 levels were also higher in the 4T1- compared to the 66cl4-based model (Fig. 3D,E). Chitin monotherapy significantly reduced serum levels of both CLPs in the 4T1-based model only (Fig. 3D,E). Chitin + anti-PD-1 combination treatment had an add-on effect in the 4T1-based model with a more significant reduction of CHI3L1 and CHI3L3 serum levels compared to untreated and anti-PD-1-treated tumor-bearing mice (Fig. 3D,E). Yet, serum CLP levels in chitin- compared to chitin + anti-PD-1-treated 4T1 tumor-bearing mice did not statistically significantly differ.

In order to explore the origin of these local and systemic reductions in CHI3L1 and CHI3L3 upon chitin treatment, their main cellular sources were subsequently investigated. CHI3L1 and CHI3L3 levels were highest in the CD45^+^ fraction of 4T1 primary tumors (Fig. 3F), identifying immune rather than tumor cells as major producers of both CLPs in the TME. Moreover, based on the staining location on primary tumor sections, CHI3L1 and CHI3L3 were almost exclusively present at cellular necrosis sites (Additional file 7: Fig. S5A,B), which were highly populated with Ly6G^+^ TANs, albeit with a much lower abundance in 66cl4 compared to 4T1 primary tumors (Additional file 7: Fig. S5C). Chitin with or without anti-PD-1 significantly reduced the CHI3L3 levels and the concomitant Ly6G staining in primary tumors of both TNBC models, and provided additional reduction of CHI3L1 levels in 4T1 primary tumors (Additional file 7: Fig. S5).

**Fig. 4:**
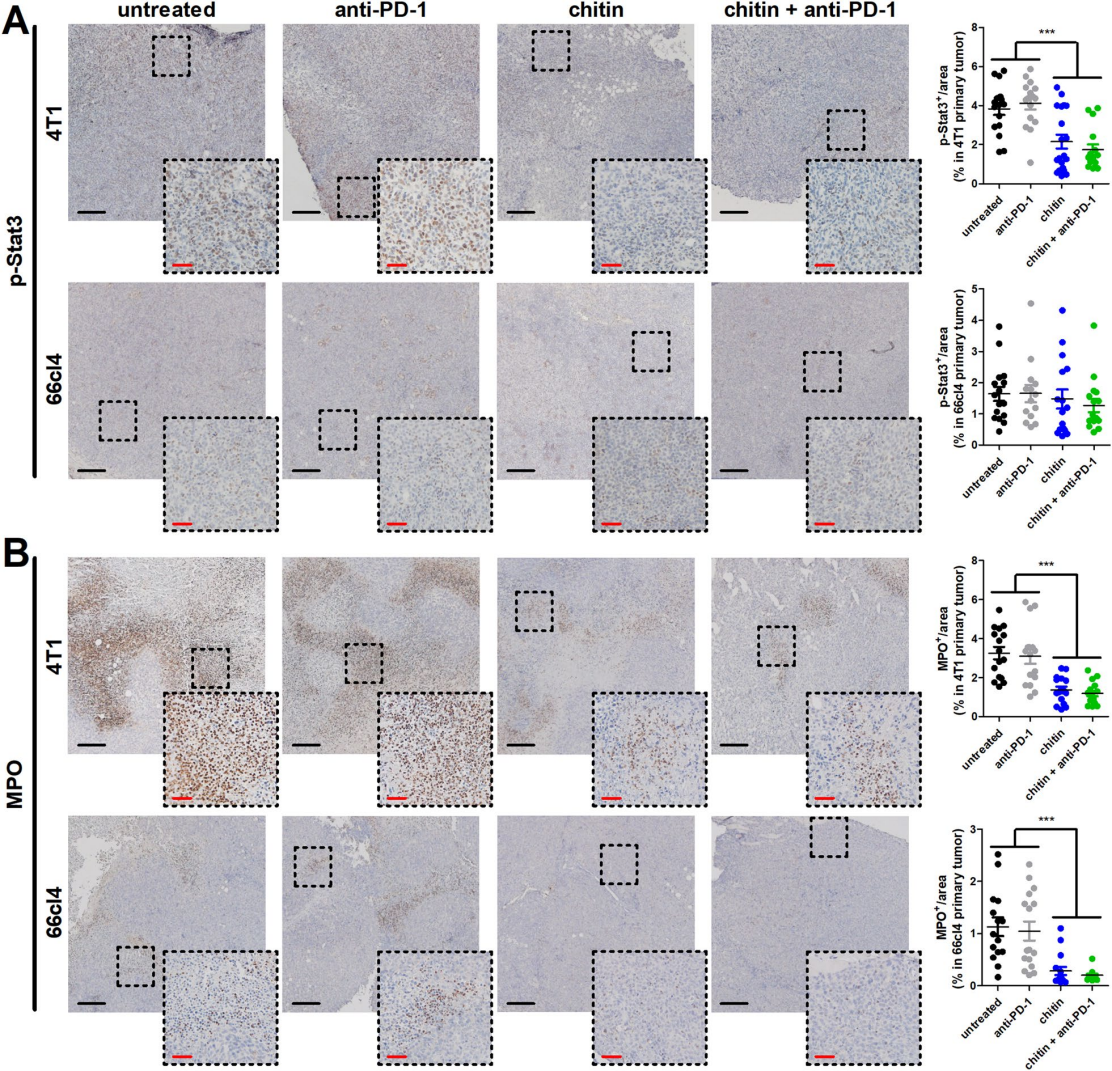
Chitin reduces p-Stat3 levels in 4T1 primary tumors and MPO positivity in both 4T1 and 66cl4 primary tumors. **A,B** Immunohistochemistry for p-Stat3 (**A**) and MPO (**B**) on primary tumor sections from untreated, anti-PD-1-, chitin- and chitin + anti-PD-1-treated 4T1 and 66cl4 tumor-bearing mice at 5 w p.i. (n = 16 for all groups; 4 slides with 4 images per slide). Dashed inserts highlight stained tissue at a larger magnification. Black scale bars = 200 µm, red scale bars = 50 μm. Data are presented as the means ± SEM. ***P < 0.001

In order to unequivocally identify TANs as main CLP producers, Ly6G^+^ and Ly6G^−^ fractions were also magnetically isolated from untreated compared to chitin-treated 4T1 primary tumors at 3 w p.i. Of note, the 3-w endpoint avoided tumor necrosis that can interfere with cellular separation, and chitin-treated primary tumors were subjected to a daily dose of 1 mg chitin to maximize the chitin treatment effect on downstream cellular targets. ELISA showed significantly increased CHI3L1 and CHI3L3 levels in the Ly6G^+^ compared to the Ly6G^−^ fraction from untreated primary tumors (Fig. 3G), and CHI3L1 levels significantly decreased in both the Ly6G^+^ (Fig. 3H) and even the low CHI3L1-expressing Ly6G^−^ primary tumor fraction upon chitin treatment (Fig. 3I). Binding and signaling of CHI3L1 has been linked to signal transducer and activator of transcription (Stat) 3 phosphorylation and subsequent production of CHI3L1 [31–33]. As a result, reduction of CHI3L1 levels in the primary tumor upon chitin treatment would be intricately linked to reduced Stat3 phosphorylation. Indeed, additional stainings on primary tumor sections showed that p-Stat3 levels were significantly reduced in the chitin- and chitin + anti-PD-1-treated compared to untreated and anti-PD-1-treated 4T1 primary tumors, but this reductive effect was not detected in the low CLP-producing 66cl4 primary tumors (Fig. 4A). Furthermore, the stainings also showed a higher p-Stat3 positivity in 4T1 compared to 66cl4 primary tumors, corroborating with a higher CLP production in the 4T1- compared to 66cl4-based model (Fig. 4A). Besides its impact on TAN-derived CLP production, chitin treatment also reduced pro-tumorigenic formation of neutrophil extracellular traps (NETs) based on significantly decreased myeloperoxidase (MPO) stainings in chitin-treated primary tumors compared to untreated and anti-PD-1-treated primary tumors from both the 4T1- and 66cl4-based model (Fig. 4B). Combining anti-PD-1 with chitin did not further reduce the MPO stainings compared to chitin monotherapy in primary tumors from either model (Fig. 4B).

### Chitin reduces immunosuppression and enhances ICB immunostimulation in 4T1 and 66cl4 primary tumors

In order to gain further insight into the immunological changes associated with chitin treatment either in combination with or without ICB, extensive flow cytometric immunophenotyping was performed on 4T1 and 66cl4 primary tumors at 5 w p.i. (Methods section and Additional files 8 and 9: Fig. S6 and S7, including detection of the myeloid subpopulations PMN-MDSCs, M-MDSCs, TANs, TAMs and DCs, and the lymphocytic subpopulations CD4^+^ and CD8α^+^ T-cells, B-cells, NK and NK-T cells).

**Fig. 5:**
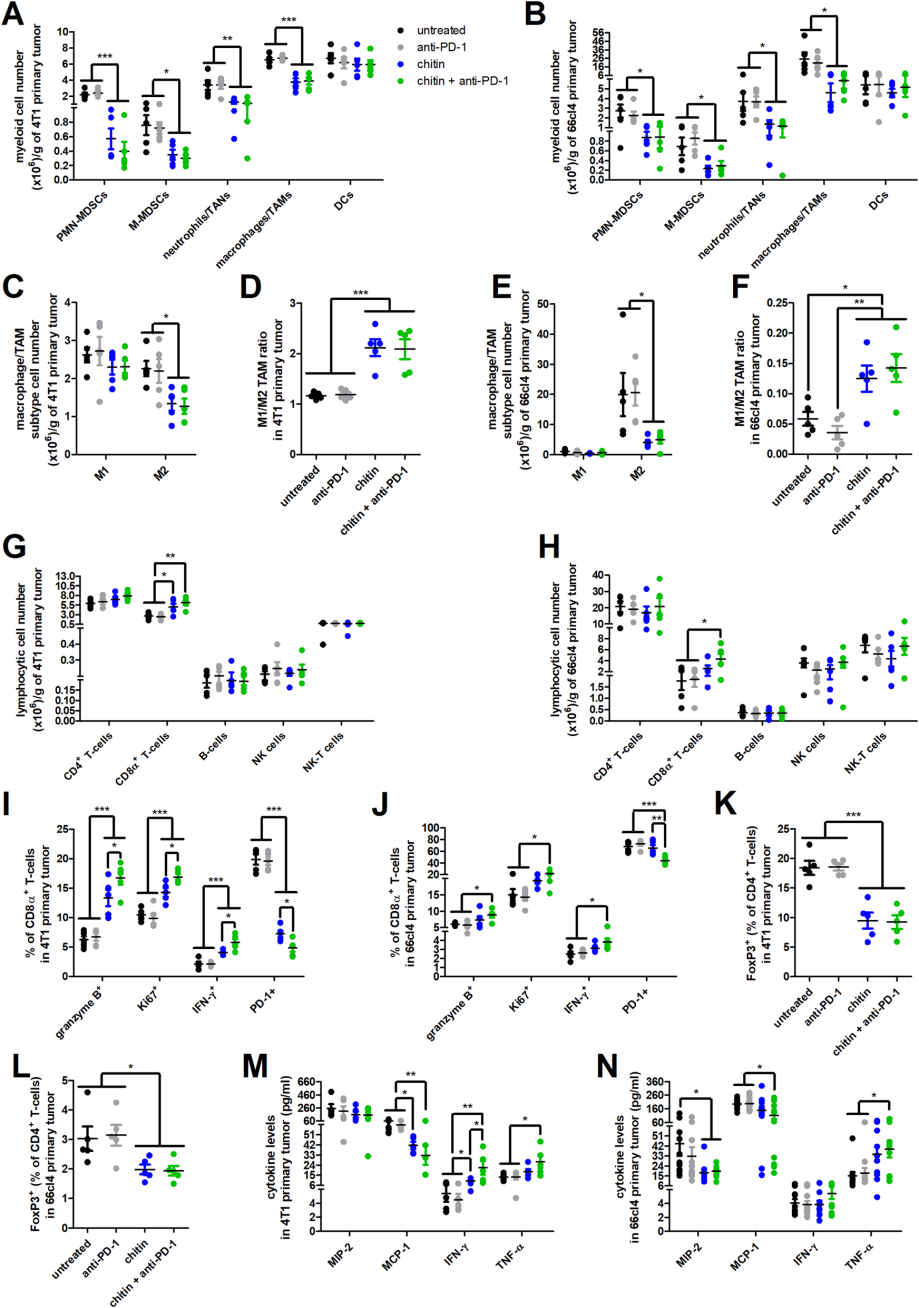
Chitin reduces immunosuppressive myeloid subpopulations and increases anti-PD-1-stimulated lymphocytic subpopulations in 4T1 and 66cl4 primary tumors. **A-L** Primary tumors were isolated from the untreated, anti-PD-1-, chitin- and chitin + anti-PD-treated 4T1- and 66cl4-based model at 5 w p.i. and processed into a single cell suspension for flow cytometric immunophenotyping (n = 5 for all groups). **A,B** Number of myeloid subpopulations (including PMN-MDSCs, M-MDSCs, TANs, TAMs and DCs) per gram of primary tumor in the untreated and treated 4T1- (**A**) and 66cl4-based model (**B**). **C,D** Number of M1 and M2 TAM subtypes per gram of primary tumor (**C**) and calculated M1/M2 TAM ratio (**D**) in the 4T1-based model. **E,F** Number of M1 and M2 TAM subtypes per gram of primary tumor (**E**) and calculated M1/M2 TAM ratio (**F**) in the 66cl4-based model. **G,H** Number of lymphocytic subpopulations (including CD4^+^ and CD8α^+^ T-cells, B-cells, NK cells and NK-T cells) per gram of primary tumor in the untreated and treated 4T1- (**G**) and 66cl4-based model (**H**). **I,J** Percentage of granzyme B^+^, Ki67^+^, IFN-γ^+^ and PD-1^+^ cells within the primary tumor CD8α^+^ T-cell population in the untreated and treated 4T1- (**I**) and 66cl4-based model (**J**). **K,L** Percentage of FoxP3^+^ cells within the primary tumor CD4^+^ T-cell population in the untreated and treated 4T1- (**K**) and 66cl4-based model (**L**). **M,N** Levels for MIP-2, MCP-1, IFN-γ and TNF-α in primary tumor lysates from the untreated and treated 4T1- (**M**) and 66cl4-based model (**N**) at 5 w p.i. (n = 5 for all groups in the 4T1-based model; n = 11 for all groups in the 66cl4-based model). Data are presented as the means ± SEM. *P < 0.05, **P < 0.01, ***P < 0.001

This immunophenotyping corroborated the Ly6G staining results on primary tumor sections, as chitin treatment in combination with or without anti-PD-1 significantly reduced both the number and percentage of TANs in the 4T1- and the number of TANs in the 66cl4-based model (Fig. 5A,B, Additional file 10: Fig. S8A,B). Chitin and chitin + anti-PD-1 treatment also significantly reduced the numbers/percentages of other major immunosuppressive myeloid subpopulations, including PMN-MDSCs, M-MDSCs and TAMs in the 4T1- and 66cl4-based model, albeit the reduction in PMN- and M-MDSC numbers within the chitin- and chitin + anti-PD-1-treated 66cl4 primary tumors was not reflected by a reduction in their percentages (Fig. 5A,B, Additional file 10: Fig. S8A,B). Moreover, although an increase in percentage, but not number, of DCs was detected upon chitin treatment with or without anti-PD-1 in 66cl4 primary tumors, these numbers/percentages were not affected in chitin- and chitin + anti-PD-1-treated 4T1 primary tumors (Fig. 5A,B, Additional file 10: Fig. S8A,B). Based on CD80 and MHCII as M1 TAM phenotype markers and CD206 as a M2 TAM phenotype marker, chitin and chitin + anti-PD-1 treatment significantly reduced the number of M2 TAMs without affecting M1 TAM numbers in both the 4T1 and 66cl4 primary tumors (Fig. 5C,E), thereby significantly increasing the M1/M2 TAM ratio (Fig. 5D,F). Chitin induced a similar significant reduction in immunosuppressive M2 TAM percentages upon combination with or without anti-PD-1 ICB in the 4T1 primary tumors, but only a non-significant reductive trend in the 66cl4 primary tumors (Additional file 10: Fig. S8C,D).

Further corroborating the immunophenotyping and reduced primary tumor immunosuppression, immunohistochemical staining for CD163 as M2 TAM marker was significantly reduced in chitin- and chitin + anti-PD-1-treated primary tumors compared to untreated and anti-PD-1-treated primary tumors from both models (Additional file 11: Fig. S9A). A striking difference was that this staining was typically confined to the tumor margin in 4T1 primary tumors, while located prominently in the tumor core of 66cl4 primary tumors (Additional file 11: Fig. S9A).

In marked contrast to the myeloid subpopulations, chitin treatment significantly increased the number/percentage of anti-tumorigenic CD8α^+^ T-cells in both 4T1 and 66cl4 primary tumors (Fig. 5G, Additional file 10: Fig. S8E). An increase in other lymphocytic subpopulations was also detected in the chitin-treated 66cl4 primary tumors, albeit only significantly in percentages of CD4^+^ T-cells, NK and NK-T cells (Additional file 10: Fig. S8F). Anti-PD-1 ICB provided add-on anti-tumorigenic immunostimulation and further increased the number/percentage of CD8α^+^ T-cells in chitin + anti-PD-1-treated compared to untreated and anti-PD-1-treated tumors in both the 4T1- and 66cl4-based model (Fig. 5G,H, Additional file 10: Fig. S8E,F). The chitin + anti-PD-1-treated 66cl4 primary tumors also showed an additional increase in CD4^+^ T-cell, CD8α^+^ T-cell, NK and NK-T cell percentages (Additional file 10: Fig. S8F). Besides an increase in numbers and percentages, chitin treatment enhanced CD8α^+^ T-cell activation in the 4T1 primary tumors with significantly increased positivity for granzyme B, Ki67 and IFN-γ, as well as significantly decreased positivity for the T-cell exhaustion marker PD-1 compared to untreated and anti-PD-1-treated tumors (Fig. 5I). Combining chitin with anti-PD-1 additionally activated CD8α^+^ T-cells in both 4T1 and 66cl4 primary tumors (Fig. 5I,J). Immunohistochemical staining for granzyme B on primary tumor sections corroborated the enhanced CD8α^+^ T-cell activation in primary tumors upon chitin treatment, albeit only significantly in the 4T1-based model (Additional file 11: Fig. S9B). Positivity was confined to the tumor core in both TNBC models, highlighting infiltration of activated lymphocytes. In line with the flow cytometric measurements, combining chitin with anti-PD-1 treatment further increased granzyme B staining in 4T1 primary tumors and now also significantly increased this staining in 66cl4 primary tumors compared to untreated and anti-PD-1 primary tumors (Additional file 11: Fig. S9B). Indicative for a reduced production of immunosuppressive T-regs, a significantly decreased forkhead box P3 (FoxP3) positivity within the CD4^+^ T-cell population was detected in chitin- and chitin + anti-PD-1-treated compared to untreated and anti-PD-1-treated 4T1 and 66cl4 primary tumors (Fig. 5K,L).

Cytokine profiling confirmed the reduction in immunosuppressive TANs upon chitin treatment, showing significantly reduced levels for MIP-2/CXCL2, an IL-8 orthologue and neutrophil chemoattractant, in chitin-treated 66cl4 primary tumor lysates and a reductive trend in chitin-treated 4T1 primary tumor lysates (Fig. 5M,N). In addition, significantly reduced levels for the monocyte chemoattractant MCP-1/CCL-2 corroborated the reduced M2 TAM numbers in chitin-treated 4T1 and 66cl4 primary tumors (Fig. 5M,N). Other myeloid subpopulation-supporting cytokines, including G-CSF, IL-1β, IL-4, IL-6, IL-10 and TGF-β1, were not significantly altered by chitin treatment either with or without anti-PD-1 in both 4T1 and 66cl4 primary tumors (Additional file 12: Fig. S10A,B). The enhanced T-cell activation in primary tumors upon chitin treatment was confirmed by significantly increased IFN-γ levels in the chitin-treated 4T1 primary tumors and an add-on increase was detected upon combining chitin with anti-PD-1 treatment (Fig. 5M). Chitin and anti-PD-1 combination treatment also increased IFN-γ levels—albeit not significantly—in 66cl4 primary tumor lysates (Fig. 5N) and significantly increased TNF-α levels in both the 4T1 and 66cl4 primary tumors (Fig. 5M,N).

**Fig. 6:**
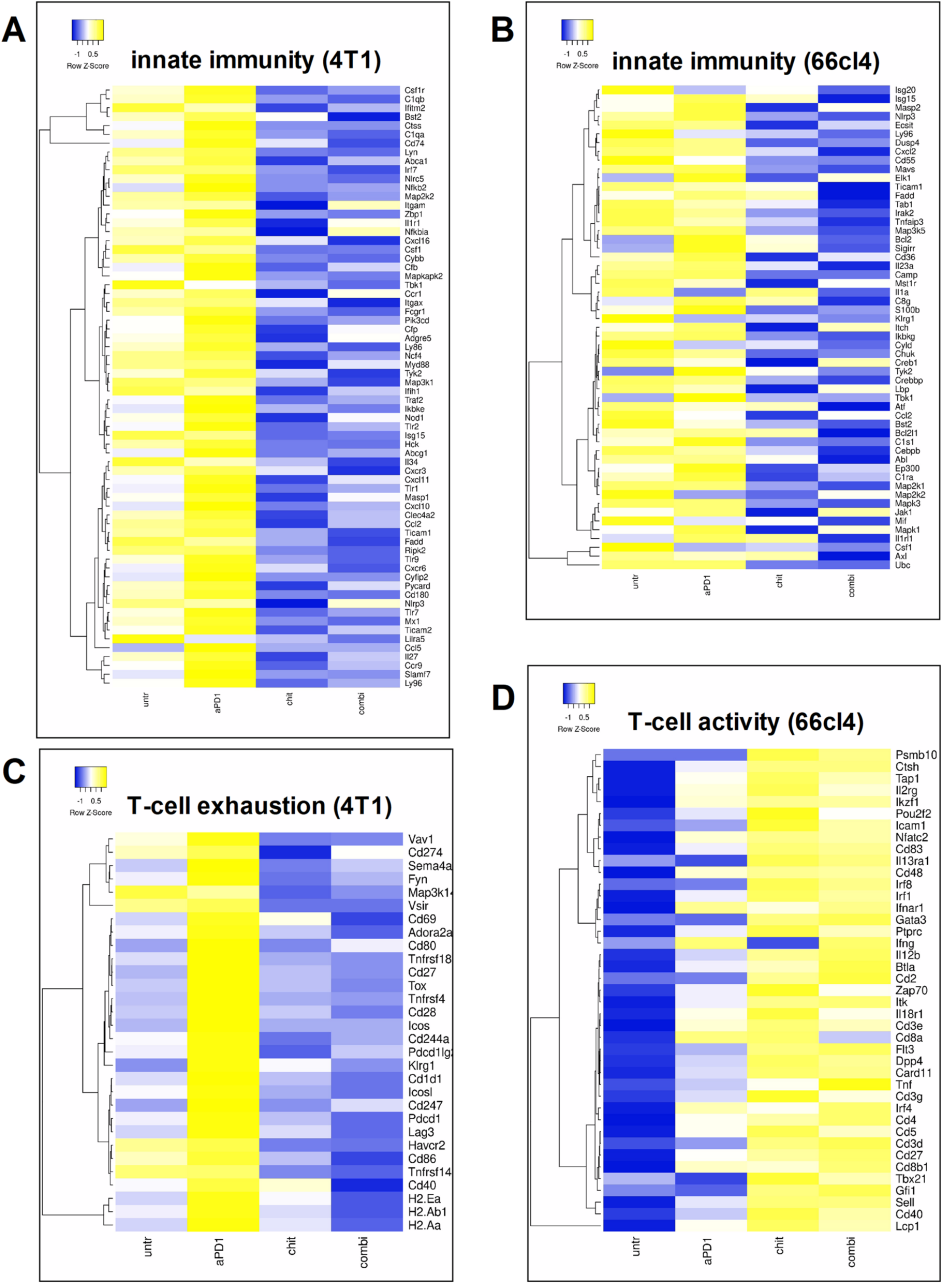
Innate- and T-cell-related gene expression levels corroborate the immunophenotypic changes upon chitin treatment in 4T1 and 66cl4 primary tumors. **A-D** Heatmaps showing mean normalized expression levels of selected genes associated with innate immunity in 4T1 (**A**) and 66cl4 primary tumors (**B**), T-cell exhaustion in 4T1 primary tumors (**C**) and T-cell activation in 66cl4 primary tumors (**D**) at 5 w p.i. derived from untreated, anti-PD-1, chitin- and chitin + anti-PD-1-treated tumor-bearing mice. Mean normalized expression levels were calculated based on normalized expression levels in 4 or 5 primary tumor samples from each treatment group and for each model. Selection of the genes was based on gene lists from the NanoString Mouse PanCancer Immune Profiling Panel. Pearson distance was used for hierarchical clustering

Chitin-mediated reduction in innate immunosuppression and increased anti-tumor T-cell immunity in primary tumors of both TNBC models was also clearly presented at the genomic level based on RNA-sequencing analysis. More specifically, a selection of 68 and 55 innate immunity-related genes in respectively 4T1 and 66cl4 primary tumors were downregulated upon chitin treatment in combination with or without anti-PD-1 (Fig. 6A,B). Several of the selected innate immunity genes could be linked to immunosuppressive myeloid cell types, including Ccl2, Cxcl2, Csf1r and Itgam. Genes related to T-cell exhaustion, including Pdcd1, Cd274, Havcr2 and Lag3, were downregulated upon chitin and chitin + anti-PD-1 treatment in 4T1 tumors (Fig. 6C). Chitin- and chitin + anti-PD-1-treated 66cl4 tumors showed upregulation of genes associated with enhanced T-cell activity, including Cd8a, Cd8b1, Ifng, Il12b and Il18r1 (Fig. 6D). In line with an overall reduced inflammation upon chitin treatment at the protein level, predominantly through myeloid cell reduction, a selected set of 68 and 48 inflammation-related genes were downregulated in chitin- and chitin + anti-PD-1-treated primary tumors of respectively the 4T1- and the 66cl4-based model (Additional file 13: Fig. S11A,B). Importantly, genes that have been associated with the signaling and pro-tumorigenic activity of CHI3L1 were also downregulated following chitin treatment either with or without anti-PD-1, including Usf1 [34] and Rab37 [35] in 4T1 primary tumors and Lgals3 [36] in 66cl4 primary tumors (Additional file 13: Fig. S11A,B).

### Chitin also enhances ICB immunostimulation in lymphoid organs of the 4T1- and 66cl4-based TNBC model

Flow cytometric immunophenotyping on the axillary lymph nodes and the spleen extended the local immunomodulation evaluation to lymphoid organs.

**Fig. 7:**
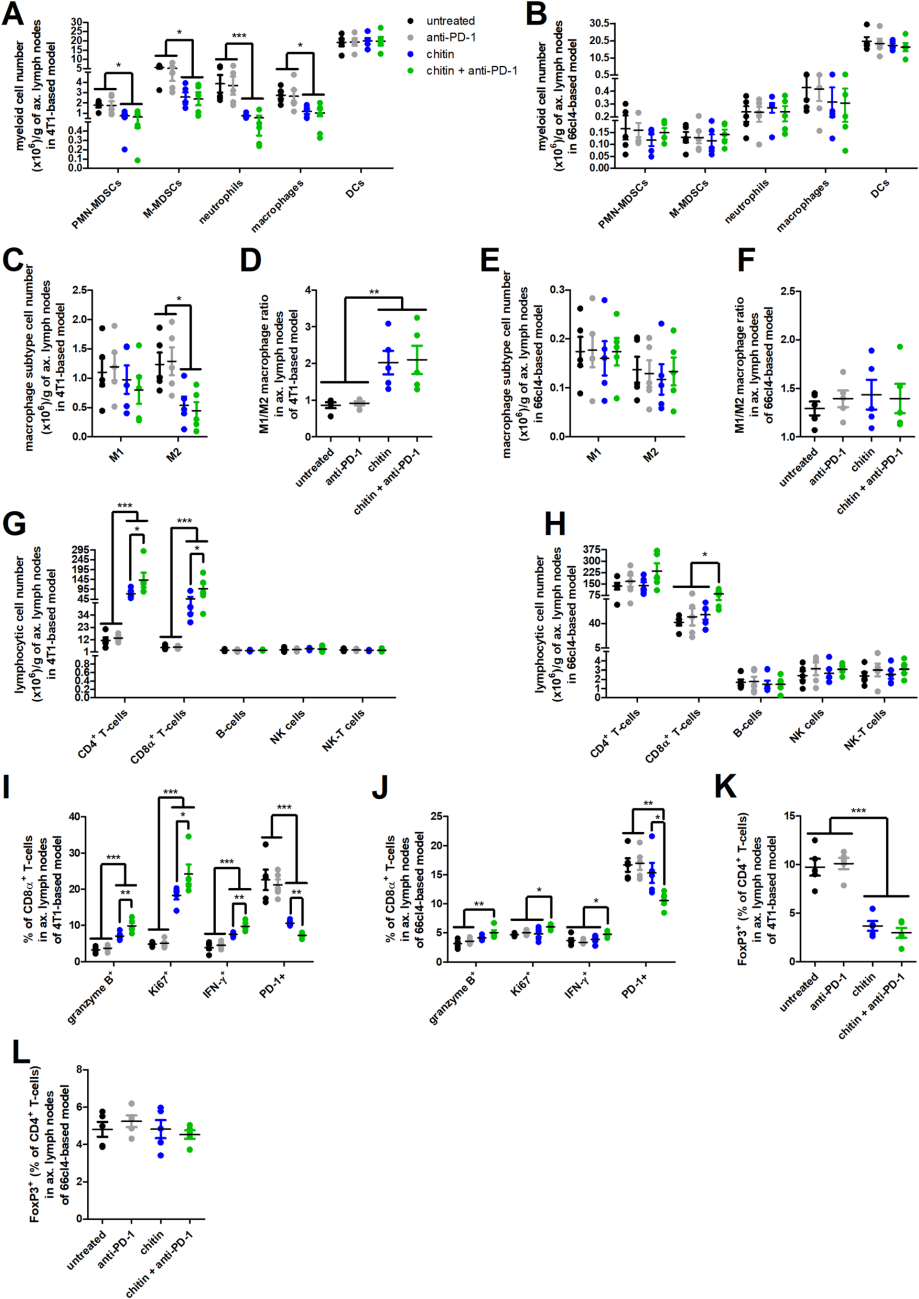
Chitin reduces immunosuppressive subpopulations in 4T1-derived axillary lymph nodes and increases anti-PD-1-stimulated lymphocytic subpopulations in both the 4T1- and 66cl4-derived axillary lymph nodes. **A-L** Axillary lymph nodes were isolated from the untreated, anti-PD-1-, chitin- and chitin + anti-PD-treated 4T1- and 66cl4-based model at 5 w p.i. and processed into a single cell suspension for flow cytometric immunophenotyping (n = 5 for all groups). **A,B** Number of myeloid subpopulations (including PMN-MDSCs, M-MDSCs, neutrophils, macrophages and DCs) per gram of axillary lymph nodes in the untreated and treated 4T1- (**A**) and 66cl4-based model (**B**). **C,D** Number of M1 and M2 macrophage subtypes per gram of axillary lymph nodes (**C**) and calculated M1/M2 macrophage ratio (**D**) in the 4T1-based model. **E,F** Number of M1 and M2 macrophage subtypes per gram of axillary lymph nodes (**E**) and calculated M1/M2 macrophage ratio (**F**) in the 66cl4-based model. **G,H** Number of lymphocytic subpopulations (including CD4^+^ and CD8α^+^ T-cells, B-cells, NK cells and NK-T cells) per gram of axillary lymph nodes in the untreated and treated 4T1- (**G**) and 66cl4-based model (**H**). **I,J** Percentage of granzyme B^+^, Ki67^+^, IFN-γ^+^ and PD-1^+^ cells within the axillary lymph node CD8α^+^ T-cell population in the untreated and treated 4T1- (**I**) and 66cl4-based model (**J**). **K,L** Percentage of FoxP3^+^ cells within the axillary lymph node CD4^+^ T-cell population in the untreated and treated 4T1- (**K**) and 66cl4-based model (**L**). Data are presented as the means ± SEM. *P < 0.05, **P < 0.01, ***P < 0.001

Similarly to the primary tumor, chitin treatment with or without anti-PD-1 again reduced the numbers of immunosuppressive myeloid PMN- and M-MDSC, neutrophil and macrophage, but not DC subpopulations in the axillary lymph nodes of the 4T1-based model (Fig. 7A). This myeloid cell reduction was also demonstrated through a significant reduction in M-MDSC and neutrophil percentages in the axillary lymph nodes of the 4T1-based model (Additional file 14: Fig. S12A). In marked contrast, axillary lymph nodes of the 66cl4-based model had low numbers/percentages of these myeloid subpopulations and either chitin monotherapy or chitin + anti-PD-1 combination treatment did not additionally reduce immunosuppression (Fig. 7B, Additional file 14: Fig. S12B). In the 4T1-based model, M1 macrophage numbers/percentages were not affected in axillary lymph nodes, but chitin monotherapy and chitin + anti-PD-1 combination treatment significantly decreased the M2 macrophage numbers and induced a reductive trend in M2 macrophage percentages (Fig. 7C, Additional file 14: Fig. S12C)), resulting in a significantly increased M1/M2 macrophage ratio (Fig. 7D). In the 66cl4-based model, both the M1 and M2 macrophage numbers/percentages were not affected by either treatment in axillary lymph nodes (Fig. 7E, Additional file 14: Fig. S12D), resulting in a similar M1/M2 macrophage ratio in all treatment groups (Fig. 7F).

In the 4T1-based model, both CD4^+^ and CD8α^+^ T-cell numbers/percentages significantly increased upon chitin treatment in axillary lymph nodes with a significant add-on increase upon combination of chitin with anti-PD-1 treatment (Fig. 7G, Additional file 14: Fig. S12E). Although axillary lymph nodes from untreated 66cl4 tumor-bearing mice already had a very high number/percentage of CD4^+^ and CD8α^+^ T-cells compared to the 4T1-based model, an additional significant increase in the number/percentage of CD8α^+^ T-cells and an increased—albeit not significantly—CD4^+^ T-cell number/percentage was also detected upon chitin + anti-PD-1 combination treatment (Fig. 7H, Additional file 14: Fig. S12F). The B-, NK- and NK-T cell numbers/percentages, did not significantly change upon chitin treatment either with or without anti-PD-1 in axillary lymph nodes of both the 4T1- and the 66cl4-based model (Fig. 7G,H, Additional file 14: Fig. S12E,F). Chitin again activated the CD8α^+^ T-cells in axillary lymph nodes of the 4T1-based model as shown by their significantly increased positivity for granzyme B, Ki67 and IFN-γ, as well as significantly decreased positivity for PD-1 compared to untreated and anti-PD-1-treated counterparts (Fig. 7I). Combining chitin with anti-PD-1 treatment significantly enhanced this CD8α^+^ T-cell activation compared to chitin monotherapy in the axillary lymph nodes, now both in the 4T1- and 66cl4-based model (Fig. 7I,J). FoxP3 positivity within the CD4^+^ T-cell population of the axillary lymph nodes significantly decreased by chitin either with or without anti-PD-1 treatment in the 4T1- but not in the 66cl4-based model (Fig. 7K,L).

**Fig. 8:**
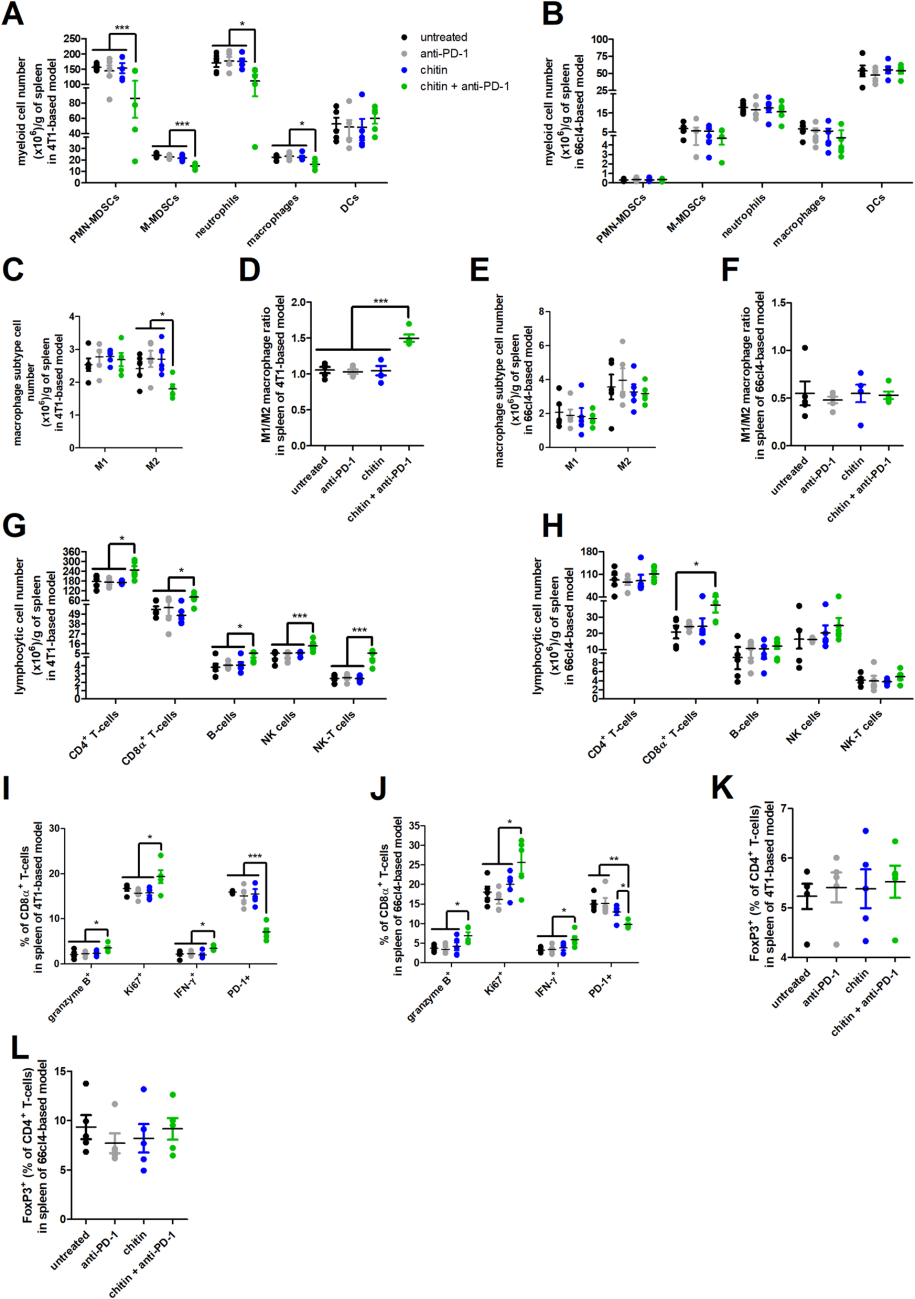
Chitin reduces immunosuppressive subpopulations in 4T1-derived spleens and increases anti-PD-1-stimulated lymphocytic subpopulations in both the 4T1- and 66cl4-derived spleens. **A-L** Spleens were isolated from the untreated, anti-PD-1-, chitin- and chitin + anti-PD-treated 4T1- and 66cl4-based model at 5 w p.i. and processed into a single cell suspension for flow cytometric immunophenotyping (n = 5 for all groups). **A,B** Number of myeloid subpopulations (including PMN-MDSCs, M-MDSCs, neutrophils, macrophages and DCs) per gram of spleen in the untreated and treated 4T1- (**A**) and 66cl4-based model (**B**). **C,D** Number of M1 and M2 macrophage subtypes per gram of spleen (**C**) and calculated M1/M2 macrophage ratio (**D**) in the 4T1-based model. **E,F** Number of M1 and M2 macrophage subtypes per gram of spleen (**E**) and calculated M1/M2 macrophage ratio (**F**) in the 66cl4-based model. **G,H** Number of lymphocytic subpopulations (including CD4^+^ and CD8α^+^ T-cells, B-cells, NK cells and NK-T cells) per gram of spleen in the untreated and treated 4T1- (**G**) and 66cl4-based model (**H**). **I,J** Percentage of granzyme B^+^, Ki67^+^, IFN-γ^+^ and PD-1^+^ cells within the splenic CD8α^+^ T-cell population in the untreated and treated 4T1- (**I**) and 66cl4-based model (**J**). **K,L** Percentage of FoxP3^+^ cells within the splenic CD4^+^ T-cell population in the untreated and treated 4T1- (**K**) and 66cl4-based model (**L**). Data are presented as the means ± SEM. *P < 0.05, **P < 0.01, ***P < 0.001

At the splenic level, the 4T1-based model showed a very high number/percentage of immunosuppressive PMN-MDSC and neutrophil subpopulations. In marked contrast to their reduction in the primary tumor and axillary lymph node compartments, chitin monotherapy did not affect PMN- and M-MDSC, neutrophil and macrophage numbers/percentages in the spleen of 4T1 tumor-bearing mice (Fig. 8A, Additional file 15: Fig. S13A), corroborating the comparable splenomegaly in the untreated, anti-PD-1- and chitin-treated 4T1-based model (Fig. 2D). However, combining chitin with anti-PD-1 treatment in the 4T1-based model significantly reduced the splenic numbers/percentages of these myeloid cells. DC numbers/percentages again remained unchanged (Fig. 8A, Additional file 15: Fig. S13A). Spleens from the 66cl4 tumor-bearing mice showed only very low numbers/percentages of the myeloid subpopulations (Fig. 8B, Additional file 15: Fig. S13B), corroborating the absence of splenomegaly in the 66cl4-based model (Fig. 2E).

Whereas splenic M1 macrophage numbers/percentages remained unchanged, a significant decrease in numbers and a reductive trend in percentages of M2 macrophages was detected in the spleen of the 4T1 tumor-bearing mice upon chitin + anti-PD-1 combination treatment (Fig. 8C, Additional file 15: Fig. S13C), resulting in a significantly increased M1/M2 macrophage ratio compared to the other treatment groups (Fig. 8D). In marked contrast, neither chitin nor chitin + anti-PD-1 combination treatment reduced splenic myeloid cell numbers/percentages in the 66cl4-based model, resulting in similar splenic M1 and M2 macrophage subtype numbers/percentages (Fig. 8E, Additional file 15: Fig. S13D) and M1/M2 macrophage ratio (Fig. 8F) compared to spleens from untreated and anti-PD-1-treated 66cl4 tumor-bearing mice.

In the spleen of 4T1 tumor-bearing mice, the numbers/percentages of the lymphocytic subpopulations CD4^+^ and CD8α^+^ T-cells, B-cells, NK and NK-T cells significantly increased in response to chitin + anti-PD-1 combination treatment (Fig. 8G, Additional file 15: Fig. S13E). Spleens from the chitin-treated 66cl4 tumor-bearing mice showed a significant increase in CD4^+^ T-cell percentages compared to spleens from untreated and anti-PD-1-treated 66cl4 tumor-bearing mice (Additional file 15: Fig. S13F). Chitin in combination with anti-PD-1 further enhanced these splenic CD4^+^ T-cell, CD8α^+^ T-cell and B-cell percentages in the 66cl4-based model (Additional file 15: Fig. S13F). The significant increase in splenic CD8α^+^ T-cell percentages in the chitin + anti-PD-1-treated compared to the untreated 66cl4 tumor-bearing mice was also reflected in the splenic CD8α^+^ T-cell numbers (Fig. 8H, Additional file 15: Fig. S13F). Both models showed a significantly enhanced CD8α^+^ T-cell activation in the spleen upon chitin + anti-PD-1 combination treatment, highlighted with an increased positivity for granzyme B, Ki67 and IFN-γ, and a decreased positivity for PD-1 in the splenic CD8α^+^ T-cell population (Fig. 8I,J). T-reg production was not affected by either treatment in the spleen of both models based on similar FoxP3 positivity within the CD4^+^ T-cell population (Fig. 8K,L).

### Chitin enhances tumor growth reduction and anti-tumor immunity compared to single CLP blockade

**Fig. 9:**
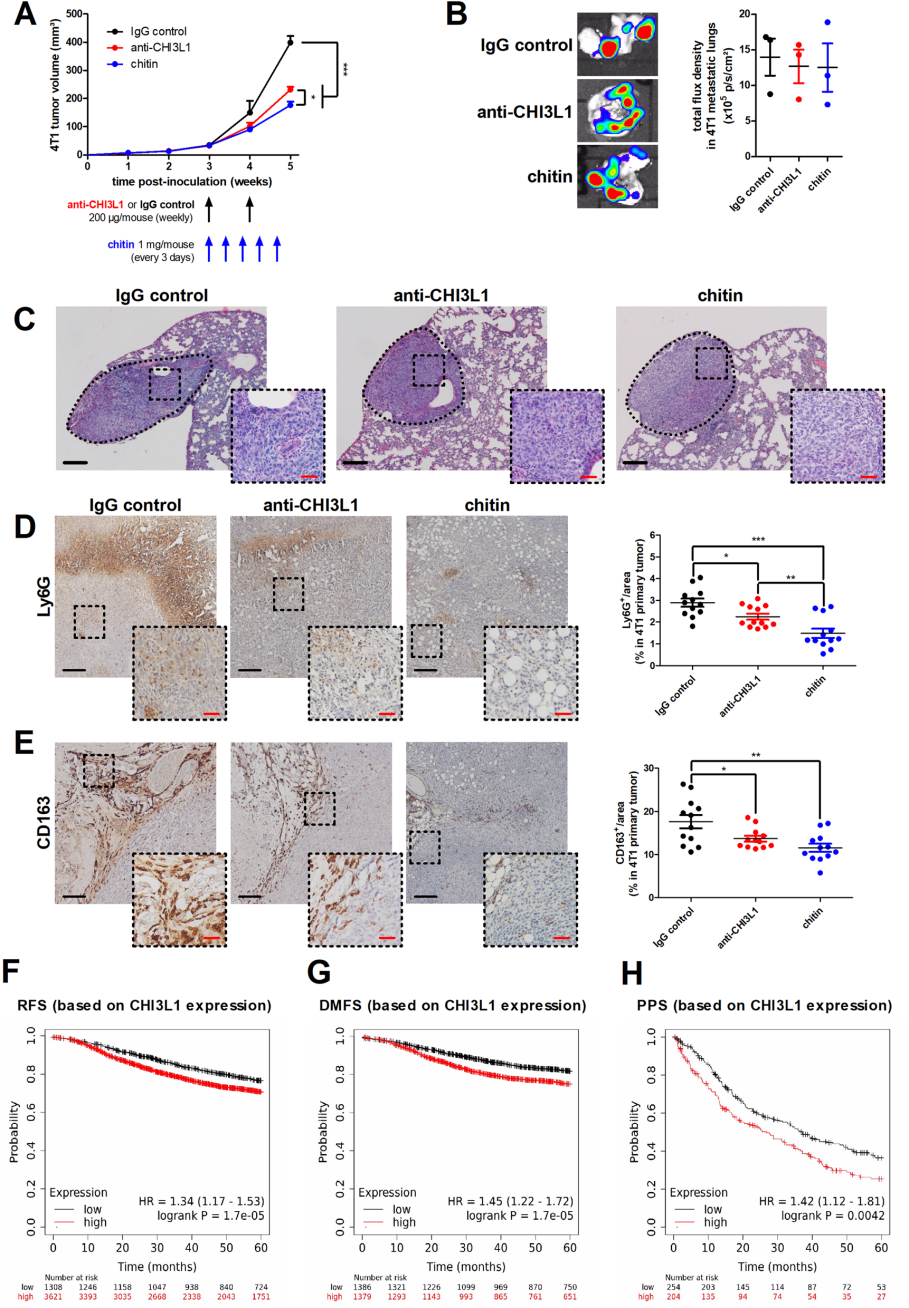
Chitin provides enhanced tumor reduction and anti-tumorigenicity compared to anti-CHI3L1 treatment in the 4T1-based intraductal model. **A** Weekly measurements of primary tumor volumes in the IgG control-, anti-CHI3L1- and chitin-treated 4T1-based model with treatment schedules indicated (n = 6 for all groups at all time points). **B** Representative images and quantification of bioluminescent signals (total flux density in p/s/cm^2^) in lungs from the IgG control-, anti-CHI3L1 and chitin-treated 4T1-based model at 5 w p.i. (n = 3 for all groups). **C** H&E histology of lung metastases from the IgG control-, anti-CHI3L1- and chitin-treated 4T1-based model at 5 w p.i. Dashed inserts highlight H&E-stained metastases at a larger magnification. **D,E** Immunohistochemistry for the PMN-MDSC/TAN marker Ly6G (**D**) and the M2 TAM subtype marker CD163 (**E**) on primary tumor sections from IgG control-, anti-CHI3L1- and chitin-treated 4T1 tumor-bearing mice at 5 w p.i. (n = 12 for all groups; 3 slides with 4 images per slide). Dashed inserts highlight stained tissue at a larger magnification. Black scale bars = 200 µm, red scale bars = 50 μm. **F–H** Kaplan Meier plots showing relapse-free (**F**), distant metastasis-free (**G**) and post-progression survival (**H**) over 60 months time based on CHI3L1 expression as calculated using publicly available mRNA gene chip data from all BC subtypes and the KM-plotter tool. Number of patients that were included for analysis: 4929 for RFS, 2765 for DMFS and 458 for PPS. Patients were split in high/low CHI3L1 expressors based on auto select of the best cutoff by the KM-plotter tool. Data are presented as the means ± SEM. *P < 0.05, **P < 0.01, ***P < 0.001

Given that chitin neutralizes multiple CLP family members, especially in the 4T1-based model, we subsequently investigated whether this general CLP blockade outperforms single CLP blockade in terms of disease reduction. Therefore, 4T1 tumor-bearing mice were treated with either chitin (every 3 d), anti-CHI3L1 or IgG control antibodies (weekly) for 2 w until 5 w p.i., and tumor progression was monitored through tumor volume measurements (Fig. 9A). Anti-CHI3L1 significantly reduced tumor growth compared to IgG control treatment, but chitin showed a significant add-on treatment effect compared to anti-CHI3L1 (Fig. 9A). Lung metastases were not significantly impacted by either treatment (Fig. 9B,C).

Immunohistochemistry identified significantly reduced Ly6G^+^ TAN and CD163^+^ M2 TAM stainings on primary tumor sections from anti-CHI3L1- compared to IgG control-treated mice, and add-on reductions of both markers in chitin-treated primary tumors (Fig. 9D,E). Flow cytometric immunophenotyping confirmed significantly reduced numbers of PMN-MDSCs and TANs in anti-CHI3L1-treated primary tumors, and again showed additional reduction in the number of TANs and M-MDSCs upon chitin treatment (Additional file 16: Fig. S14A). These changes were also shown in percentages, albeit not statistically significantly (Additional file 16: Fig. S14B). Corroborating the CD163 stainings, anti-CHI3L1 treatment significantly increased the M1/M2 TAM ratio, whereas chitin treatment provided a significant add-on increase in this ratio through additional reduction of M2 TAM numbers/percentages (Additional file 16: Fig. S14C). Although anti-CHI3L1 treatment did not significantly upregulate CD8α^+^ T-cell numbers/percentages (Additional file 16: Fig. S14D,E), their activation was significantly increased by the treatment based on decreased positivity for PD-1 and enhanced positivity for granzyme B, Ki67 and IFN-γ (Additional file 16: Fig. S14F). Chitin, on the other hand, provided an add-on increase in the CD8α^+^ T-cell numbers and significantly in percentages (Additional file 16: Fig. S14D,E), also inducing an add-on increase in their activation compared to anti-CHI3L1 treatment based on a significantly decreased positivity for PD-1 (Additional file 16: Fig. S14F). Other CD8α^+^ T-cell activation markers, including granzyme B, Ki67 and IFN-γ were not significantly increased with chitin compared to anti-CHI3L1 treatment. T-reg production was not significantly reduced by anti-CHI3L1 treatment, but chitin provided an add-on significant reduction compared to IgG control and anti-CHI3L1 based on FoxP3 positivity in the CD4^+^ T-cell population (Additional file 16: Fig. S14G).

Our preclinical data on the immunostimulatory role of CLPs, and more specifically CHI3L1, in BC progression could also be translated to the human patient based on publicly available datasets [37]. High gene expression levels of CHI3L1 are a predictor for significantly reduced relapse-free, distant metastasis-free and post-progression survival (Fig. 9F-H). Moreover, CHI3L1 is amplified in 12–24% of primary tumors in all BC subtypes and in 6–12% of primary tumors in TNBC based on data from The Cancer Genome Atlas/TCGA and Molecular Taxonomy of Breast Cancer International Consortium/METABRIC (Additional file 17: Fig. S15A). Upon metastatic BC, these frequencies range from 12–15% in all BC subtypes and 8–34% in TNBC (Additional file 17: Fig. S15B). Supporting its importance in TNBC, CHI3L1 gene expression significantly negatively correlates with ESR1, PGR and ERBB2 gene expression levels in human BC (Additional file 17: Fig. S15C). Further translating our preclinical mouse data, CHI3L1 gene expression significantly positively correlates with the expression of the myeloid cell markers ITGAM, CD14, MPO, CD68 and CD163, the immune checkpoint protein PDCD1 and the T-reg marker FoxP3 (Additional file 17: Fig. S15D,E).

### Chitin reduces lymphatic metastasis and inhibits CHI3L1-stimulated lymphatic vessel integration of macrophages

**Fig. 10:**
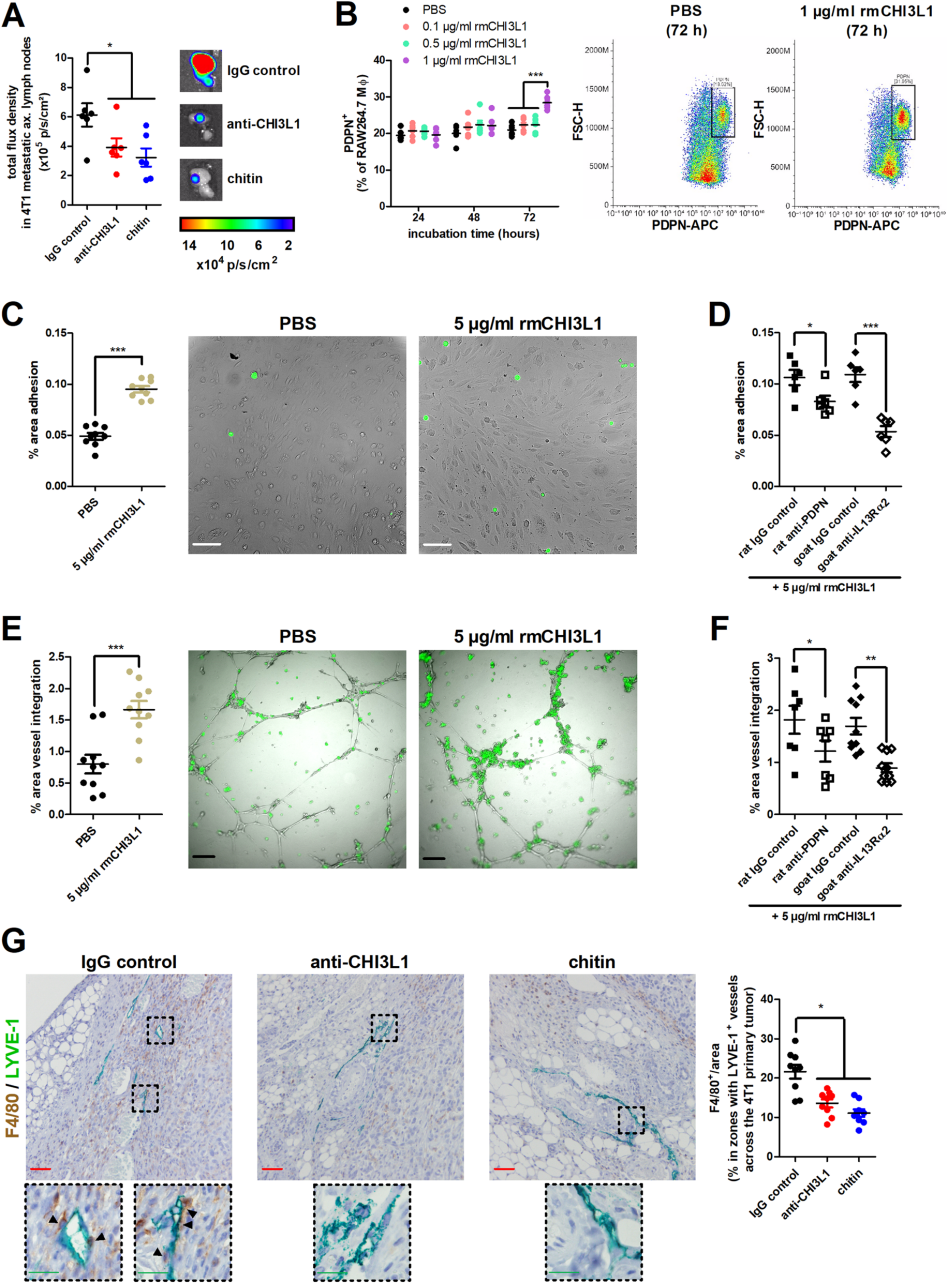
Chitin and anti-CHI3L1 treatment reduces CHI3L1-stimulated macrophage integration in lymphatic vessels in the 4T1-based intraductal model. **A** Representative images and quantification of bioluminescent signals (total flux density in p/s/cm^2^) in axillary lymph nodes from the IgG control-, anti-CHI3L1- and chitin-treated 4T1-based model at 5 w p.i. (n = 6 for all groups). **B** Flow cytometric analysis of PDPN positivity on the cell surface of RAW264.7 macrophages following a 24 h, 48 h and 72 h incubation with either PBS, 0.1, 0.5 or 1 µg/ml rmCHI3L1 (n = 6 for all groups). The dot plots highlight the PDPN positivity after a 72 h incubation with PBS versus 1 µg/ml rmCHI3L1. **C** Adhesion of PBS- and 5 µg/ml rmCHI3L1-treated RAW264.7 macrophages to a 2D LEC monolayer with representative images shown (n = 9 for all groups). **D** Ability of anti-PDPN, anti-IL13Rα2 and their respective IgG controls to inhibit adhesion of 5 µg/ml rmCHI3L1-treated RAW264.7 macrophages to a 2D LEC monolayer (n = 6 for all groups). **E** Integration of PBS- and 5 µg/ml rmCHI3L1-treated RAW264.7 macrophages into 3D LEC vessel-like structures with representative images shown (n = 9 for all groups). **F** Ability of anti-PDPN, anti-IL13Rα2 and their respective IgG controls to inhibit integration of 5 µg/ml rmCHI3L1-treated RAW264.7 macrophages into 3D LEC vessel-like structures (n = 7 for the rat IgG control and rat anti-PDPN group, n = 10 for the goat IgG control and goat anti-IL13Rα2 group). **G** Dual staining for the TAM marker F4/80 and LEC marker LYVE-1 on primary tumor sections from IgG control-, anti-CHI3L1- and chitin-treated 4T1 tumor-bearing mice at 5 w p.i. (n = 9 for all groups; 3 slides with 3 images per slide). Dashed inserts highlight stained tissue at a larger magnification. Black scale bars = 200 µm, white scale bars = 100 µm, red scale bars = 50 μm, green scale bars = 20 µm. Data are presented as the means ± SEM. *P < 0.05, **P < 0.01, ***P < 0.001

Neither anti-CHI3L1 nor chitin treatment affected hematogenous metastases to the lungs, but both treatment strategies equally and significantly decreased axillary lymph node metastases in 4T1 tumor-bearing mice (Fig. 10A), despite their differential effect on 4T1 primary tumor growth. Previous studies have shown that semaphorin 7a (SEMA7A), as upstream regulator of CHI3L1 [38], stimulates macrophage/TAM integration into lymphatic vessels to facilitate lymphatic entry and spreading of tumor cells [28]. However, the role of CHI3L1 in macrophage-mediated lymphatic remodeling has not yet been reported. Murine RAW264.7 macrophages incubated with rmCHI3L1 showed significantly increased surface expression of the lymphatic anchorage marker PDPN after 72 h (Fig. 10B). In line with an enhanced ability for PDPN-mediated lymphatic anchorage, CHI3L1-stimulated RAW264.7 macrophages showed a significantly higher adhesion to 2D monolayers of LECs (Fig. 10C), which could be countered through specific blockade of either PDPN or the cognate CHI3L1 receptor IL13Rα2 on the macrophage surface (Fig. 10D). CHI3L1-stimulated RAW264.7 macrophages also showed significantly increased integration into 3D lymphatic vessel structures (Fig. 10E), which again could be blocked by incubating the macrophages with anti-PDPN or anti-IL13Rα2 (Fig. 10F).

Translating these in vitro observations to our preclinical TNBC model, dual color immunohistochemistry for the LEC marker lymphatic vessel endothelial hyaluronan receptor 1 (LYVE-1) and the TAM marker F4/80 on primary tumor sections from 4T1 tumor-bearing mice further demonstrated the CHI3L1-mediated stimulation on lymphatic vessel integration of macrophages (Fig. 10G). More specifically, F4/80^+^ TAMs were found near and integrated into LYVE-1^+^ lymphatics in IgG control-treated primary tumors, whereas both anti-CHI3L1 and chitin treatment significantly reduced the association of TAMs with lymphatic structures in 4T1 primary tumors (Fig. 10G).

## Discussion

Cancer immunosuppression remains a major hurdle for immunotherapeutic efficacy in TNBC patients, and consequently a high clinical demand exists for novel therapeutic targets that can improve ICB outcome [4, 5]. Although the enzymatically-inactive CLP family has been identified to evolutionary fuel immunosuppression in wound healing [6, 7], mechanistic data on its contribution in highly metastatic BC such as TNBC associated with an immunosuppressed TME remain scarce. The most investigated CLP family member in the context of cancer is CHI3L1, being of specific interest due to its conserved expression in both mice and humans [11]. The strategy of blocking CHI3L1 with specific antibodies has been used in mouse models to decrease progression of melanoma [38–40], glioblastoma [41], lung [35, 42], pancreatic [35], colon [35] and breast cancer [16]. In marked contrast, the immunomodulatory effects envisaged after a more general blocking strategy of CLPs, especially in cancer and TNBC, have merely been investigated after chitin was twice opted for this purpose by the same group more than a decade ago [20, 21]. Indeed, the homoglycan chitin is the natural ligand of CLPs that functions as structural component of the fungal as well as bacterial cell wall, and is also present in the exoskeleton of nematodes, insects and crustaceans, but is not synthesized by mammals [6]. Given the enzymatic inactivity of CLPs, they bind but cannot cleave chitin, resulting in their non-selective blockade. We here explored at first the anti-cancer immunomodulatory effects of chitin monotherapy and its efficacy in combination with ICB in complementary intraductal mouse models for TNBC.

To establish the 4T1- and 66cl4-based intraductal model, we used lactating female mice that allow easy access to the teat canal and do not necessitate microscopic guidance or surgery for intraductal inoculation of tumor cells as described by other groups [43–45]. Moreover, the accompanying involution process is fueled by Stat3-driven CLP overexpression [46], providing a targetable environment for chitin treatment and allowing to investigate therapeutic efficacy in the context of pregnancy-associated BC, a rarely studied but aggressive BC with often TNBC characteristics [47].

Systemic injection of a chitin suspension significantly reduced progression of primary tumors but not lung metastases in the 4T1-based model. However, when extending these observations to a second intraductal TNBC model based on the also highly metastatic 66cl4 cell line, primary tumor growth and lung metastatic progression were not significantly reduced. At least partly explaining this important difference in chitin monotherapy effects between both TNBC models, is our key finding on the heterogenous production of CLP family members in these models. Whereas 4T1 primary tumors were demonstrated to be CLP-driven and showed high levels of both CHI3L1 and CHI3L3 as most prominent CLPs, the 66cl4 primary tumors were characterized to have only low to moderate production of these two major CLPs, respectively. We here at first unequivocally showed that in 4T1 primary tumors and serum, chitin significantly reduces CHI3L1 as well as CHI3L3 levels, while in 66cl4 primary tumors and not in serum, it significantly reduces only CHI3L3 levels. Of note, although their function in the context of cancer remains unclear, it should be mentioned that enzymatically active mammalian chitinases, including acidic mammalian chitinase (AMCase) and chitotriosidase (CHIT1), are also known targets of chitin [6] and could potentially contribute to the observed treatment effects in the 4T1- and 66cl4-based model for TNBC. However, neither of these mammalian chitinases were detected by RNA-seq and therefore very likely do not play a significant role in our models.

Our second key observation was the source of these CLPs. Based on immunohistochemistry and separation of Ly6G^−^ and Ly6G^+^ cell fractions, our data identify immune cells, and more specifically TANs, as prominent CLP producers, which corroborates the findings of a few reports in a non-cancer context [48–50], but also differs from previous cancer literature [20, 36, 39, 51]. These differential findings highlight the model- and disease-dependent sources of CLPs. It should be emphasized that our Ly6G^+^ cell separation experiments did not specify between TANs and PMN-MDSC precursors, leaving the question open whether the mature and/or precursor Ly6G^+^ cell population can be regarded as the primary CLP source in the 4T1- and 66cl4-based model. Of relevance, the PMN-MDSC-mediated production of CHI3L1 has been highlighted once in 2018, also in the context of TNBC [52]. Yet, the Ly6G^+^ nature of prominent CLP producers at least provides an explanation for the heterogeneity in CLP production between the 4T1- and 66cl4-based model: whereas the 4T1-based model is characterized by high numbers/percentages of PMN-MDSCs and TANs in its TME and concomitant G-CSF and MIP-2 primary tumor levels, the 66cl4-based model has only low numbers/percentages of both granulocytic subpopulations and related cytokine levels in its primary tumors.

In order to gain further mechanistic insights into how chitin-mediated CLP blockade eventually reduces CLP production, we investigated a reported positive feedback loop that involves CHI3L1 and p-Stat3, regulating CHI3L1 production [31–33]. As a third key finding, we here show that chitin-mediated CHI3L1 blockade impacts this feedback loop by inhibiting CHI3L1 signaling and Stat3 phosphorylation, reducing local and systemic CHI3L1 levels, especially in the high CLP-producing 4T1-based model. Whether CHI3L3 is also regulated by such a feedback loop remains unclear. However, the reduced CHI3L1 and/or CHI3L3 levels upon chitin treatment in both TNBC models may also be—at least partly—explained by alternative processes. Indeed, a 2023 pioneer study in the context of TNBC showed at first that CHI3L1 functions as a direct chemoattractant for TANs [51], suggesting that CHI3L1 blockade inhibits TAN recruitment and subsequently reduces CLP levels. Therefore, the reduced CLP levels upon chitin treatment could be intricately linked to the number of recruited TANs, a hypothesis that is corroborated by our flow cytometric data for TANs (and precursors), a reduced immunohistochemical staining for Ly6G and a reduced expression of CHI3L1-associated genes in chitin-treated primary tumors.

Despite the differential decreases in CLP levels, both ICB-resistant TNBC models showed a similarly enhanced response to anti-PD-1 immunotherapy following chitin treatment. More specifically, a combination treatment of chitin with anti-PD-1 provided a significant and add-on growth reduction in both the 4T1- and 66cl4-based model, not only limited to primary tumors but also extended to lung metastases. In line with these data, a synergistic effect between anti-CHI3L1 and either anti-PD-1 or anti-cytotoxic T-lymphocyte antigen (CTLA)-4 ICB has recently been described for melanoma lung metastases [39, 40]. Importantly, only the levels of CHI3L3, the CLP that is in general more produced than CHI3L1 in the murine TME, were high enough in the lower CLP-producing 66cl4 primary tumors to be impacted by chitin treatment. Nonetheless, the chitin-mediated reduction of these moderate CHI3L3 levels still sufficed to concomitantly increase anti-PD-1 efficacy. These key results highlight the potential for a broad use of chitin to stimulate ICB efficacy in TNBC, even in cases with low to moderate CHI3L1 and CHI3L3 production in the TME based on the combined presence of TANs and their precursors.

Previous reports on multiple cellular targets of CLPs, and especially CHI3L1, in the context of cancer could further explain the immune cell changes observed in our mouse models upon chitin-mediated CLP blockade. More specifically, CHI3L1 binds and signals through its cognate receptor IL13Rα2 on macrophages to induce tumor-promoting M2 polarization [35, 36, 39, 53], enhance PD-L1 expression on the macrophage surface [39] and stimulate production of pro-inflammatory factors MIP-2 and MCP-1 [20], respectively attracting neutrophils and additional macrophages to the TME for immunosuppression. The cited 2023 pioneer study additionally showed that CHI3L1 serves as a TAN chemoattractant [51], which in turn reduces TAN(-precursor) infiltration in the primary tumor and promotes T-cell exclusion in the TME through formation of NETs. The latter mechanistic aspect is supported by our study based on reduced levels of MPO—a NET marker—in chitin-treated 4T1 and 66cl4 primary tumors. Of note, the reduction in PMN-MDSCs and TANs is clinically highly relevant since these related myeloid subpopulations remain difficult to target with (chemo)therapeutics and are associated with immunotherapeutic failure [54, 55]. Furthermore, CHI3L1 directly reduces the anti-tumorigenic T-cell production of IFN-γ and abrogates Th1 polarization as well as cytotoxic T-lymphocyte activation [15, 20], which can be inhibited by chitin treatment. Production of immunosuppressive T-regs in the TME has been reported to be stimulated by CHI3L1 and can consequently be inhibited by CHI3L1 blockade [35]. Moreover, our observed significant decrease in T-reg production upon chitin treatment again further emphasizes the beneficial effect on ICB efficacy for human TNBC patients since T-reg amplification upon anti-PD-1 treatment promotes therapeutic resistance [56, 57]. Besides providing suppressive modulation of the host immune system, CHI3L1 has a direct impact on tumor cells by activating pathways involved in cellular proliferation, migration, stemness and survival [13, 26, 35, 58–60].

The chitin-mediated immunostimulation was not restricted to the primary tumor, since it also influenced systemic immune populations in lymphoid organs. Again, especially in the 4T1-based model, a reduction of cancer immunosuppression through significant decrease of myeloid cell types and concomitant increase in lymphocytic cell types was observed in both the axillary lymph nodes and the spleen. The 4T1-based model is known to display excessive myeloid cell production, also referred to as extramedullary hematopoiesis (EMH), in distant organs and most prominently in the spleen [61]. Given the stimulatory role of 4T1 cells in the EMH process through secretion of myeloid cell-supporting cytokines such as G-CSF [30, 62, 63], splenomegaly in this model has been linked to primary tumor growth and disease progression [8, 30]. Yet, despite a chitin-mediated reduction in primary tumor growth, spleen size and associated splenic myeloid cell numbers were not affected upon chitin monotherapy and required a chitin and anti-PD-1 combination for their significant reduction. A potential explanation for this apparent discrepancy in spleen and tumor size upon chitin treatment in the 4T1-based model is that the remaining lung metastases further support EMH through myeloid cell-supporting cytokines and that chitin treatment is not able to compensate for the latter massive myeloid cell production. In marked contrast, a chitin + anti-PD-1 combination treatment was able to almost completely prevent metastatic growth with accompanying reduction in splenomegaly. Again strikingly different from the 4T1-based model, the 66cl4-based model showed no expansion of myeloid cell numbers in either untreated or anti-PD-1-treated axillary lymph nodes and spleens, the latter being supported by a normal spleen size. The potential explanation for this absence of EMH is the much lower secretion of myeloid cell-supporting cytokines, corroborating other reports on the 66cl4-based model [63, 64]. Another salient finding was that, despite this restricted systemic immunosuppression, the 66cl4-based model remains resistant to ICB and requires anti-PD-1 to be combined with chitin-mediated CLP blockade to expand CD8α^+^ T-cell numbers and their activation in the axillary lymph nodes and spleen, supporting systemic disease reduction.

Besides identifying the benefits of combining CLP blockade with ICB, our study also highlights at first that a chitin-mediated general blockade is more preferable than blocking a single CLP, e.g. CHI3L1, with specific antibodies. Indeed, general CLP blockade provided an enhanced reduction in primary tumor growth compared to anti-CHI3L1 treatment in the 4T1-based model, reduced immunosuppressive myeloid populations and T-reg production to a higher extent, and also increased anti-tumorigenic CD8α^+^ T-cell numbers and their activity to a higher level. Comparing chitin and anti-CHI3L1 treatment in the context of TNBC additionally highlighted at first a potential mechanism by which CHI3L1 blockade prevents TAMs from enhancing metastases in the axillary lymph nodes. These findings are in line with a previous study that investigated the stimulatory role of SEMA7A as upstream regulator of CHI3L1 in macrophage-mediated lymphatic remodeling [28], a process by which macrophages adhere and integrate into the lymphatic vessel wall to promote lymphatic metastasis. Moreover, these novel findings are supported by other groups that identified the upregulation of PDPN as lymphatic anchorage marker by CHI3L1 in macrophages [65], as well as reduction in lymphatic spreading of B16-BL6 melanoma cells upon treatment with anti-CHI3L1 antibodies [39].

Based on the amplification frequency of CHI3L1, which is highest in metastatic TNBC patient samples, and the positive correlation of CHI3L1 expression with immunosuppressive markers, we hypothesize that CLP blockade could provide clinical benefits and enhance response to ICB in TNBC. Although this clinical translation will necessitate treatment optimization and additional toxicity testing in subsequent trials, earlier reports on chitin treatment in human patients were promising. In fact, chitin oligosaccharide mixtures have already been commercially available in Asia since 1990’s and significant tumor regression in early stage cancer has been anecdotically observed following *per os* administration in a number of cases [66]. Moreover, chitin is often used to develop nanoparticle carriers for drug delivery [67]. The role of chitin in the eventual therapeutic efficacy is neglected in these reports, but can be important based on its immunomodulatory and anti-metastatic effects as observed in our study. The anti-cancer effects of chitin also crucially depend on the chitin particle size and degree of acetylation as large particles were reported to induce M2 macrophage polarization [68, 69] and chitosan, the deacetylated form of chitin, is less efficient in mediating M1 macrophage activation [68, 69].

## Conclusions

Our preclinical data show that chitin blocks and reduces the levels of CHI3L1 and CHI3L3 as most prominent CLP family members in complementary ICB-resistant intraductal mouse models for TNBC. Chitin-mediated CLP blockade significantly reduces immunosuppressive cell types and stimulates anti-PD-1 efficacy with concomitant decrease in disease progression and increase in anti-tumorigenic lymphocytes both in the primary tumor and lymphoid organs. Moreover, chitin-mediated blockade of CLPs, and especially CHI3L1, also prevents macrophages from stimulating the outgrowth of metastases in axillary lymph nodes, which remain the earliest metastatic sites in the context of BC and aggressive TNBC. Overall, we identify chitin as a potentially tolerable treatment to reduce immunosuppression and lymphatic metastasis in ICB-resistant TNBC patients. Given its significant immunostimulatory effects in low CLP-producing TNBC models, chitin could even be effective beyond TNBC patients that show CLP overexpression.

### Supplementary Information

**Additional file 1:** **Figure S1.** Validation of chitin particle size and acetylation. (A) Flow cytometric analysis of particle sizes in the chitin suspension for in vivo use with 1 and 9.9 µm microspheres indicated for size reference. (B) MALDI-TOF MS verifies the degree of polymerization and acetylation of the chitin suspension as previously reported [24].

**Additional file 2:** **Table S1.** Primary antibodies used for immunohistochemistry.

**Additional file 3:** **Table S2.** Fluorophore-conjugated antibodies used for flow cytometric immunophenotyping.

**Additional file 4:** **Figure S2.** Chitin in combination with and without anti-PD-1 treatment has no toxic effect in a 4T1- and 66cl4-based intraductal model. (A,B) Weekly body weight measurements for animal welfare monitoring of the untreated and treated 4T1- (A) and 66cl4-based model (B) (n = 7 for all groups at all time points in the 4T1-based model; n = 11 for all groups at all time points in the 66cl4-based model). (C,D) Weekly body temperature measurements for animal welfare monitoring of the untreated and treated 4T1- (C) and 66cl4-based model (D) (n = 7 for all groups at all time points in the 4T1-based model; n = 11 for all groups at all time points in the 66cl4-based model). The decrease in body weight in both intraductal models during the first w p.i. can be attributed to cessation of milk production after pup weaning. Data are presented as the means +/- SEM.

**Additional file 5:** **Figure S3.** The treatment effect of a chitin and anti-PD-1 combination is anti-PD-1 dose-dependent in a 4T1-based intraductal model. (A) Weekly measurements of primary tumor volumes in the untreated, 25 µg IgG control-, 25 µg anti-PD-1-, chitin- and chitin + 25 µg anti-PD-1-treated 4T1-based model (n = 6 for the chitin + 25 µg anti-PD-1 group and n = 8 for all other groups at all time points). (B) In vivo imaging of primary tumor bioluminescent signals (total flux density in p/s/cm²) in the untreated, 25 µg IgG control-, 25 µg anti-PD-1-, chitin- and chitin + 25 µg anti-PD-1-treated 4T1-based model (n = 6 for the chitin + 25 µg anti-PD-1 group and n = 8 for all other groups at all time points). (C) Representative images of primary tumor bioluminescence in the untreated and treated 4T1-based model at 5 w p.i. (D,E) Quantification of bioluminescent signals (total flux density in p/s/cm²) in lungs (D) and representative lung images from the untreated and treated 4T1-based model at 5 w p.i. (E) (n = 3 for the chitin + 25 µg anti-PD-1 group and n = 4 for all other groups). (F) H&E histology of lung metastases from the untreated and treated 4T1-based model at 5 w p.i. Dashed inserts highlight H&E-stained metastases at a larger magnification. Black scale bars = 200 µm, red scale bars = 50 μm. (G) Spleen weight measurements from the untreated and treated 4T1-based model at 5 w p.i. (n = 3 for the chitin + 25 µg anti-PD-1 group and n = 4 for all other groups). Data are presented as the means +/- SEM. *: P < 0.05, **: P < 0.01.

**Additional file 6: Figure S4.** Chitin reduces CHI3L4 production but not statistically significantly in 4T1 and 66cl4 primary tumors. (A) Representative western blot images for CHI3L3 + CHI3L4 and GAPDH loading control in primary tumor lysates from untreated, anti-PD-1-, chitin- and chitin + anti-PD-1-treated 4T1 and 66cl4 tumor-bearing mice at 5 w p.i. (B) Quantification of CHI3L3 + CHI3L4 signal intensity relative to GAPDH (n = 6 for all groups; 3 western blots with 2 samples from each group per blot). (C) CHI3L4 relative signal intensity based on subtraction of the CHI3L3 signal (as shown in Fig. 3A) from the CHI3L3 + CHI3L4 combined signal (n = 6 for all groups). Data are presented as the means +/- SEM. *: P < 0.05, **: P < 0.01.

**Additional file 7: Figure S5.** Immunohistochemistry confirms reduction of CHI3L1 levels in 4T1 and CHI3L3 levels in both 4T1 and 66cl4 primary tumors following chitin treatment, with concomitant reduction of Ly6G+ TANs. (A-C) Immunohistochemistry for CHI3L1 (A), CHI3L3 (B) and the PMN-MDSC/TAN marker Ly6G (C) on primary tumor sections from untreated, anti-PD-1-, chitin- and chitin + anti-PD-1-treated 4T1 and 66cl4 tumor-bearing mice at 5 w p.i. (n = 16 for all groups; 4 slides with 4 images per slide). Dashed inserts highlight stained tissue at a larger magnification. Black scale bars = 200 µm, red scale bars = 50 μm. Data are presented as the means +/- SEM. *: P < 0.05, ***: P < 0.001.

**Additional file 8: Figure S6.** Gating strategy for the flow cytometric immunophenotyping of myeloid cell types. (A) Gating of CD45+ leukocytes after excluding doublets and debris, also applied in the panels prior to gating of specific immune cell types. (B) Gating of CD45+ CD11b+ myeloid cells for subsequent gating of CD45+ CD11b+ CD14+ and CD45+ CD11b+ CD14- myeloid cells. The CD45+ CD11b+ CD14+ myeloid cells were further subdivided into CD45+ CD11b+ CD14+ Ly6Cint Ly6G+ PMN-MDSCs and CD45+ CD11b+ CD14+ Ly6Chi Ly6G- M-MDSCs, and the CD45+ CD11b+ CD14- myeloid cells were further gated towards CD45+ CD11b+ CD14- Ly6Cint Ly6G+ neutrophils/TANs. (C) Gating of CD45+ CD11b+ myeloid cells for subsequent gating of CD45+ CD11b+ CD14+ myeloid cells and CD45+ CD11b+ CD14+ F4/80+ macrophages/TAMs. Applying CD80 and MHCII allowed additional gating of CD45+ CD11b+ CD14+ F4/80+ CD80+ MHCII+ M1 macrophage/TAM subtypes and applying CD206 allowed gating of CD45+ CD11b+ CD14+ F4/80+ CD206+ M2 macrophage/TAM subtypes. (D) Gating of CD45+ CD11b+ myeloid cells for subsequent gating of CD45+ CD11b+ CD11c+ DCs.

**Additional file 9: Figure S7. **Gating strategy for the flow cytometric immunophenotyping of lymphocytic cell types. (A) Gating of CD45+ CD3ε+ T-cells for subsequent gating of CD45+ CD3ε+ CD4+ CD8α- and CD45+ CD3ε+ CD4- CD8α+ T-cell subtypes. (B) Gating of CD45+ CD3ε- cells for subsequent gating of CD45+ CD3ε- NKp46- cells and CD45+ CD3ε- NKp46- CD19+ B220+ B-cells. (C) Gating of CD45+ CD3ε+ T-cells for subsequent gating of CD45+ CD3ε+ NKp46+ NK-T cells. Gating of CD45+ CD3ε- cells was also applied for subsequent gating of CD45+ CD3ε- NKp46+ NK cells.

**Additional file 10: Figure S8**. Flow cytometric data from figure 5 shown as % of CD45+ cells. (A,B) Percentage of myeloid subpopulations (including PMN-MDSCs, M-MDSCs, TANs, TAMs and DCs) within the CD45+ leukocyte population of untreated and treated 4T1 (A) and 66cl4 primary tumors (B). (C,D) Percentage of M1 and M2 TAM subtypes within the CD45+ leukocyte population of untreated and treated 4T1 (C) and 66cl4 primary tumors (D). (E,F) Percentage of lymphocytic subpopulations (including CD4+ and CD8α+ T-cells, B-cells, NK cells and NK-T cells) within the CD45+ leukocyte population of untreated and treated 4T1 (E) and 66cl4 primary tumors (F). Data are presented as the means +/- SEM with n =5 for all groups. *: P < 0.05, **: P < 0.01, ***: P < 0.001.

** Additional file 11: Figure S9.** Immunohistochemistry confirms the M2 TAM reduction and enhanced lymphocytic activation upon chitin either with or without anti-PD-1 treatment in 4T1 and 66cl4 primary tumors. (A,B) Immunohistochemistry for the M2 TAM subtype marker CD163 (A) and the lymphocytic activation marker granzyme B (B) on primary tumor sections from untreated, anti-PD-1-, chitin- and chitin + anti-PD-1-treated 4T1 and 66cl4 tumor-bearing mice at 5 w p.i. (n = 16 for all groups; 4 slides with 4 images per slide). Dashed inserts highlight stained tissue at a larger magnification. Black scale bars = 200 µm, red scale bars = 50 μm. Data are presented as the means +/- SEM. *: P < 0.05, **: P < 0.01, ***: P < 0.001.

**Additional file 12: Figure S10.** Six of the investigated cytokines remain unchanged upon chitin with or without anti-PD-1 treatment in 4T1 and 66cl4 primary tumors. (A,B) Levels for G-CSF, IL-1β, IL-4, IL-6, IL-10 and TGF-β1 in primary tumor lysates from untreated, anti-PD-1-, chitin- and chitin + anti-PD-1-treated 4T1 (A) and 66cl4 tumor-bearing mice (B) at 5 w p.i. (n = 5 for all groups in the 4T1-based model; n = 11 for all groups in the 66cl4-based model). Data are presented as the means +/- SEM.

**Additional file 13: Figure S11.** Inflammation-related gene expression levels corroborate the reduced immunosuppressive activity upon chitin treatment in 4T1 and 66cl4 primary tumors. (A,B) Heatmaps showing normalized expression levels of selected genes associated with inflammation in 4T1 (A) and 66cl4 primary tumors (B) at 5 w p.i. derived from untreated, anti-PD-1, chitin- and chitin + anti-PD-1-treated tumor-bearing mice. Mean normalized expression levels were calculated based on normalized expression levels in 4 or 5 primary tumor samples from each treatment group and for each model. Selection of the genes was based on gene lists from the NanoString Mouse PanCancer Immune Profiling Panel. Pearson distance was used for hierarchical clustering.

**Additional file 14: Figure S12.** Flow cytometric data from figure 7 shown as % of CD45+ cells. (A,B) Percentage of myeloid subpopulations (including PMN-MDSCs, M-MDSCs, neutrophils, macrophages and DCs) within the CD45+ leukocyte population of axillary lymph nodes derived from the untreated and treated 4T1- (A) and 66cl4-based model (B). (C,D) Percentage of M1 and M2 macrophage subtypes within the CD45+ leukocyte population of axillary lymph nodes derived from the untreated and treated 4T1- (C) and 66cl4-based model (D). (E,F) Percentage of lymphocytic subpopulations (including CD4+ and CD8α+ T-cells, B-cells, NK cells and NK-T cells) within the CD45+ leukocyte population of axillary lymph nodes derived from the untreated and treated 4T1- (E) and 66cl4-based model (F). Data are presented as the means +/- SEM with n = 5 for all groups. *: P < 0.05, **: P < 0.01, ***: P < 0.001.

**Additional file 15: Figure S13.** Flow cytometric data from figure 8 shown as % of CD45+ cells. (A,B) Percentage of myeloid subpopulations (including PMN-MDSCs, M-MDSCs, neutrophils, macrophages and DCs) within the CD45+ leukocyte population of spleens derived from the untreated and treated 4T1- (A) and 66cl4-based model (B). (C,D) Percentage of M1 and M2 macrophage subtypes within the CD45+ leukocyte population of spleens derived from the untreated and treated 4T1- (C) and 66cl4-based model (D). (E,F) Percentage of lymphocytic subpopulations (including CD4+ and CD8α+ T-cells, B-cells, NK cells and NK-T cells) within the CD45+ leukocyte population of spleens derived from the untreated and treated 4T1- (E) and 66cl4-based model (F). Data are presented as the means +/- SEM with n = 5 for all groups. *: P < 0.05, **: P < 0.01, ***: P < 0.001.

**Additional file 16: Figure S14.** Chitin reduces immunosuppression and increases CD8α+ T-cells and their activation to a higher extent than anti-CHI3L1 treatment in the 4T1-based intraductal model. (A-F) Primary tumors were isolated from the IgG control-, anti-CHI3L1- and chitin-treated 4T1-based model at 5 w p.i. and processed into a single cell suspension for flow cytometric immunophenotyping (n = 3 for the IgG control and anti-CHI3L1 group, n = 5 for the chitin group). (A,B) Number of myeloid subpopulations (including PMN-MDSCs, M-MDSCs and TANs) per gram of untreated and treated primary tumor (A) and percentage of these myeloid subpopulations within the CD45+ leukocyte population of untreated and treated primary tumors (B). (C) Calculated M1/M2 TAM ratio in untreated and treated primary tumors. (D,E) Number of CD8α+ T-cells per gram of untreated and treated primary tumor (D) and percentage of these CD8α+ T-cells within the CD45+ leukocyte population of untreated and treated primary tumors (E). (F) Percentage of granzyme B+, Ki67+, IFN-γ+ and PD-1+ cells within the primary tumor CD8α+ T-cell population in the untreated and treated 4T1-based model. (G) Percentage of FoxP3+ cells within the primary tumor CD4+ T-cell population in the untreated and treated 4T1-based model. Data are presented as the means +/- SEM. *: P < 0.05, **: P < 0.01, ***: P < 0.001.

**Additional file 17: Figure S15.** Publicly available data from BC patients correlate human CHI3L1 expression with hormonal and immune cell markers. (A,B) Amplification frequency of CHI3L1 in primary breast tumors according to the TCGA and METABRIC database (A), and metastatic BC according to a published INSERM study and the MBCproject database (B), both for all BC subtypes and TNBC. (C) Negative correlation between expression of CHI3L1 and hormone receptors ER, PR and HER2/ERBB2 based on the TCGA database. (D) Positive correlation between expression of CHI3L1 and myeloid cell markers ITGAM, CD14, MPO, CD68 and CD163 based on the TCGA database. (E) Positive correlation between expression of CHI3L1 and immune checkpoint marker PDCD1 as well as T-reg marker FOXP3 based on the TCGA database.

**Additional file 18: Figure S16.** Full western blot images supporting the cropped images used in figure 3A and the specificity of the used CHI3L1 antibody. Uncropped blot images for CHI3L1, CHI3L3 and GAPDH loading controls in primary tumor lysates from untreated, anti-PD-1-, chitin- and chitin + anti-PD-1-treated 4T1 and 66cl4 tumor-bearing mice at 5 w p.i., supporting the cropped images in figure 3A. CHI3L1 antibody specificity was validated by western blot, showing a signal at 43 kDa with increasing intensity starting from 1 ng to 100 ng dose of rmCHI3L1.

**Additional file 19: Figure S17.** Full western blot images supporting the cropped images used in figure S4A. Uncropped blot images for CHI3L3 + CHI3L4 and GAPDH loading control in primary tumor lysates from untreated, anti-PD-1-, chitin- and chitin + anti-PD-1-treated 4T1 and 66cl4 tumor-bearing mice at 5 w p.i., supporting the cropped images in figure S4A.

## Data Availability

All data generated or analysed during this study are included in this published article and its additional files. RNA-seq data were uploaded to GEO (Gene-Expression Omnibus, accession number GSE256439). Additional information is available from the corresponding author on reasonable request.

## Abbreviations

CLP: Chitinase-like protein
TNBC: Triple-negative breast cancer
PD-1: Programmed death-1
ICB: Immune checkpoint blockade
CHI3L1: Chitinase 3-like 1
CHI3L3: Chitinase 3-like 3
CHI3L4: Chitinase 3-like 4
SI-CLP: Stabilin-1 interacting chitinase-like protein
TME: Tumor microenvironment
PMN-MDSC: Polymorphonuclear-myeloid-derived suppressor cell
M-MDSC: Monocytic-myeloid-derived suppressor cell
TAN: Tumor-associated neutrophil
TAM: Tumor-associated macrophage
DC: Dendritic cell
T-reg: Regulatory T-cell
NK: Natural killer
i.p.: Intraperitoneal
IFN-γ: Interferon-γ
MCP-1: Monocyte chemoattractant protein-1
MIP-2: Macrophage inflammatory protein-2
G-CSF: Granulocyte-colony stimulating factor
IL: Interleukin
TGF-β1: Transforming growth factor-β1
TNF-α: Tumor necrosis factor-α
Stat3: Signal transducer and activator of transcription 3
NET: Neutrophil extracellular trap
MPO: Myeloperoxidase
FoxP3: Forkhead box P3
SEMA7A: Semaphorin 7A
PDPN: Podoplanin
LYVE-1: Lymphatic vessel endothelial hyaluronan receptor 1
EMH: Extramedullary hematopoiesis
CTLA-4: Cytotoxic T-lymphocyte antigen-4

## References

[1] Gupta GK, Collier AL, Lee D, Hoefer RA, Zheleva V, Siewertsz van Reesema LL, Tang-Tan AM, Guye ML, Chang DZ, Winston JS, Samli B, Jansen RJ, Petricoin EF, Goetz MP, Bear HD, Tang AH. Perspectives on triple-negative breast cancer: current treatment strategies, unmet needs, and potential targets for future therapies. Cancers Basel. 2020;12(9):2392. 10.3390/cancers12092392 PMC7565566 32846967

[2] Thomas R, Al-Khadairi G, Decock J (2021). Immune checkpoint inhibitors in triple negative breast cancer treatment: promising future prospects. Front Oncol.

[3] Ribeiro R, Carvalho MJ, Goncalves J, Moreira JN (2022). Immunotherapy in triple-negative breast cancer: insights into tumor immune landscape and therapeutic opportunities. Front Mol Biosci.

[4] Tie Y, Tang F, Wei YQ, Wei XW (2022). Immunosuppressive cells in cancer: mechanisms and potential therapeutic targets. J Hematol Oncol.

[5] Labani-Motlagh A, Ashja-Mahdavi M, Loskog A (2020). The tumor microenvironment: a milieu hindering and obstructing antitumor immune responses. Front Immunol.

[6] Lee CG, Da Silva CA, Dela Cruz CS, Ahangari F, Ma B, Kang MJ, He CH, Takyar S, Elias JA (2011). Role of chitin and chitinase/chitinase-like proteins in inflammation, tissue remodeling, and injury. Annu Rev Physiol.

[7] Sutherland TE (2018). Chitinase-like proteins as regulators of innate immunity and tissue repair: helpful lessons for asthma?. Biochem Soc Trans.

[8] Steenbrugge J, Breyne K, Denies S, Dekimpe M, Demeyere K, De Wever O, Vermeulen P, Van Laere S, Sanders NN, Meyer E (2016). Comparison of the adipose and luminal mammary gland compartment as orthotopic inoculation sites in a 4T1-based immunocompetent preclinical model for triple-negative breast cancer. J Mammary Gland Biol Neoplasia.

[9] Johansen JS, Jensen BV, Roslind A, Nielsen D, Price PA (2006). Serum YKL-40, a new prognostic biomarker in cancer patients?. Cancer Epidemiol Biomarkers Prev.

[10] Shao R, Cao QJ, Arenas RB, Bigelow C, Bentley B, Yan W (2011). Breast cancer expression of YKL-40 correlates with tumour grade, poor differentiation, and other cancer markers. Br J Cancer.

[11] Zhao T, Su Z, Li Y, Zhang X, You Q (2020). Chitinase-3 like-protein-1 function and its role in diseases. Signal Transduct Target Ther.

[12] Cohen N, Shani O, Raz Y, Sharon Y, Hoffman D, Abramovitz L, Erez N (2017). Fibroblasts drive an immunosuppressive and growth-promoting microenvironment in breast cancer via secretion of Chitinase 3-like 1. Oncogene.

[13] Chen Y, Zhang S, Wang Q, Zhang X (2017). Tumor-recruited M2 macrophages promote gastric and breast cancer metastasis via M2 macrophage-secreted CHI3L1 protein. J Hematol Oncol.

[14] Steenbrugge J, Breyne K, Demeyere K, De Wever O, Sanders NN, Van Den Broeck W, Colpaert C, Vermeulen P, Van Laere S, Meyer E (2018). Anti-inflammatory signaling by mammary tumor cells mediates prometastatic macrophage polarization in an innovative intraductal mouse model for triple-negative breast cancer. J Exp Clin Cancer Res.

[15] Kim DH, Park HJ, Lim S, Koo JH, Lee HG, Choi JO, Oh JH, Ha SJ, Kang MJ, Lee CM, Lee CG, Elias JA, Choi JM (2018). Regulation of chitinase-3-like-1 in T cell elicits Th1 and cytotoxic responses to inhibit lung metastasis. Nat Commun.

[16] Darwich A, Silvestri A, Benmebarek MR, Mouriès J, Cadilha B, Melacarne A, Morelli L, Supino D, Taleb A, Obeck H, Sustmann C, Losurdo A, Masci G, Curigliano G, Kobold S, Penna G, Rescigno M (2021). Paralysis of the cytotoxic granule machinery is a new cancer immune evasion mechanism mediated by chitinase 3-like-1. J Immunother Cancer.

[17] Perry CJ, Muñoz-Rojas AR, Meeth KM, Kellman LN, Amezquita RA, Thakral D, Du VY, Wang JX, Damsky W, Kuhlmann AL, Sher JW, Bosenberg M, Miller-Jensen K, Kaech SM (2018). Myeloid-targeted immunotherapies act in synergy to induce inflammation and antitumor immunity. J Exp Med.

[18] Fleming Martinez AK, Döppler HR, Bastea LI, Edenfield BH, Liou GY, Storz P. Ym1+ macrophages orchestrate fibrosis, lesion growth, and progression during development of murine pancreatic cancer. iScience. 2022;25(5):104327. 10.1016/j.isci.2022.104327 PMC9118688 35602933

[19] Funkhouser JD, Aronson NN (2007). Chitinase family GH18: evolutionary insights from the genomic history of a diverse protein family. BMC Evol Biol.

[20] Libreros S, Garcia-Areas R, Shibata Y, Carrio R, Torroella-Kouri M, Iragavarapu-Charyulu V (2012). Induction of proinflammatory mediators by CHI3L1 is reduced by chitin treatment: decreased tumor metastasis in a breast cancer model. Int J Cancer.

[21] Libreros S, Garcia-Areas R, Keating P, Carrio R, Iragavarapu-Charyulu VL (2013). Exploring the role of CHI3L1 in "pre-metastatic" lungs of mammary tumor-bearing mice. Front Physiol.

[22] Steenbrugge J, Bellemans J, Vander Elst N, Demeyere K, De Vliegher J, Perera T, De Wever O, Van Den Broeck W, De Spiegelaere W, Sanders NN, Meyer E (2022). One cisplatin dose provides durable stimulation of anti-tumor immunity and alleviates anti-PD-1 resistance in an intraductal model for triple-negative breast cancer. Oncoimmunology.

[23] Breyne K, Steenbrugge J, Demeyere K, Lee CG, Elias JA, Petzl W, Smith DGE, Germon P, Meyer E (2018). Immunomodulation of host chitinase 3-like 1 during a mammary pathogenic *Escherichia coli* infection. Front Immunol.

[24] Zhang X, Mao Y, Briber RM (2022). Efficient production of oligomeric chitin with narrow distributions of degree of polymerization using sonication-assisted phosphoric acid hydrolysis. Carbohydr Polym.

[25] Steenbrugge J, Vander Elst N, Demeyere K, De Wever O, Sanders NN, Van Den Broeck W, Dirix L, Van Laere S, Meyer E (2019). Comparative profiling of metastatic 4T1- vs. non-metastatic Py230-based mammary tumors in an intraductal model for triple-negative breast cancer. Front Immunol..

[26] Qiu QC, Wang L, Jin SS, Liu GF, Liu J, Ma L, Mao RF, Ma YY, Zhao N, Chen M, Lin BY (2018). CHI3L1 promotes tumor progression by activating TGF-β signaling pathway in hepatocellular carcinoma. Sci Rep.

[27] Xu W, Chao R, Xie X, Mao Y, Chen X, Chen X, Zhang S (2024). IL13Rα2 as a crucial receptor for Chi3l1 in osteoclast differentiation and bone resorption through the MAPK/AKT pathway. Cell Commun Signal.

[28] Elder AM, Tamburini BAJ, Crump LS, Black SA, Wessells VM, Schedin PJ, Borges VF, Lyons TR (2018). Semaphorin 7A promotes macrophage-mediated lymphatic remodeling during postpartum mammary gland involution and in breast cancer. Cancer Res.

[29] Babicki S, Arndt D, Marcu A, Liang Y, Grant JR, Maciejewski A, Wishart DS (2016). Heatmapper: web-enabled heat mapping for all. Nucleic Acids Res.

[30] DuPre' SA, Hunter KW (2007). Murine mammary carcinoma 4T1 induces a leukemoid reaction with splenomegaly: association with tumor-derived growth factors. Exp Mol Pathol.

[31] Singh SK, Bhardwaj R, Wilczynska KM, Dumur CI, Kordula T (2011). A complex of nuclear factor I-X3 and STAT3 regulates astrocyte and glioma migration through the secreted glycoprotein YKL-40. J Biol Chem.

[32] Tran HT, Lee IA, Low D, Kamba A, Mizoguchi A, Shi HN, Lee CG, Elias JA, Mizoguchi E (2014). Chitinase 3-like 1 synergistically activates IL6-mediated STAT3 phosphorylation in intestinal epithelial cells in murine models of infectious colitis. Inflamm Bowel Dis.

[33] Yan C, Ding X, Wu L, Yu M, Qu P, Du H (2013). Stat3 downstream gene product chitinase 3-like 1 is a potential biomarker of inflammation-induced lung cancer in multiple mouse lung tumor models and humans. PLoS ONE.

[34] Kim KC, Yun J, Son DJ, Kim JY, Jung JK, Choi JS, Kim YR, Song JK, Kim SY, Kang SK, Shin DH, Roh YS, Han SB, Hong JT (2018). Suppression of metastasis through inhibition of chitinase 3-like 1 expression by miR-125a-3p-mediated up-regulation of USF1. Theranostics.

[35] Yang PS, Yu MH, Hou YC, Chang CP, Lin SC, Kuo IY, Su PC, Cheng HC, Su WC, Shan YS, Wang YC (2022). Targeting protumor factor chitinase-3-like-1 secreted by Rab37 vesicles for cancer immunotherapy. Theranostics.

[36] Chen A, Jiang Y, Li Z, Wu L, Santiago U, Zou H, Cai C, Sharma V, Guan Y, McCarl LH, Ma J, Wu YL, Michel J, Shi Y, Konnikova L, Amankulor NM, Zinn PO, Kohanbash G, Agnihotri S, Lu S, Lu X, Sun D, Gittes GK, Wang Q, Xiao X, Yimlamai D, Pollack IF, Camacho CJ, Hu B (2021). Chitinase-3-like 1 protein complexes modulate macrophage-mediated immune suppression in glioblastoma. J Clin Invest.

[37] Győrffy B (2021). Survival analysis across the entire transcriptome identifies biomarkers with the highest prognostic power in breast cancer. Comput Struct Biotechnol J.

[38] Ma B, Herzog EL, Lee CG, Peng X, Lee CM, Chen X, Rockwell S, Koo JS, Kluger H, Herbst RS, Sznol M, Elias JA (2015). Role of chitinase 3-like-1 and semaphorin 7a in pulmonary melanoma metastasis. Cancer Res.

[39] Ma B, Akosman B, Kamle S, Lee CM, He CH, Koo JS, Lee CG, Elias JA (2021). CHI3L1 regulates PD-L1 and anti-CHI3L1-PD-1 antibody elicits synergistic antitumor responses. J Clin Invest.

[40] Ma B, Kamle S, Akosman B, Khan H, Lee CM, Lee CG, Elias JA (2022). CHI3L1 enhances melanoma lung metastasis via regulation of T cell co-stimulators and CTLA-4/B7 axis. Front Immunol.

[41] Guetta-Terrier C, Karambizi D, Akosman B, Zepecki JP, Chen JS, Kamle S, Fajardo JE, Fiser A, Singh R, Toms SA, Lee CG, Elias JA, Tapinos N (2023). Chi3l1 Is a modulator of glioma stem cell states and a therapeutic target in glioblastoma. Cancer Res.

[42] Yu JE, Yeo IJ, Son DJ, Yun J, Han SB, Hong JT (2022). Anti-Chi3L1 antibody suppresses lung tumor growth and metastasis through inhibition of M2 polarization. Mol Oncol.

[43] Behbod F, Kittrell FS, LaMarca H, Edwards D, Kerbawy S, Heestand JC, Young E, Mukhopadhyay P, Yeh HW, Allred DC, Hu M, Polyak K, Rosen JM, Medina D (2009). An intraductal human-in-mouse transplantation model mimics the subtypes of ductal carcinoma in situ. Breast Cancer Res.

[44] Ghosh A, Sarkar S, Banerjee S, Behbod F, Tawfik O, McGregor D, Graff S, Banerjee SK (2018). MIND model for triple-negative breast cancer in syngeneic mice for quick and sequential progression analysis of lung metastasis. PLoS ONE.

[45] Luo XL, Lin L, Hu H, Hu FL, Lin Y, Luo ML, Wang L, He YQ (2020). Development and characterization of mammary intraductal (MIND) spontaneous metastasis models for triple-negative breast cancer in syngeneic mice. Sci Rep.

[46] Hughes K, Wickenden JA, Allen JE, Watson CJ (2012). Conditional deletion of Stat3 in mammary epithelium impairs the acute phase response and modulates immune cell numbers during post-lactational regression. J Pathol.

[47] Allouch S, Gupta I, Malik S, Al Farsi HF, Vranic S, Al Moustafa AE (2020). Breast cancer during pregnancy: a marked propensity to triple-negative phenotype. Front Oncol.

[48] Volck B, Price PA, Johansen JS, Sørensen O, Benfield TL, Nielsen HJ, Calafat J, Borregaard N (1998). YKL-40, a mammalian member of the chitinase family, is a matrix protein of specific granules in human neutrophils. Proc Assoc Am Physicians.

[49] Coriati A, Massé C, Ménard A, Bouvet GF, Berthiaume Y (2018). Neutrophils as a potential source of chitinase-3-like protein 1 in cystic fibrosis. Inflammation.

[50] Deutschmann C, Sowa M, Murugaiyan J, Roesler U, Röber N, Conrad K, Laass MW, Bogdanos D, Sipeki N, Papp M, Rödiger S, Roggenbuck D, Schierack P (2019). Identification of chitinase-3-like protein 1 as a novel neutrophil antigenic target in Crohn's disease. J Crohns Colitis.

[51] Taifour T, Attalla SS, Zuo D, Gu Y, Sanguin-Gendreau V, Proud H, Solymoss E, Bui T, Kuasne H, Papavasiliou V, Lee CG, Kamle S, Siegel PM, Elias JA, Park M, Muller WJ (2023). The tumor-derived cytokine Chi3l1 induces neutrophil extracellular traps that promote T cell exclusion in triple-negative breast cancer. Immunity.

[52] Kumar S, Wilkes DW, Samuel N, Blanco MA, Nayak A, Alicea-Torres K, Gluck C, Sinha S, Gabrilovich D, Chakrabarti R (2018). ΔNp63-driven recruitment of myeloid-derived suppressor cells promotes metastasis in triple-negative breast cancer. J Clin Invest.

[53] He CH, Lee CG, Dela Cruz CS, Lee CM, Zhou Y, Ahangari F, Ma B, Herzog EL, Rosenberg SA, Li Y, Nour AM, Parikh CR, Schmidt I, Modis Y, Cantley L, Elias JA (2013). Chitinase 3-like 1 regulates cellular and tissue responses via IL-13 receptor α2. Cell Rep.

[54] Raskov H, Orhan A, Gaggar S, Gögenur I (2022). Neutrophils and polymorphonuclear myeloid-derived suppressor cells: an emerging battleground in cancer therapy. Oncogenesis.

[55] Veglia F, Hashimoto A, Dweep H, Sanseviero E, De Leo A, Tcyganov E, Kossenkov A, Mulligan C, Nam B, Masters G, Patel J, Bhargava V, Wilkinson P, Smirnov D, Sepulveda MA, Singhal S, Eruslanov EB, Cristescu R, Loboda A, Nefedova Y, Gabrilovich DI (2021). Analysis of classical neutrophils and polymorphonuclear myeloid-derived suppressor cells in cancer patients and tumor-bearing mice. J Exp Med.

[56] Kamada T, Togashi Y, Tay C, Ha D, Sasaki A, Nakamura Y, Sato E, Fukuoka S, Tada Y, Tanaka A, Morikawa H, Kawazoe A, Kinoshita T, Shitara K, Sakaguchi S, Nishikawa H (2019). PD-1+ regulatory T cells amplified by PD-1 blockade promote hyperprogression of cancer. Proc Natl Acad Sci U S A.

[57] Blomberg OS, Kos K, Spagnuolo L, Isaeva OI, Garner H, Wellenstein MD, Bakker N, Duits DEM, Kersten K, Klarenbeek S, Hau CS, Kaldenbach D, Raeven EAM, Vrijland K, Kok M, de Visser KE (2023). Neoadjuvant immune checkpoint blockade triggers persistent and systemic Treg activation which blunts therapeutic efficacy against metastatic spread of breast tumors. Oncoimmunology.

[58] Kawada M, Seno H, Kanda K, Nakanishi Y, Akitake R, Komekado H, Kawada K, Sakai Y, Mizoguchi E, Chiba T (2012). Chitinase 3-like 1 promotes macrophage recruitment and angiogenesis in colorectal cancer. Oncogene.

[59] Lin HW, Chiang YC, Sun NY, Chen YL, Chang CF, Tai YJ, Chen CA, Cheng WF (2019). CHI3L1 results in poor outcome of ovarian cancer by promoting properties of stem-like cells. Endocr Relat Cancer.

[60] Ji S, Yu H, Zhou D, Fan X, Duan Y, Tan Y, Lang M, Shao G (2023). Cancer stem cell-derived CHI3L1 activates the MAF/CTLA4 signaling pathway to promote immune escape in triple-negative breast cancer. J Transl Med.

[61] Steenbrugge J, De Jaeghere EA, Meyer E, Denys H, De Wever O (2021). Splenic hematopoietic and stromal cells in cancer progression. Cancer Res.

[62] Liu M, Jin X, He X, Pan L, Zhang X, Zhao Y (2015). Macrophages support splenic erythropoiesis in 4T1 tumor-bearing mice. PLoS ONE.

[63] Ravindranathan S, Nguyen KG, Kurtz SL, Frazier HN, Smith SG, Koppolu BP, Rajaram N, Zaharoff DA (2018). Tumor-derived granulocyte colony-stimulating factor diminishes efficacy of breast tumor cell vaccines. Breast Cancer Res.

[64] Pande K, Ueda R, Machemer T, Sathe M, Tsai V, Brin E, Delano MJ, Van Rooijen N, McClanahan TK, Talmadge JE, Moldawer LL, Phillips JH, LaFace DM (2009). Cancer-induced expansion and activation of CD11b+ Gr-1+ cells predispose mice to adenoviral-triggered anaphylactoid-type reactions. Mol Ther.

[65] Shan Z, Li L, Atkins CL, Wang M, Wen Y, Jeong J, Moreno NF, Feng D, Gui X, Zhang N, Lee CG, Elias JA, Lee WM, Gao B, Lam FW, An Z, Ju C (2021). Chitinase 3-like-1 contributes to acetaminophen-induced liver injury by promoting hepatic platelet recruitment. Elife.

[66] Azuma K, Osaki T, Minami S, Okamoto Y (2015). Anticancer and anti-inflammatory properties of chitin and chitosan oligosaccharides. J Funct Biomater.

[67] Baharlouei P, Rahman A (2022). Chitin and chitosan: prospective biomedical applications in drug delivery, cancer treatment, and wound healing. Mar Drugs.

[68] Shibata Y, Metzger WJ, Myrvik QN (1997). Chitin particle-induced cell-mediated immunity is inhibited by soluble mannan: mannose receptor-mediated phagocytosis initiates IL-12 production. J Immunol.

[69] Davis S, Cirone AM, Menzie J, Russell F, Dorey CK, Shibata Y, Wei J, Nan C (2018). Phagocytosis-mediated M1 activation by chitin but not by chitosan. Am J Physiol Cell Physiol.

