# Supplementary figures and images for: Chitin-mediated blockade of chitinase-like proteins reduces tumor immunosuppression, inhibits lymphatic metastasis and enhances anti-PD-1 efficacy in complementary TNBC models
Source: Breast Cancer Res. 2024 Apr 11;26:63. doi: 10.1186/s13058-024-01815-8 (PMC11007917; doi:10.1186/s13058-024-01815-8)

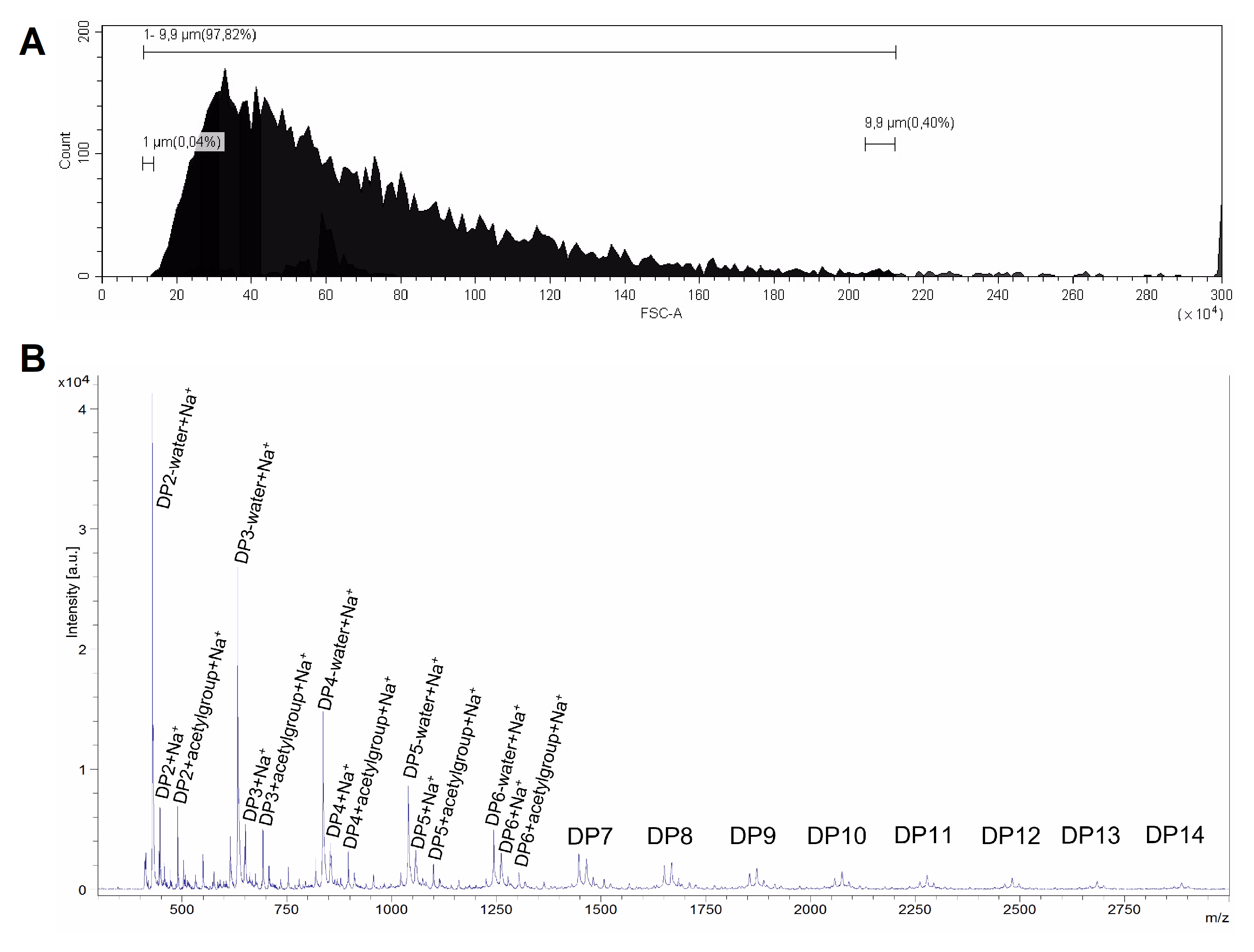

Supplement: Supplementary file 1 — Additional file 1: Figure S1. Validation of chitin particle size and acetylation. (A) Flow cytometric analysis of particle sizes in the chitin suspension for in vivo use with 1 and 9.9 µm microspheres indicated for size reference. (B) MALDI-TOF MS verifies the degree of polymerization and acetylation of the chitin suspension as previously reported [24]. [file 13058_2024_1815_MOESM1_ESM.tif]

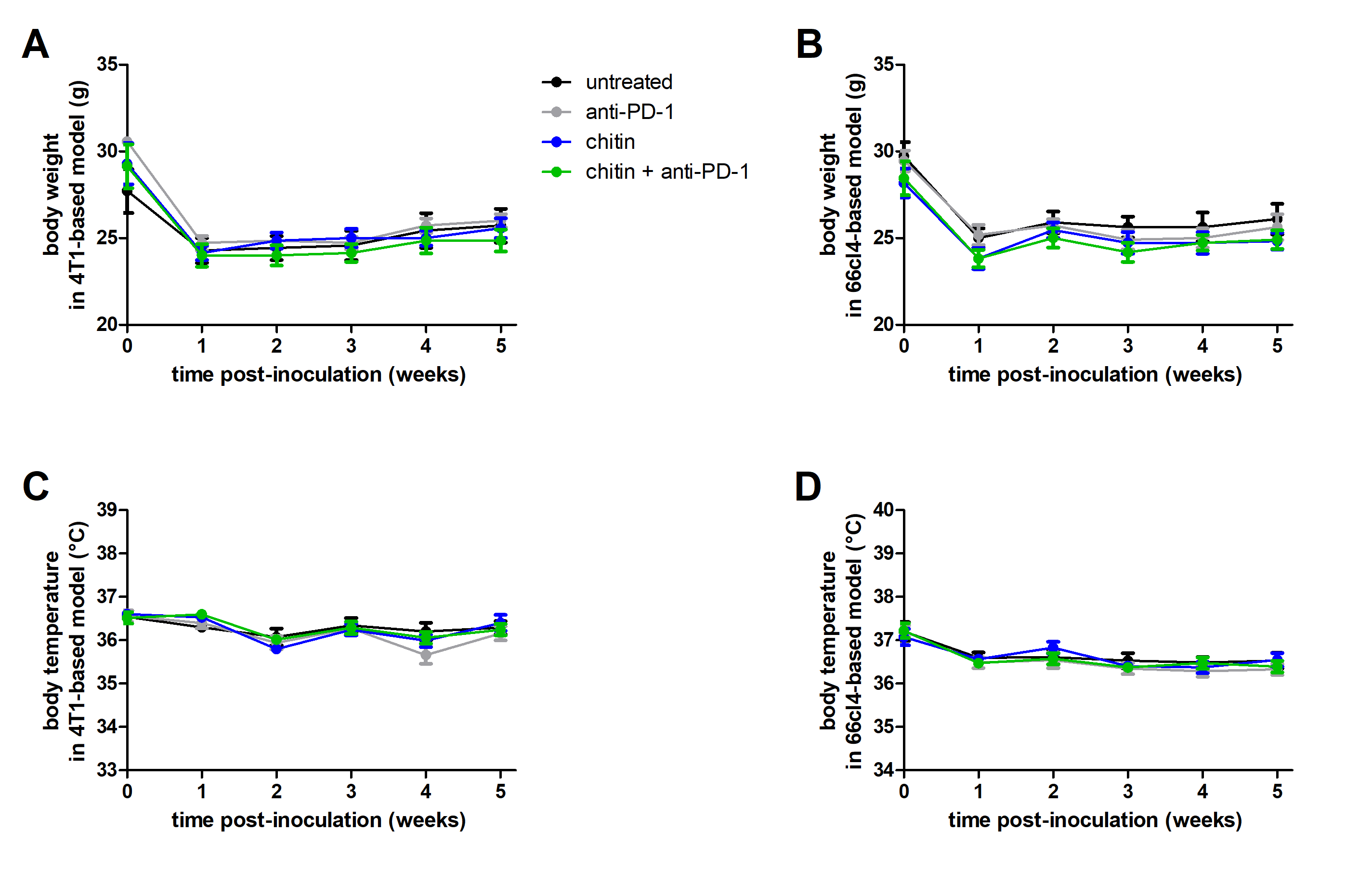

Supplement: Supplementary file 4 — Additional file 4: Figure S2. Chitin in combination with and without anti-PD-1 treatment has no toxic effect in a 4T1- and 66cl4-based intraductal model. (A,B) Weekly body weight measurements for animal welfare monitoring of the untreated and treated 4T1- (A) and 66cl4-based model (B) (n = 7 for all groups at all time points in the 4T1-based model; n = 11 for all groups at all time points in the 66cl4-based model). (C,D) Weekly body temperature measurements for animal welfare monitoring of the untreated and treated 4T1- (C) and 66cl4-based model (D) (n = 7 for all groups at all time points in the 4T1-based model; n = 11 for all groups at all time points in the 66cl4-based model). The decrease in body weight in both intraductal models during the first w p.i. can be attributed to cessation of milk production after pup weaning. Data are presented as the means +/- SEM. [file 13058_2024_1815_MOESM4_ESM.tif]

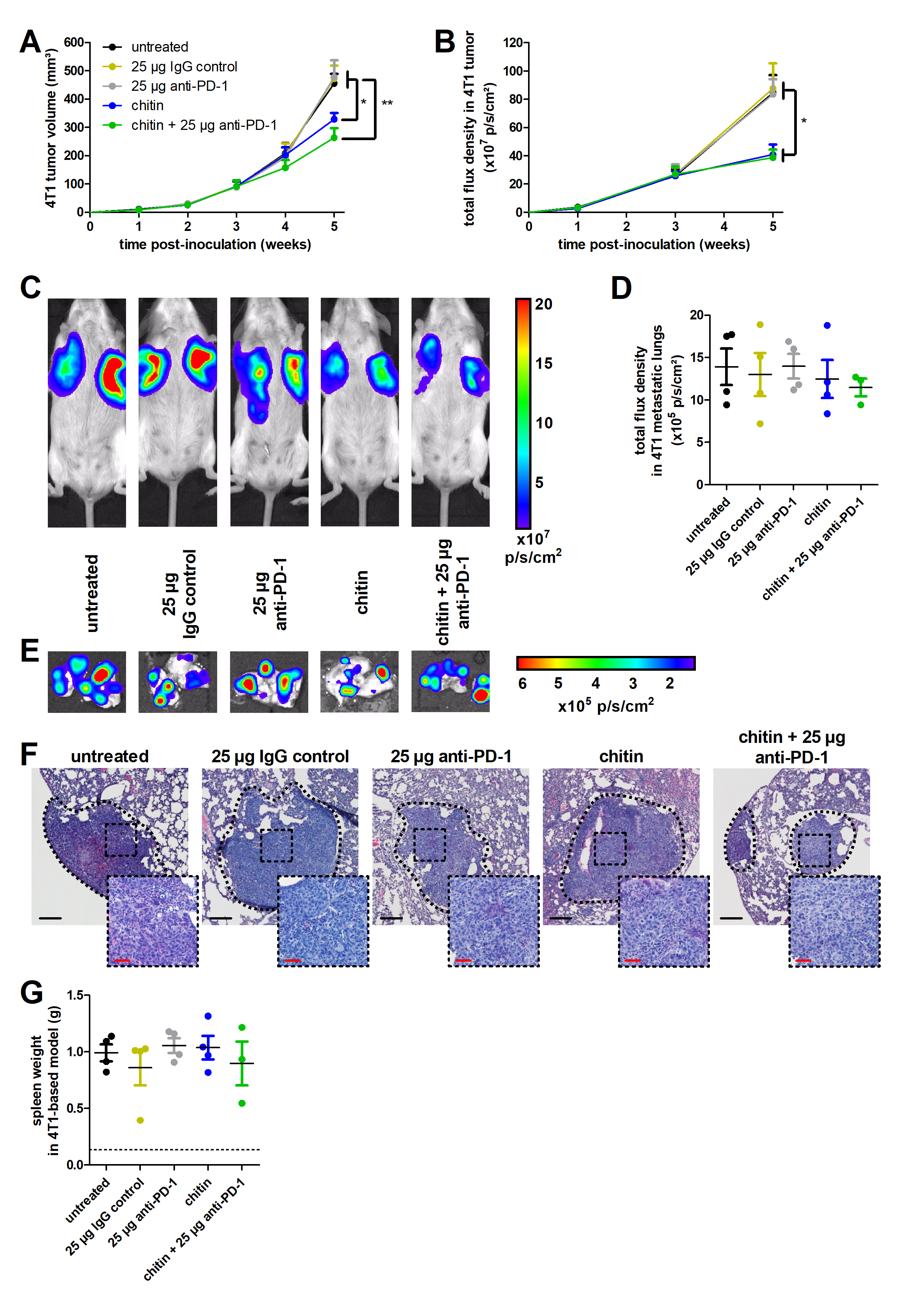

Supplement: Supplementary file 5 — Additional file 5: Figure S3. The treatment effect of a chitin and anti-PD-1 combination is anti-PD-1 dose-dependent in a 4T1-based intraductal model. (A) Weekly measurements of primary tumor volumes in the untreated, 25 µg IgG control-, 25 µg anti-PD-1-, chitin- and chitin + 25 µg anti-PD-1-treated 4T1-based model (n = 6 for the chitin + 25 µg anti-PD-1 group and n = 8 for all other groups at all time points). (B) In vivo imaging of primary tumor bioluminescent signals (total flux density in p/s/cm²) in the untreated, 25 µg IgG control-, 25 µg anti-PD-1-, chitin- and chitin + 25 µg anti-PD-1-treated 4T1-based model (n = 6 for the chitin + 25 µg anti-PD-1 group and n = 8 for all other groups at all time points). (C) Representative images of primary tumor bioluminescence in the untreated and treated 4T1-based model at 5 w p.i. (D,E) Quantification of bioluminescent signals (total flux density in p/s/cm²) in lungs (D) and representative lung images from the untreated and treated 4T1-based model at 5 w p.i. (E) (n = 3 for the chitin + 25 µg anti-PD-1 group and n = 4 for all other groups). (F) H&E histology of lung metastases from the untreated and treated 4T1-based model at 5 w p.i. Dashed inserts highlight H&E-stained metastases at a larger magnification. Black scale bars = 200 µm, red scale bars = 50 μm. (G) Spleen weight measurements from the untreated and treated 4T1-based model at 5 w p.i. (n = 3 for the chitin + 25 µg anti-PD-1 group and n = 4 for all other groups). Data are presented as the means +/- SEM. *: P < 0.05, **: P < 0.01. [file 13058_2024_1815_MOESM5_ESM.tif]

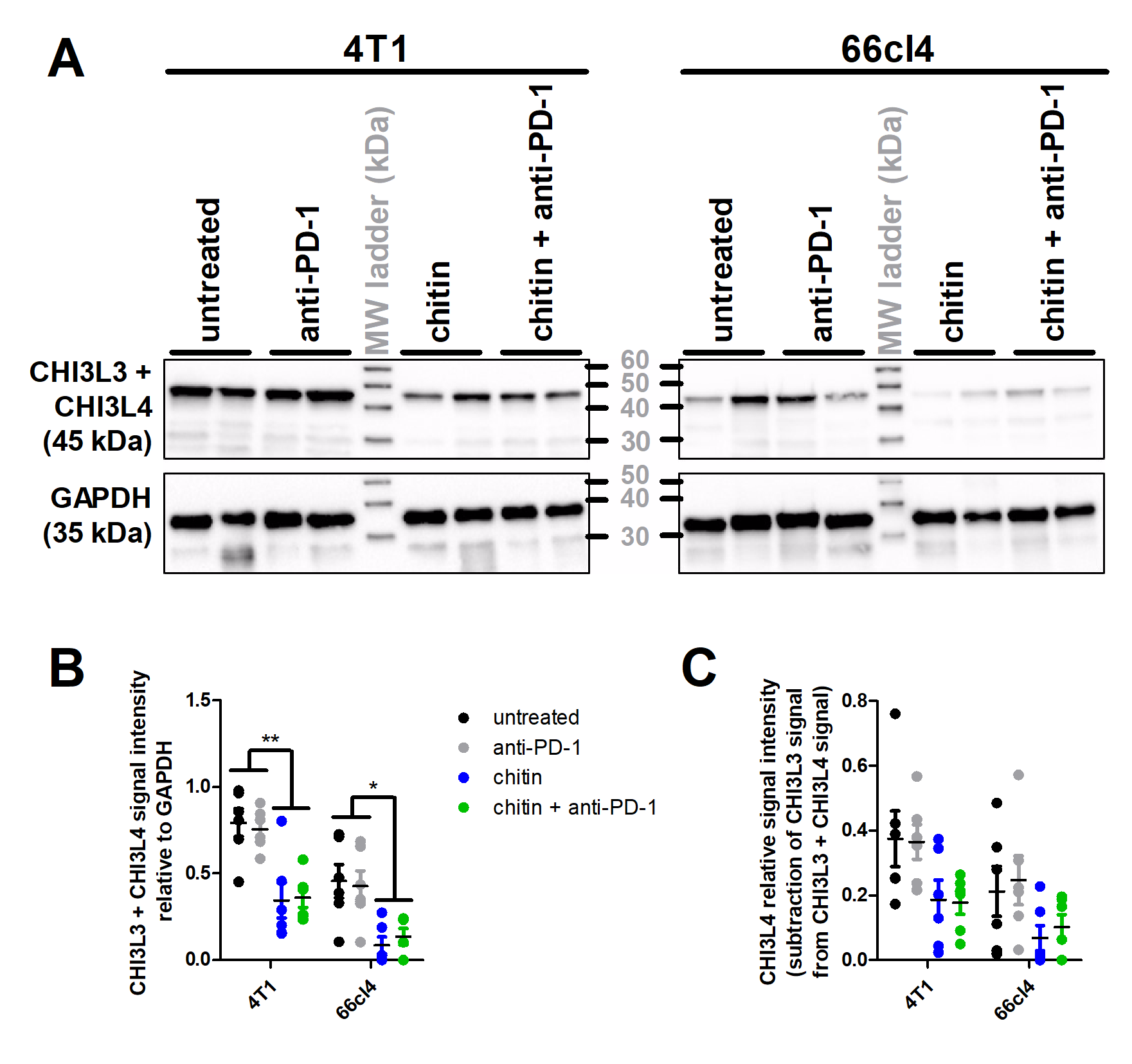

Supplement: Supplementary file 6 — Additional file 6: Figure S4. Chitin reduces CHI3L4 production but not statistically significantly in 4T1 and 66cl4 primary tumors. (A) Representative western blot images for CHI3L3 + CHI3L4 and GAPDH loading control in primary tumor lysates from untreated, anti-PD-1-, chitin- and chitin + anti-PD-1-treated 4T1 and 66cl4 tumor-bearing mice at 5 w p.i. (B) Quantification of CHI3L3 + CHI3L4 signal intensity relative to GAPDH (n = 6 for all groups; 3 western blots with 2 samples from each group per blot). (C) CHI3L4 relative signal intensity based on subtraction of the CHI3L3 signal (as shown in Fig. 3A) from the CHI3L3 + CHI3L4 combined signal (n = 6 for all groups). Data are presented as the means +/- SEM. *: P < 0.05, **: P < 0.01. [file 13058_2024_1815_MOESM6_ESM.tif]

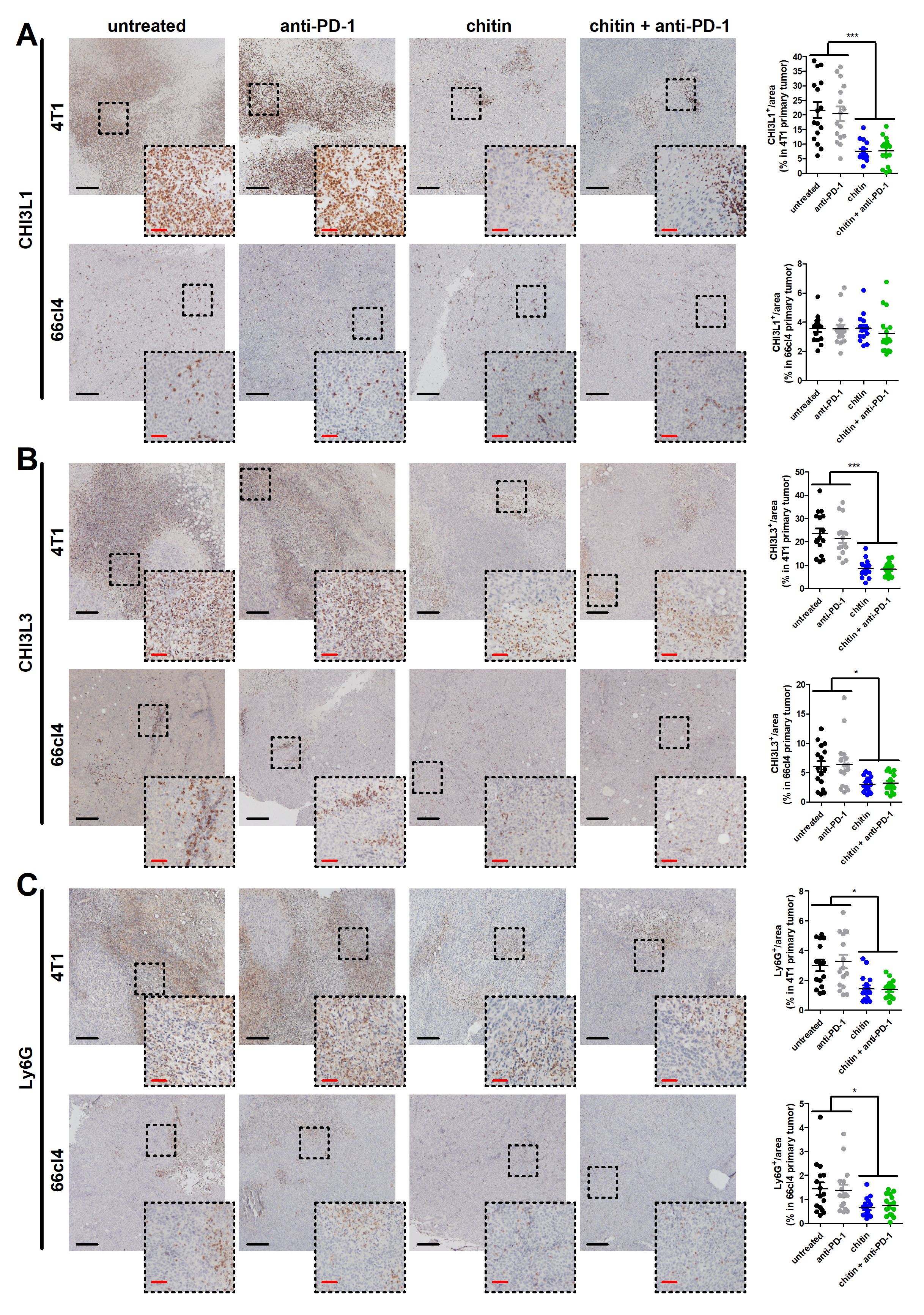

Supplement: Supplementary file 7 — Additional file 7: Figure S5. Immunohistochemistry confirms reduction of CHI3L1 levels in 4T1 and CHI3L3 levels in both 4T1 and 66cl4 primary tumors following chitin treatment, with concomitant reduction of Ly6G+ TANs. (A-C) Immunohistochemistry for CHI3L1 (A), CHI3L3 (B) and the PMN-MDSC/TAN marker Ly6G (C) on primary tumor sections from untreated, anti-PD-1-, chitin- and chitin + anti-PD-1-treated 4T1 and 66cl4 tumor-bearing mice at 5 w p.i. (n = 16 for all groups; 4 slides with 4 images per slide). Dashed inserts highlight stained tissue at a larger magnification. Black scale bars = 200 µm, red scale bars = 50 μm. Data are presented as the means +/- SEM. *: P < 0.05, ***: P < 0.001. [file 13058_2024_1815_MOESM7_ESM.tif]

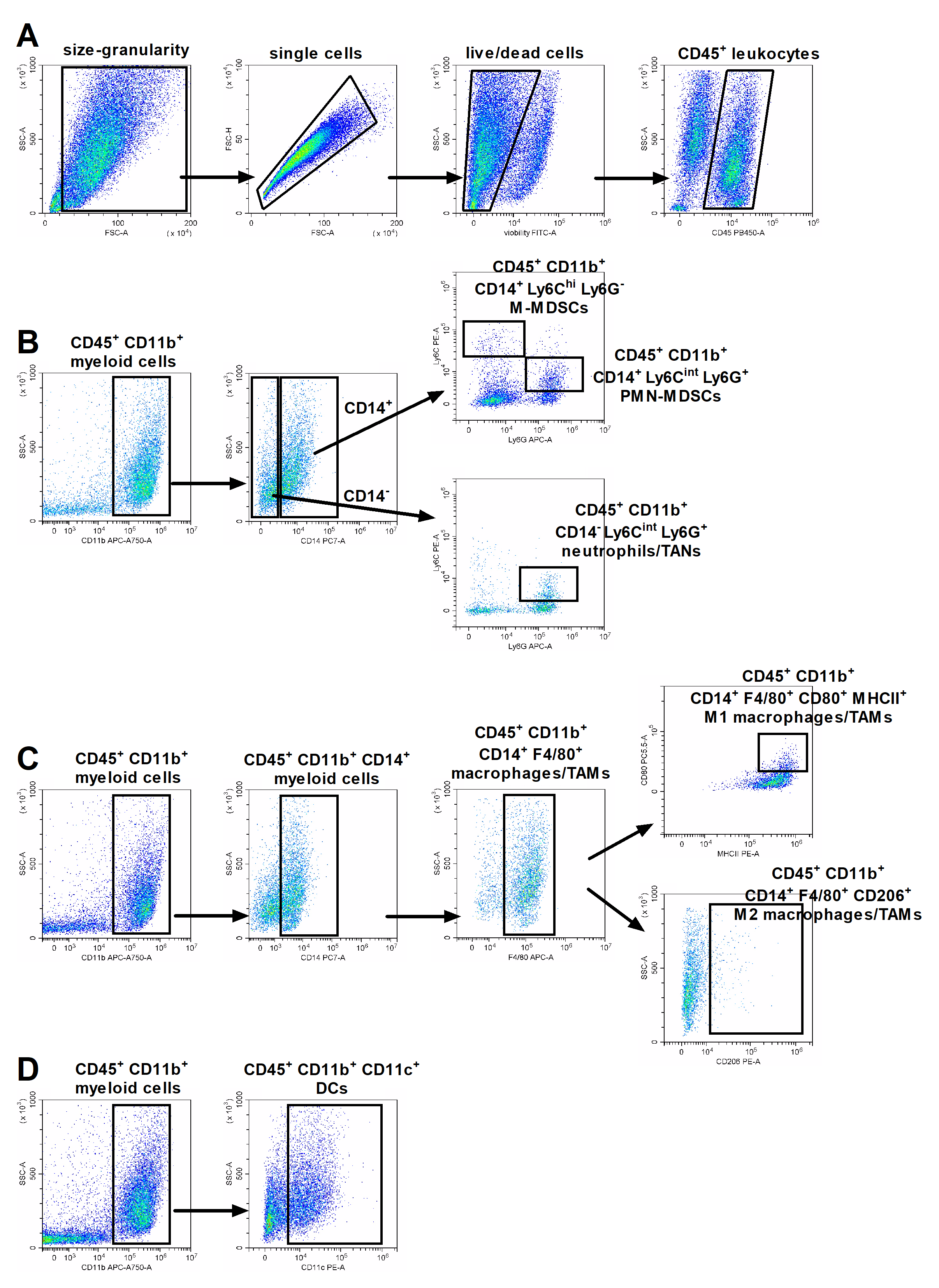

Supplement: Supplementary file 8 — Additional file 8: Figure S6. Gating strategy for the flow cytometric immunophenotyping of myeloid cell types. (A) Gating of CD45+ leukocytes after excluding doublets and debris, also applied in the panels prior to gating of specific immune cell types. (B) Gating of CD45+ CD11b+ myeloid cells for subsequent gating of CD45+ CD11b+ CD14+ and CD45+ CD11b+ CD14- myeloid cells. The CD45+ CD11b+ CD14+ myeloid cells were further subdivided into CD45+ CD11b+ CD14+ Ly6Cint Ly6G+ PMN-MDSCs and CD45+ CD11b+ CD14+ Ly6Chi Ly6G- M-MDSCs, and the CD45+ CD11b+ CD14- myeloid cells were further gated towards CD45+ CD11b+ CD14- Ly6Cint Ly6G+ neutrophils/TANs. (C) Gating of CD45+ CD11b+ myeloid cells for subsequent gating of CD45+ CD11b+ CD14+ myeloid cells and CD45+ CD11b+ CD14+ F4/80+ macrophages/TAMs. Applying CD80 and MHCII allowed additional gating of CD45+ CD11b+ CD14+ F4/80+ CD80+ MHCII+ M1 macrophage/TAM subtypes and applying CD206 allowed gating of CD45+ CD11b+ CD14+ F4/80+ CD206+ M2 macrophage/TAM subtypes. (D) Gating of CD45+ CD11b+ myeloid cells for subsequent gating of CD45+ CD11b+ CD11c+ DCs. [file 13058_2024_1815_MOESM8_ESM.tif]

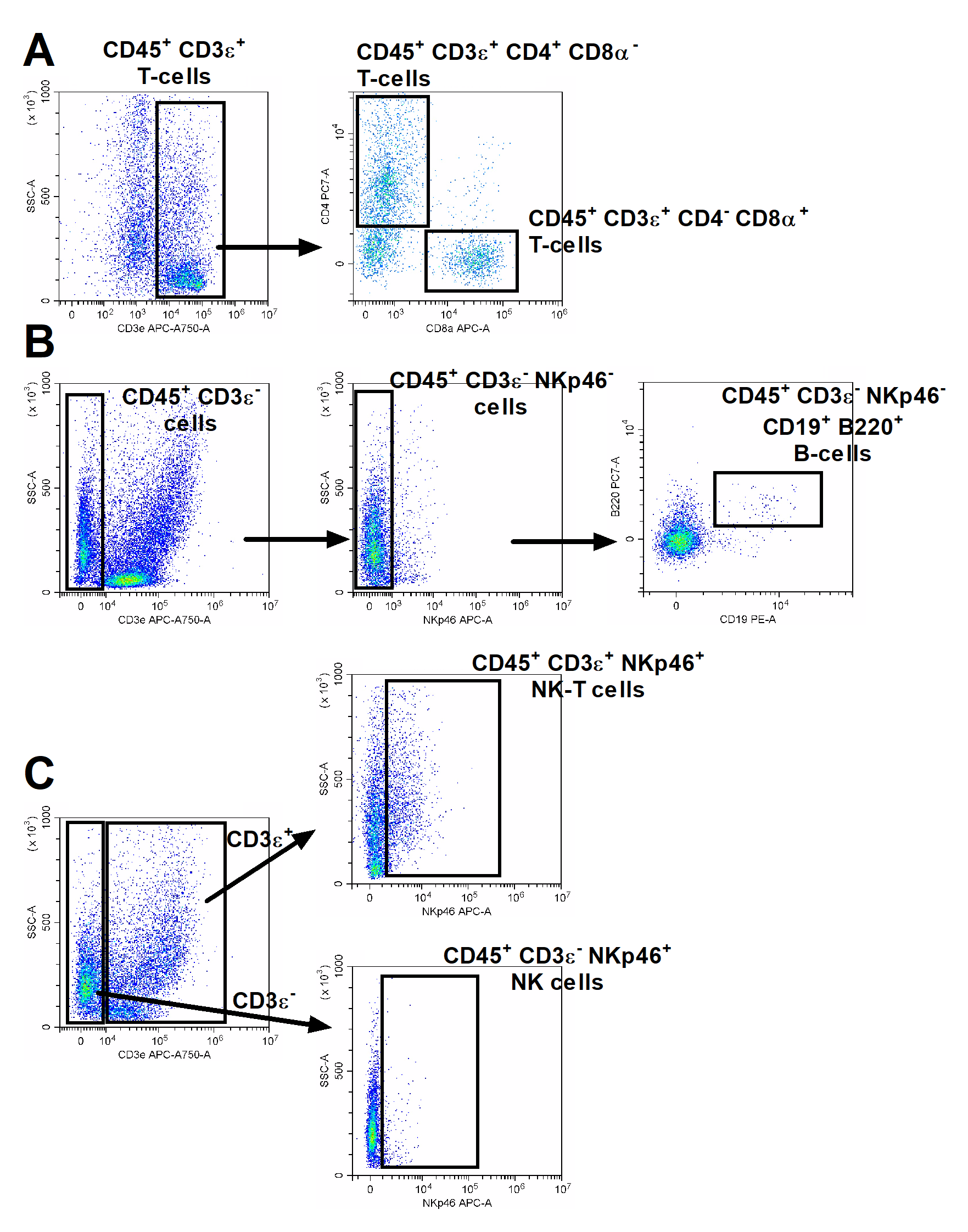

Supplement: Supplementary file 9 — Additional file 9: Figure S7. Gating strategy for the flow cytometric immunophenotyping of lymphocytic cell types. (A) Gating of CD45+ CD3ε+ T-cells for subsequent gating of CD45+ CD3ε+ CD4+ CD8α- and CD45+ CD3ε+ CD4- CD8α+ T-cell subtypes. (B) Gating of CD45+ CD3ε- cells for subsequent gating of CD45+ CD3ε- NKp46- cells and CD45+ CD3ε- NKp46- CD19+ B220+ B-cells. (C) Gating of CD45+ CD3ε+ T-cells for subsequent gating of CD45+ CD3ε+ NKp46+ NK-T cells. Gating of CD45+ CD3ε- cells was also applied for subsequent gating of CD45+ CD3ε- NKp46+ NK cells. [file 13058_2024_1815_MOESM9_ESM.tif]

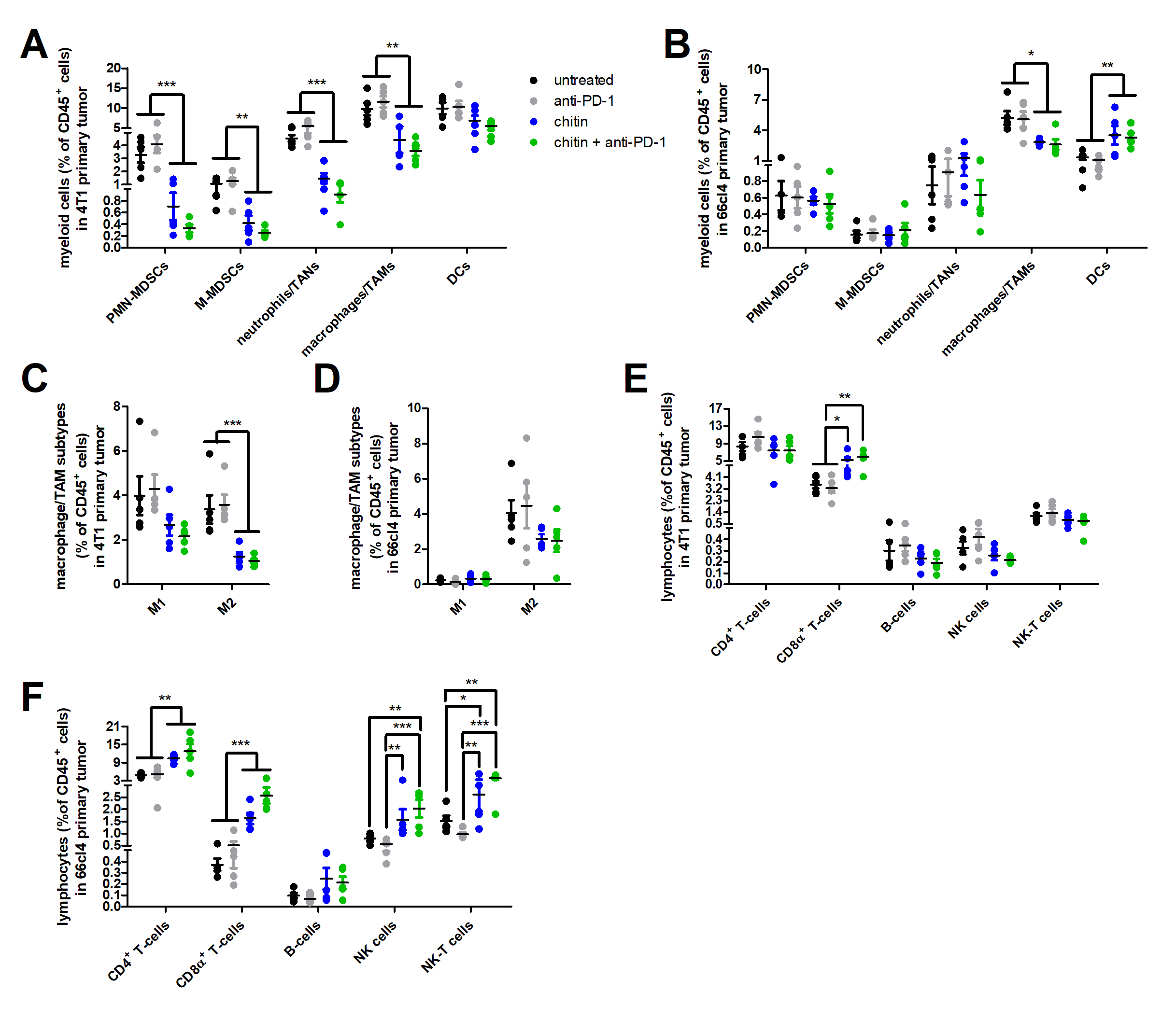

Supplement: Supplementary file 10 — Additional file 10: Figure S8. Flow cytometric data from figure 5 shown as % of CD45+ cells. (A,B) Percentage of myeloid subpopulations (including PMN-MDSCs, M-MDSCs, TANs, TAMs and DCs) within the CD45+ leukocyte population of untreated and treated 4T1 (A) and 66cl4 primary tumors (B). (C,D) Percentage of M1 and M2 TAM subtypes within the CD45+ leukocyte population of untreated and treated 4T1 (C) and 66cl4 primary tumors (D). (E,F) Percentage of lymphocytic subpopulations (including CD4+ and CD8α+ T-cells, B-cells, NK cells and NK-T cells) within the CD45+ leukocyte population of untreated and treated 4T1 (E) and 66cl4 primary tumors (F). Data are presented as the means +/- SEM with n =5 for all groups. *: P < 0.05, **: P < 0.01, ***: P < 0.001. [file 13058_2024_1815_MOESM10_ESM.tif]

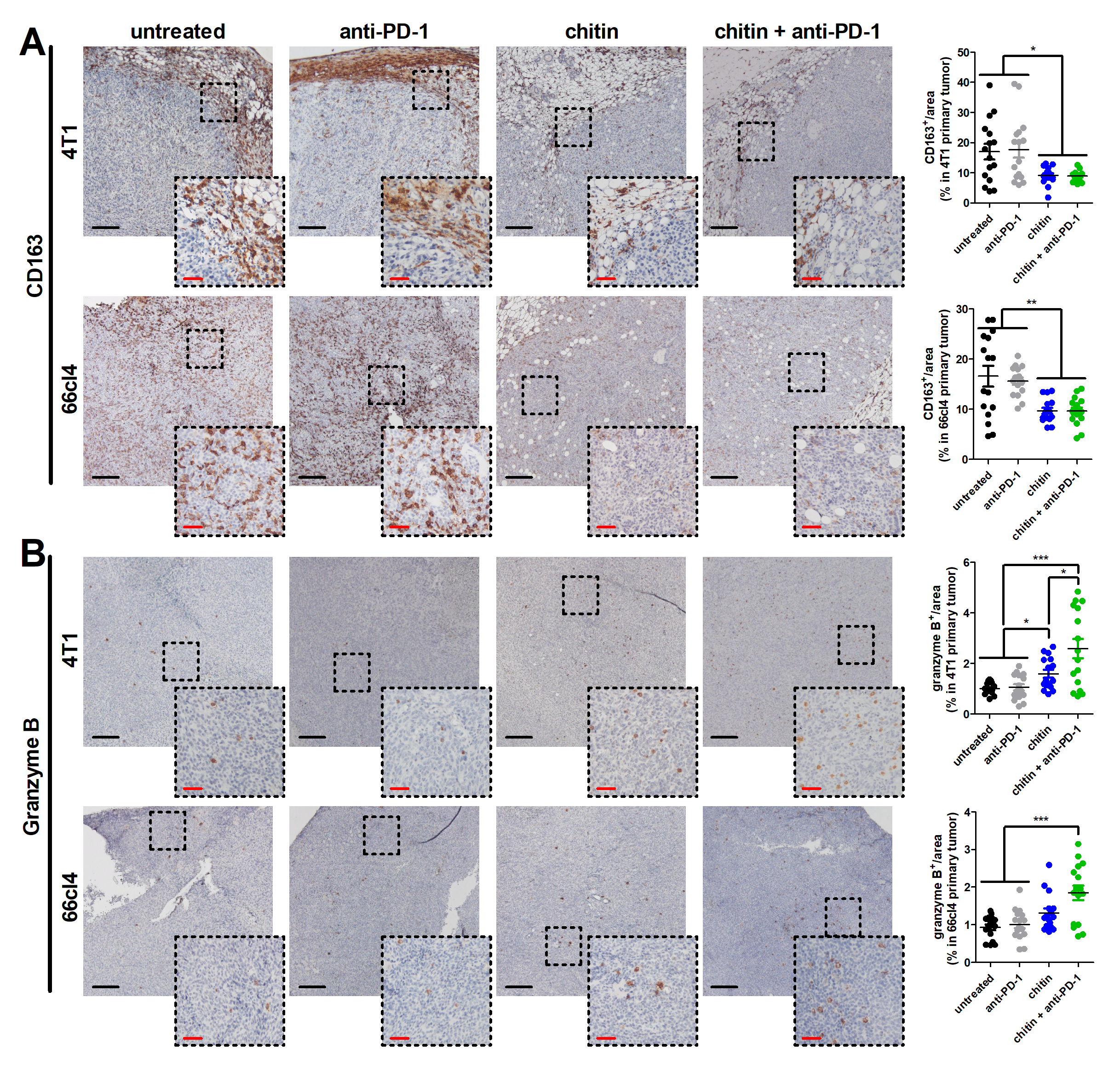

Supplement: Supplementary file 11 — Additional file 11: Figure S9. Immunohistochemistry confirms the M2 TAM reduction and enhanced lymphocytic activation upon chitin either with or without anti-PD-1 treatment in 4T1 and 66cl4 primary tumors. (A,B) Immunohistochemistry for the M2 TAM subtype marker CD163 (A) and the lymphocytic activation marker granzyme B (B) on primary tumor sections from untreated, anti-PD-1-, chitin- and chitin + anti-PD-1-treated 4T1 and 66cl4 tumor-bearing mice at 5 w p.i. (n = 16 for all groups; 4 slides with 4 images per slide). Dashed inserts highlight stained tissue at a larger magnification. Black scale bars = 200 µm, red scale bars = 50 μm. Data are presented as the means +/- SEM. *: P < 0.05, **: P < 0.01, ***: P < 0.001. [file 13058_2024_1815_MOESM11_ESM.tif]

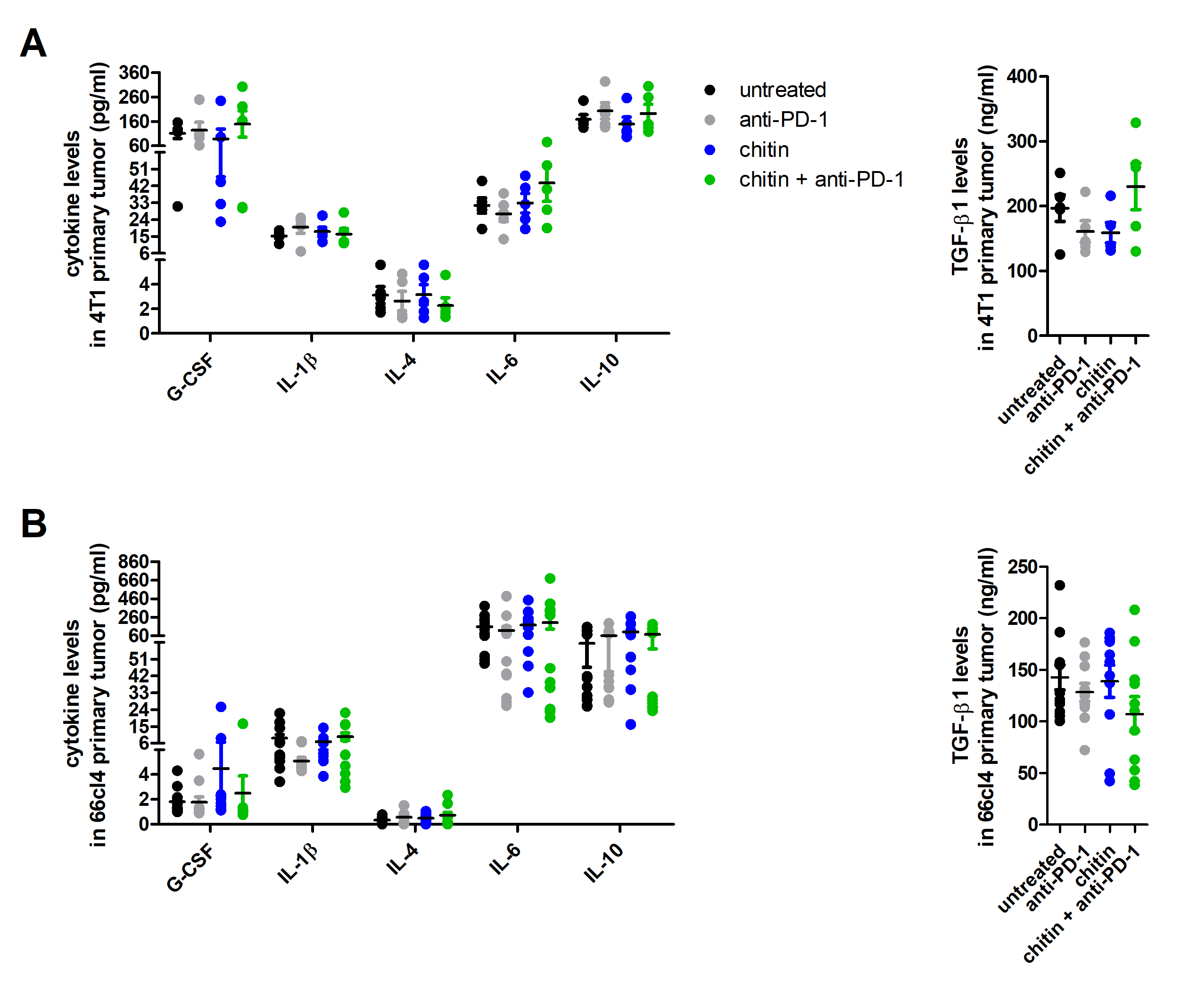

Supplement: Supplementary file 12 — Additional file 12: Figure S10. Six of the investigated cytokines remain unchanged upon chitin with or without anti-PD-1 treatment in 4T1 and 66cl4 primary tumors. (A,B) Levels for G-CSF, IL-1β, IL-4, IL-6, IL-10 and TGF-β1 in primary tumor lysates from untreated, anti-PD-1-, chitin- and chitin + anti-PD-1-treated 4T1 (A) and 66cl4 tumor-bearing mice (B) at 5 w p.i. (n = 5 for all groups in the 4T1-based model; n = 11 for all groups in the 66cl4-based model). Data are presented as the means +/- SEM. [file 13058_2024_1815_MOESM12_ESM.tif]

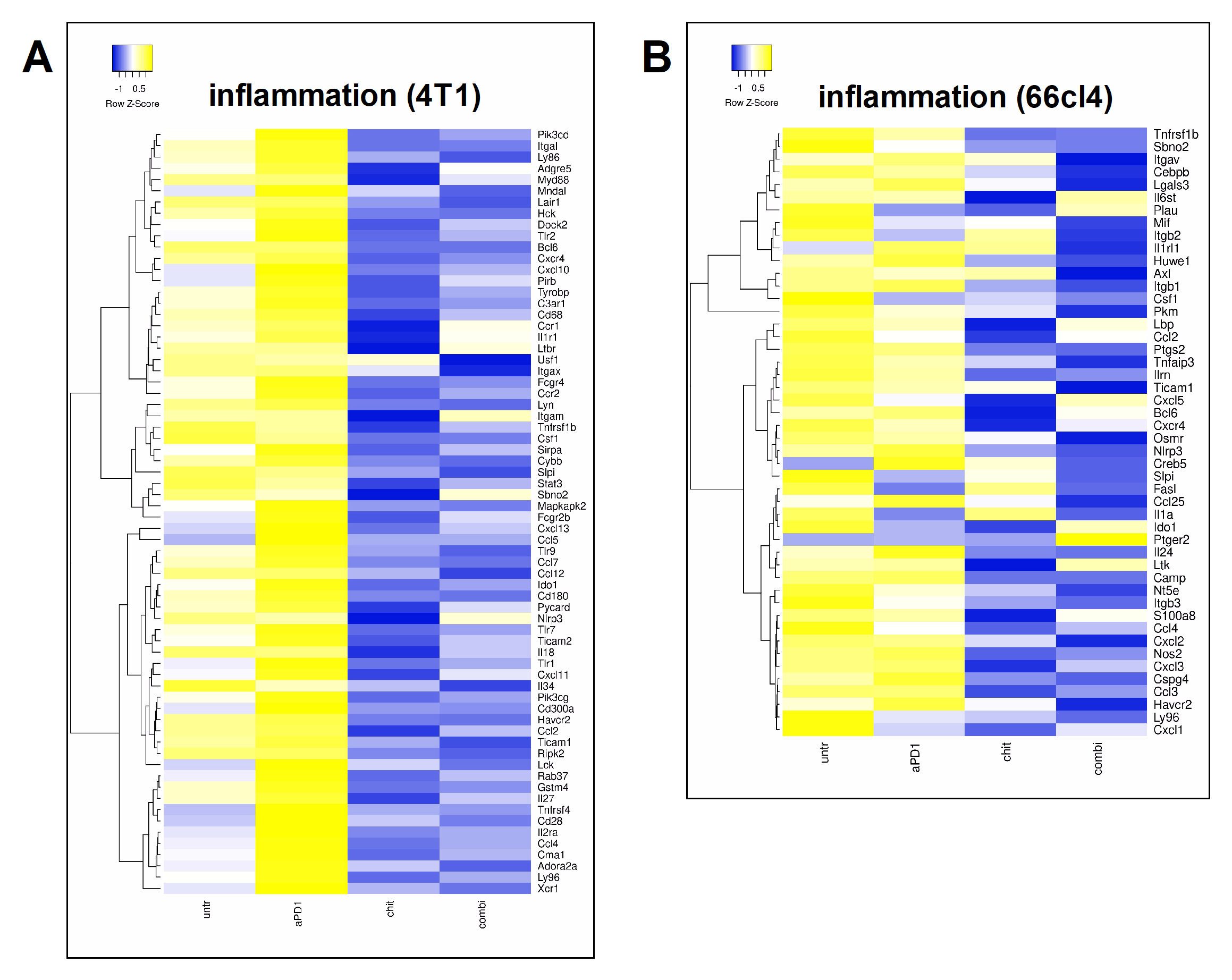

Supplement: Supplementary file 13 — Additional file 13: Figure S11. Inflammation-related gene expression levels corroborate the reduced immunosuppressive activity upon chitin treatment in 4T1 and 66cl4 primary tumors. (A,B) Heatmaps showing normalized expression levels of selected genes associated with inflammation in 4T1 (A) and 66cl4 primary tumors (B) at 5 w p.i. derived from untreated, anti-PD-1, chitin- and chitin + anti-PD-1-treated tumor-bearing mice. Mean normalized expression levels were calculated based on normalized expression levels in 4 or 5 primary tumor samples from each treatment group and for each model. Selection of the genes was based on gene lists from the NanoString Mouse PanCancer Immune Profiling Panel. Pearson distance was used for hierarchical clustering. [file 13058_2024_1815_MOESM13_ESM.tif]

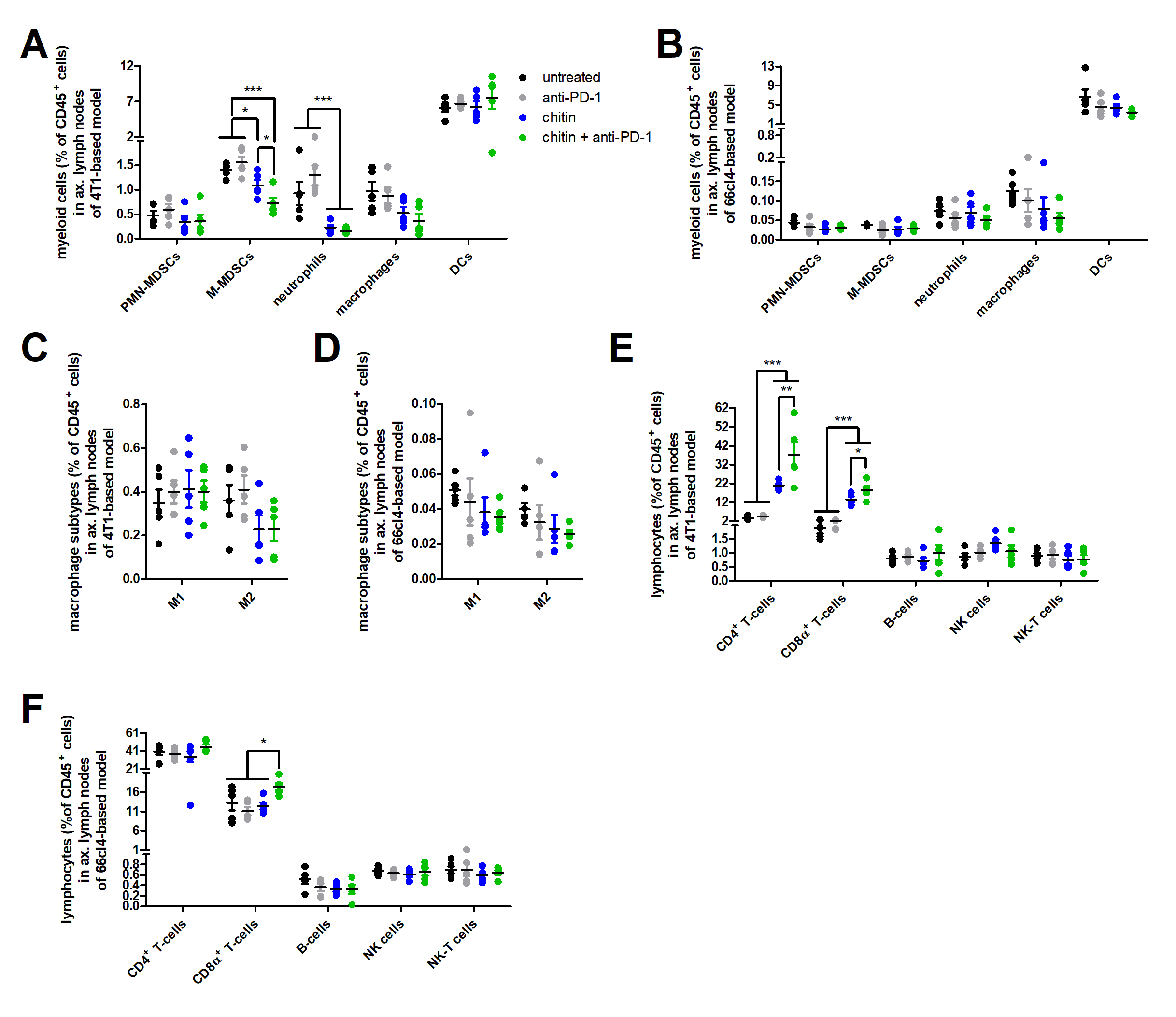

Supplement: Supplementary file 14 — Additional file 14: Figure S12. Flow cytometric data from figure 7 shown as % of CD45+ cells. (A,B) Percentage of myeloid subpopulations (including PMN-MDSCs, M-MDSCs, neutrophils, macrophages and DCs) within the CD45+ leukocyte population of axillary lymph nodes derived from the untreated and treated 4T1- (A) and 66cl4-based model (B). (C,D) Percentage of M1 and M2 macrophage subtypes within the CD45+ leukocyte population of axillary lymph nodes derived from the untreated and treated 4T1- (C) and 66cl4-based model (D). (E,F) Percentage of lymphocytic subpopulations (including CD4+ and CD8α+ T-cells, B-cells, NK cells and NK-T cells) within the CD45+ leukocyte population of axillary lymph nodes derived from the untreated and treated 4T1- (E) and 66cl4-based model (F). Data are presented as the means +/- SEM with n = 5 for all groups. *: P < 0.05, **: P < 0.01, ***: P < 0.001. [file 13058_2024_1815_MOESM14_ESM.tif]

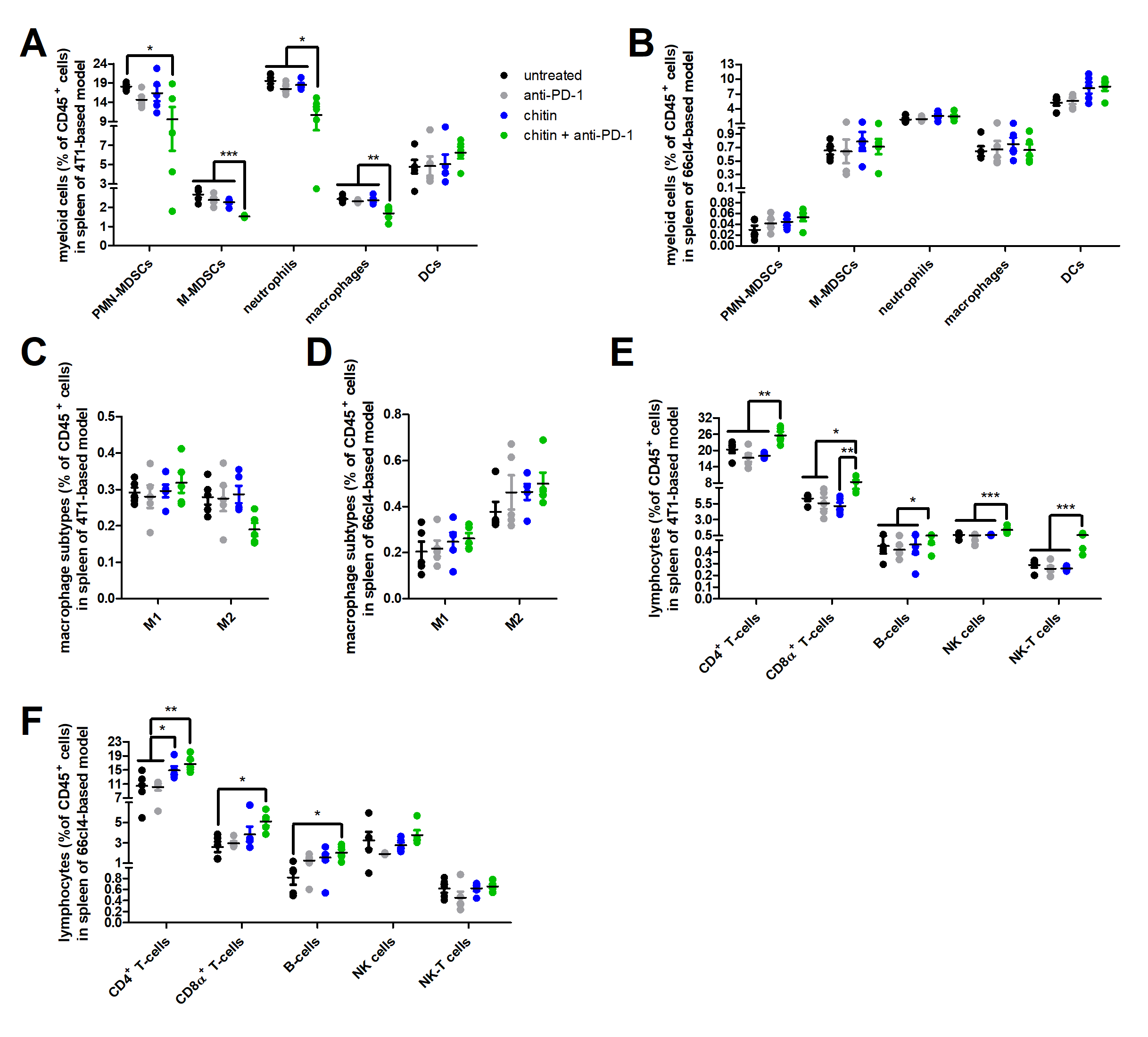

Supplement: Supplementary file 15 — Additional file 15: Figure S13. Flow cytometric data from figure 8 shown as % of CD45+ cells. (A,B) Percentage of myeloid subpopulations (including PMN-MDSCs, M-MDSCs, neutrophils, macrophages and DCs) within the CD45+ leukocyte population of spleens derived from the untreated and treated 4T1- (A) and 66cl4-based model (B). (C,D) Percentage of M1 and M2 macrophage subtypes within the CD45+ leukocyte population of spleens derived from the untreated and treated 4T1- (C) and 66cl4-based model (D). (E,F) Percentage of lymphocytic subpopulations (including CD4+ and CD8α+ T-cells, B-cells, NK cells and NK-T cells) within the CD45+ leukocyte population of spleens derived from the untreated and treated 4T1- (E) and 66cl4-based model (F). Data are presented as the means +/- SEM with n = 5 for all groups. *: P < 0.05, **: P < 0.01, ***: P < 0.001. [file 13058_2024_1815_MOESM15_ESM.tif]

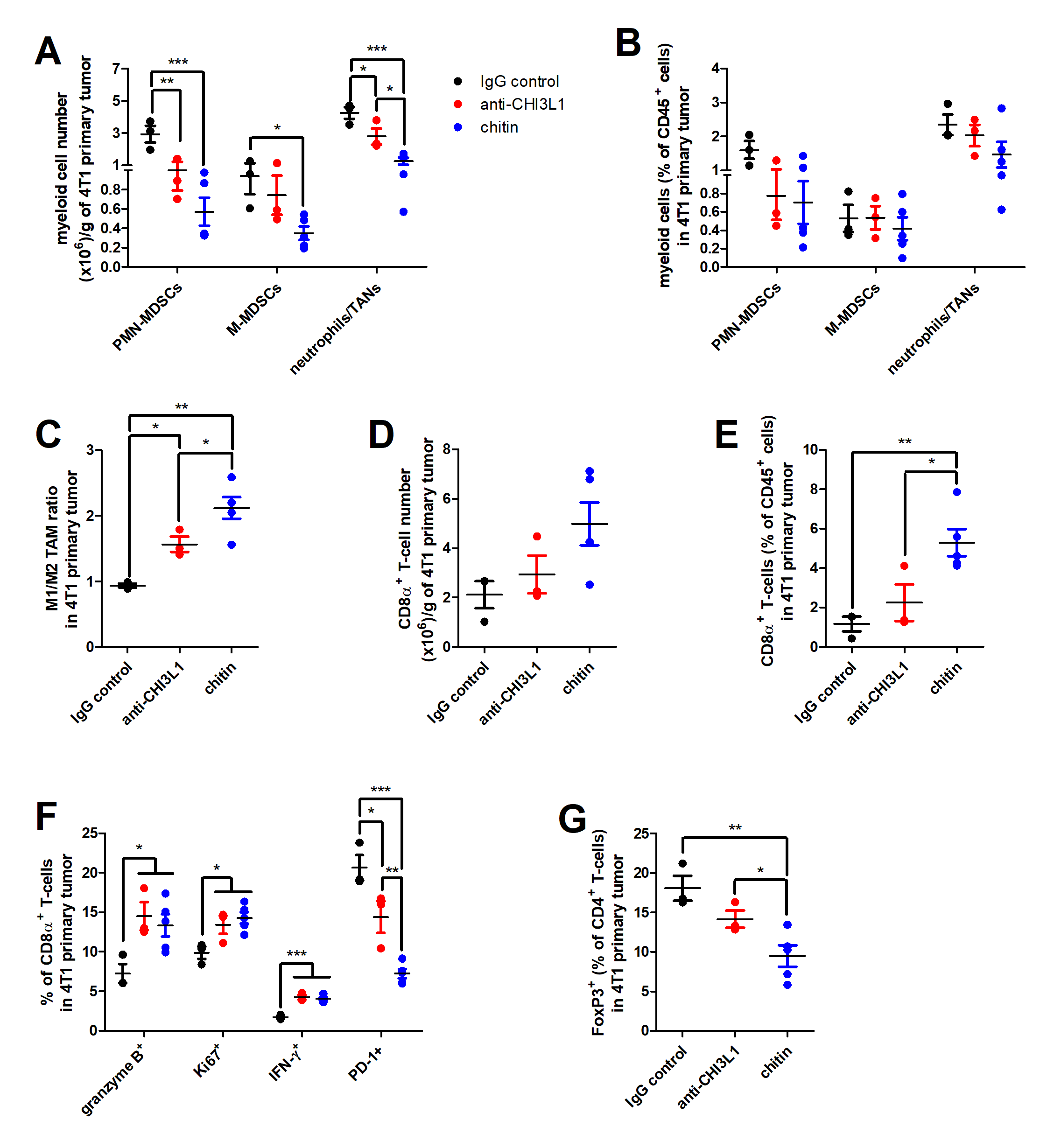

Supplement: Supplementary file 16 — Additional file 16: Figure S14. Chitin reduces immunosuppression and increases CD8α+ T-cells and their activation to a higher extent than anti-CHI3L1 treatment in the 4T1-based intraductal model. (A-F) Primary tumors were isolated from the IgG control-, anti-CHI3L1- and chitin-treated 4T1-based model at 5 w p.i. and processed into a single cell suspension for flow cytometric immunophenotyping (n = 3 for the IgG control and anti-CHI3L1 group, n = 5 for the chitin group). (A,B) Number of myeloid subpopulations (including PMN-MDSCs, M-MDSCs and TANs) per gram of untreated and treated primary tumor (A) and percentage of these myeloid subpopulations within the CD45+ leukocyte population of untreated and treated primary tumors (B). (C) Calculated M1/M2 TAM ratio in untreated and treated primary tumors. (D,E) Number of CD8α+ T-cells per gram of untreated and treated primary tumor (D) and percentage of these CD8α+ T-cells within the CD45+ leukocyte population of untreated and treated primary tumors (E). (F) Percentage of granzyme B+, Ki67+, IFN-γ+ and PD-1+ cells within the primary tumor CD8α+ T-cell population in the untreated and treated 4T1-based model. (G) Percentage of FoxP3+ cells within the primary tumor CD4+ T-cell population in the untreated and treated 4T1-based model. Data are presented as the means +/- SEM. *: P < 0.05, **: P < 0.01, ***: P < 0.001. [file 13058_2024_1815_MOESM16_ESM.tif]

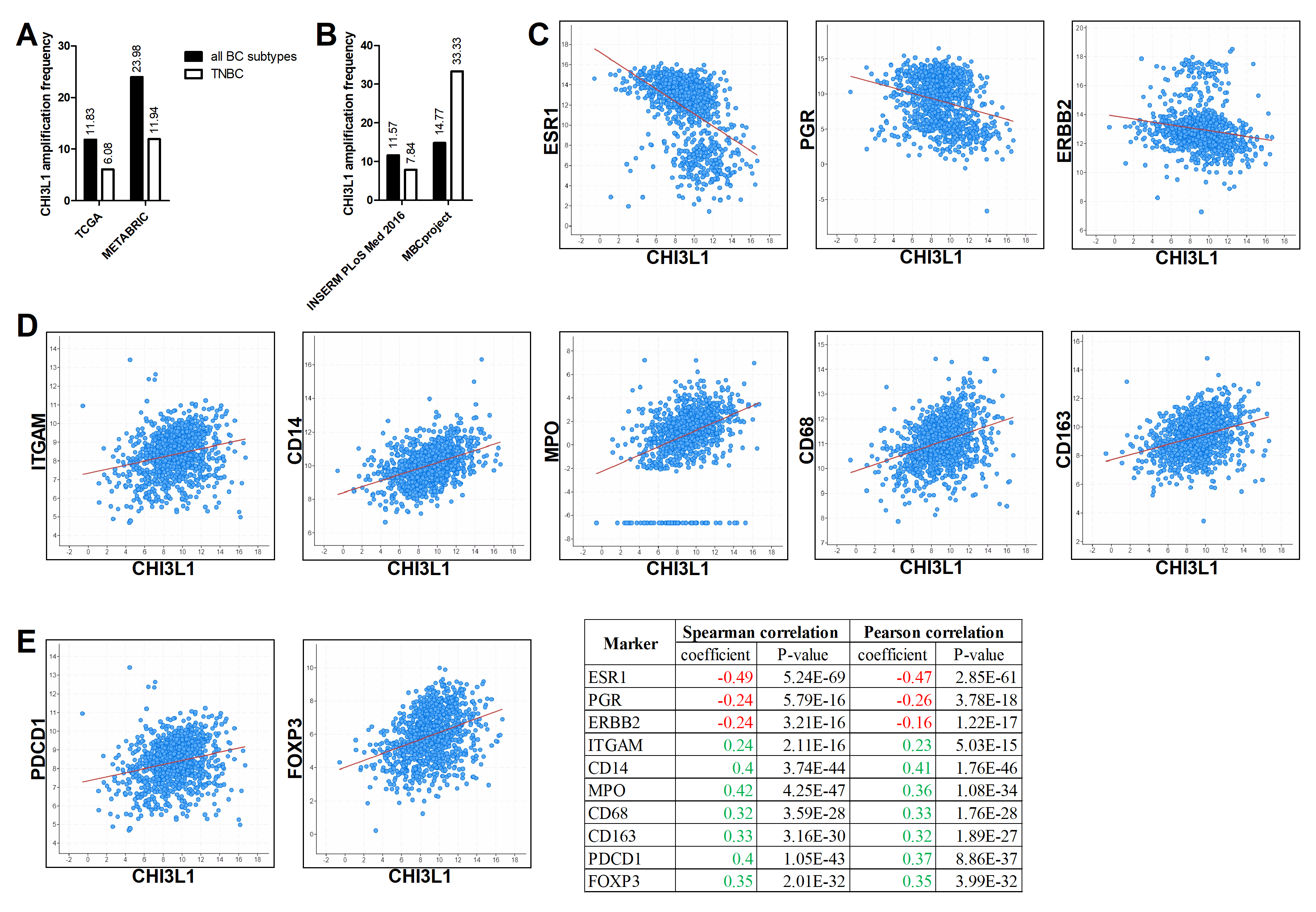

Supplement: Supplementary file 17 — Additional file 17: Figure S15. Publicly available data from BC patients correlate human CHI3L1 expression with hormonal and immune cell markers. (A,B) Amplification frequency of CHI3L1 in primary breast tumors according to the TCGA and METABRIC database (A), and metastatic BC according to a published INSERM study and the MBCproject database (B), both for all BC subtypes and TNBC. (C) Negative correlation between expression of CHI3L1 and hormone receptors ER, PR and HER2/ERBB2 based on the TCGA database. (D) Positive correlation between expression of CHI3L1 and myeloid cell markers ITGAM, CD14, MPO, CD68 and CD163 based on the TCGA database. (E) Positive correlation between expression of CHI3L1 and immune checkpoint marker PDCD1 as well as T-reg marker FOXP3 based on the TCGA database. [file 13058_2024_1815_MOESM17_ESM.tif]

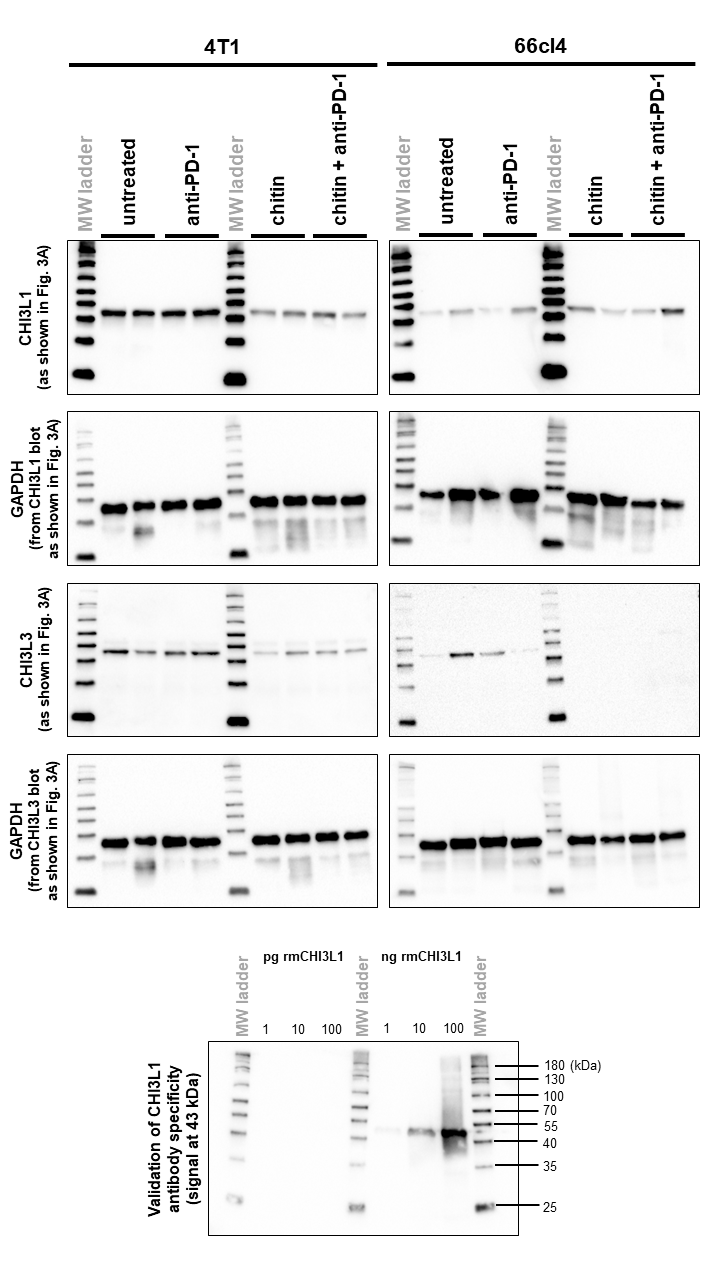

Supplement: Supplementary file 18 — Additional file 18: Figure S16. Full western blot images supporting the cropped images used in figure 3A and the specificity of the used CHI3L1 antibody. Uncropped blot images for CHI3L1, CHI3L3 and GAPDH loading controls in primary tumor lysates from untreated, anti-PD-1-, chitin- and chitin + anti-PD-1-treated 4T1 and 66cl4 tumor-bearing mice at 5 w p.i., supporting the cropped images in figure 3A. CHI3L1 antibody specificity was validated by western blot, showing a signal at 43 kDa with increasing intensity starting from 1 ng to 100 ng dose of rmCHI3L1. [file 13058_2024_1815_MOESM18_ESM.tif]

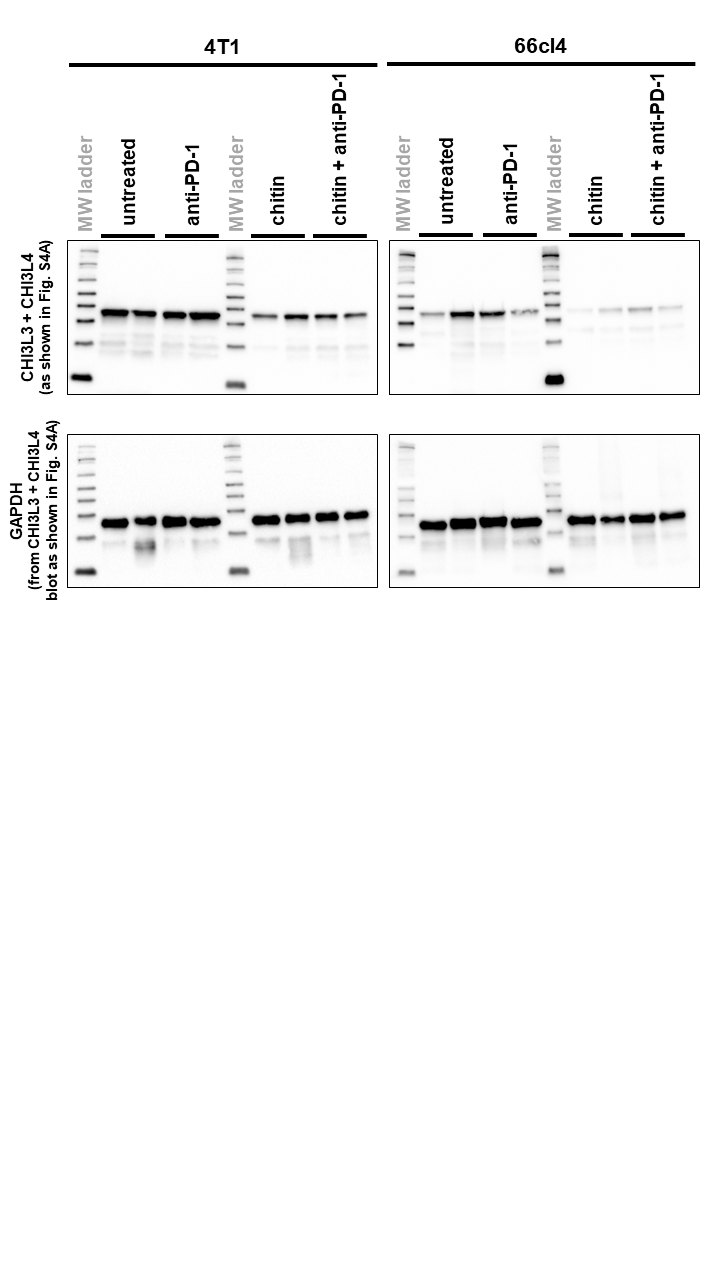

Supplement: Supplementary file 19 — Additional file 19: Figure S17. Full western blot images supporting the cropped images used in figure S4A. Uncropped blot images for CHI3L3 + CHI3L4 and GAPDH loading control in primary tumor lysates from untreated, anti-PD-1-, chitin- and chitin + anti-PD-1-treated 4T1 and 66cl4 tumor-bearing mice at 5 w p.i., supporting the cropped images in figure S4A. [file 13058_2024_1815_MOESM19_ESM.tif]
