# Supplementary material for: Chitin-mediated blockade of chitinase-like proteins reduces tumor immunosuppression, inhibits lymphatic metastasis and enhances anti-PD-1 efficacy in complementary TNBC models
Source: Breast Cancer Res. 2024 Apr 11;26:63. doi: 10.1186/s13058-024-01815-8 (PMC11007917; doi:10.1186/s13058-024-01815-8)
Supplement: Supplementary file 2 — Additional file 2: Table S1. Primary antibodies used for immunohistochemistry. [file 13058_2024_1815_MOESM2_ESM.pdf]

Table S1. Primary antibodies used for immunohistochemistry.

Target	Host species	Clone	Dilution	Antigen retrieval	Supplier
CHI3L1	Rabbit	EPR23891-162	1:800	Tris EDTA pH 9	Abcam
CHI3L3	Rabbit	EPR21248	1:10000	Tris EDTA pH 9	Abcam
Ly6G	Rat	1A8	1:1000	Citrate pH 6	BioLegend
p-Stat3	Rabbit	D3A7	1:400	Tris EDTA pH 9	Cell Signaling Technology
MPO	Rabbit	Polyclonal	1:2000	Citrate pH 6	Agilent
CD163	Rabbit	EPR19518	1:500	Citrate pH 6	Abcam
Granzyme B	Rabbit	Polyclonal	1:1000	Citrate pH 6	Abcam
F4/80	Rat	Cl:A3-1	1:100	Citrate pH 6	BioRad
LYVE-1	Rabbit	Polyclonal	1:400	Citrate pH 6	Abcam
