# Supplementary material for: Chitin-mediated blockade of chitinase-like proteins reduces tumor immunosuppression, inhibits lymphatic metastasis and enhances anti-PD-1 efficacy in complementary TNBC models
Source: Breast Cancer Res. 2024 Apr 11;26:63. doi: 10.1186/s13058-024-01815-8 (PMC11007917; doi:10.1186/s13058-024-01815-8)
Supplement: Supplementary file 3 — Additional file 3: Table S2. Fluorophore-conjugated antibodies used for flow cytometric immunophenotyping. [file 13058_2024_1815_MOESM3_ESM.pdf]

Table S2. Fluorophore-conjugated antibodies used for flow cytometric immunophenotyping.

Target	Fluorophore	Clone	Dilution	Supplier
CD45	VioBlue	REA737	1:50	Miltenyi Biotec
CD11b	APC-Vio770	REA592	1:50	Miltenyi Biotec
CD14	PE-Vio770	REA934	1:50	Miltenyi Biotec
Ly6C	PE	REA796	1:50	Miltenyi Biotec
Ly6G	APC	REA526	1:50	Miltenyi Biotec
F4/80	APC	REA126	1:50	Miltenyi Biotec
CD206	PE	C068C2	1:40	BioLegend
MHC II	PE	REA813	1:50	Miltenyi Biotec
CD80	PerCP-Vio700	REA983	1:50	Miltenyi Biotec
CD11c	PE	REA754	1:50	Miltenyi Biotec
CD3ε	APC-Vio770	REA606	1:50	Miltenyi Biotec
CD4	PE-Vio770	REA604	1:50	Miltenyi Biotec
FoxP3	PE	REA788	1:50	Miltenyi Biotec
CD8α	APC	REA601	1:50	Miltenyi Biotec
Granzyme B	PE	REA226	1:50	Miltenyi Biotec
Ki67	PE	REA183	1:50	Miltenyi Biotec
IFN-γ	PE	REA638	1:50	Miltenyi Biotec
PD-1	PerCP-Vio700	REA802	1:50	Miltenyi Biotec
CD19	PE	REA749	1:50	Miltenyi Biotec
CD45R (B220)	PE-Vio770	REA755	1:50	Miltenyi Biotec
CD335 (NKp46)	APC	REA815	1:50	Miltenyi Biotec
PDPN	APC	8.1.1	1:80	BioLegend
